# AI as a Helper: Leveraging Generative AI Tools Across Common Parts of the Creative Process

**DOI:** 10.3390/jintelligence13050057

**Published:** 2025-05-20

**Authors:** Sudapa Chompunuch, Todd Lubart

**Affiliations:** 1 Institute for Knowledge and Innovation Southeast Asia (IKI-SEA), Bangkok University, Pathum Thani 12120, Thailand; 2 Laboratoire de Psychologie et d’Ergonomie Appliquée (LaPEA), Université Paris Cité and Univ Gustave Eiffel, F-92100 Boulogne-Billancourt, France; todd.lubart@u-paris.fr

**Keywords:** generative artificial intelligence (GenAI), problem identification and framing, generating ideas, evaluating ideas, deploying and implementing ideas, use cases, common part of the creative process

## Abstract

This study explores Generative Artificial Intelligence (GenAI) applications in creativity. We identify the four most common parts of the creative process: (1) Problem Identification and Framing, (2) Generating ideas, (3) Evaluating ideas, and (4) Deploying and Implementing ideas. We map Generative AI systems into this common part of the creative process. By positioning GenAI as a supportive “AI as a helper”, we propose a structured framework that identifies specific GenAI tools and their capabilities within each common part of the creative process. Through the analysis and demonstration of use cases, this study demonstrates how Generative AI systems facilitate problem identification, generate novel ideas, evaluate ideas, and enhance implementation. We also propose the criteria for evaluating these GenAI systems for each part of the process. Moreover, this study provides insights for researchers and practitioners who are seeking to enhance GenAI’s creative capabilities and human creativity. This study concludes with a discussion of the implications of these illustrative use cases and suggests directions for future research to further advance the use of GenAI.

## 1. Introduction

The rapid advancements of Generative Artificial Intelligence (GenAI) have significantly impacted various fields, especially in the creative industries. According to Generative AI Examples from Google Cloud (2023), GenAI is defined as “the use of AI to create new content, like text, images, music, audio, and videos”, utilizing a machine learning (ML) model “to learn the patterns and relationships in a dataset of human-created content” and then “applying the learned patterns to generate new content”. Thus, GenAI represents an evolution in AI, emphasizing the creation of human-like content through advanced machine learning algorithms and neural networks, generating original outputs from human prompts. This technology is changing how many industries work by enhancing human-AI collaboration and providing tools to augment human capabilities, including automated content creation and decision-making support. GenAI systems such as ChatGPT and DALL-E are increasingly used to augment human creativity, especially for idea generation. This has led to improved efficiency and innovation in various fields (Oluwagbenro 2024; Young et al. 2024).

The creative process usually involves various stages, such as preparation, idea generation, selection, and implementation (Chompunuch 2023). Generative AI (GenAI) has significantly transformed the creative landscape, enhancing various stages of the creative process through intelligent assistance. From generating novel ideas to implementing those ideas, AI systems are reshaping how creativity is approached, making it more dynamic and accessible. This transformation is evident across multiple domains, including design (As and Basu 2021; Chandrasekera et al. 2024), art (Egon et al. 2023; Sinha et al. 2024), and music (Hirawata and Otani 2024) where AI acts as a co-creator or partner (Lubart 2005). Human collaboration with Generative Artificial Intelligence (GenAI) in creative tasks is characterized by a synergistic relationship, where AI acts as a co-creator with human artists and thinkers. This synergy is particularly evident in scenarios where AI applications are intentionally designed to enhance human creativity, aiding in the invention of new practices. Including AI in creative workflows fosters balanced cooperation that bolsters the creative process, adheres to ethical norms, and maintains human values (Vinchon et al. 2023; O’Toole and Horvát 2024).

Moreover, GenAI has significantly democratized creativity by reducing the expertise required for creative tasks. Individuals who may lack traditional creative skills can now use AI-assisted tools to articulate their ideas and emotions, making creativity more accessible to a broader audience. In this way, GenAI is a powerful facilitator of human expression, enabling a more diverse range of people to participate in creative activities (Tigre Moura 2023). However, integrating GenAI into the creative process creates challenges. Despite the increasing adoption of GenAI in creative processes, there is a lack of a comprehensive framework that systematically maps GenAI functions to specific stages of the creative process, making it challenging for practitioners to effectively integrate these tools.

This study addresses two research questions:

1. How can GenAI facilitate common steps of the creative process?
2. What are the main benefits and limitations of integrating GenAI tools?

This study positions Generative AI (GenAI) as an “AI as a helper” to highlight the collaborative dynamic between human creativity and machine capabilities. By representing AI as a supportive partner rather than a replacement, this perspective maintains human agency and judgment, allowing individuals to shape and enhance AI outputs. Consequently, “AI as a helper” fosters a more balanced, ethically grounded, and adaptive creative process that harnesses both human strengths and the efficiency of machine intelligence. Through detailed analysis and key use case demonstrations, we aim to provide a structured framework that highlights GenAI’s potential benefits and applications in creative work. Our analysis encompasses stage-specific applications and essential steps in the creative process, including (1) Problem Identification and Framing, (2) Generating Ideas, (3) Evaluating Ideas, and (4) Deploying and Implementing Ideas.

This study contributes to both theory and practice by providing a comprehensive framework of GenAI assistance across the four common steps of the creative process. Our goal is to provide valuable insights to researchers and practitioners on using GenAI to enhance human creativity. This research enriches the growing understanding of GenAI and creativity, highlighting potential opportunities and challenges in the field. We conclude by discussing the implications of our findings and suggesting future research directions for integrating GenAI into creative processes.

The first section of the paper discusses the history and background of generative artificial intelligence (GenAI). The Literature Review then examines the body of research on models of the creative process, GenAI-enhanced creativity, and the justification for referring to it as an “AI helper” in the creative process. Following this, the Conceptual Framework section aligns GenAI systems with four key common steps in the creative process. The Methodology describes the research design, incorporating theoretical mapping and a demonstration-based approach. In the Use Case Analysis, examples illustrate how GenAI tools operate within four common creative steps. Moreover, the Discussion integrates findings, highlights implications, and outlines limitations. The Conclusion summarizes key contributions and suggests future research directions.

## 2. Literature Review

### 2.1. The Creative Process: Definitions and Models

The creative process is defined as a succession of thoughts and actions leading to original and appropriate productions (Lubart 2001). The literature on the creative process has extensively explored the concept of stage models for creative problem-solving. The pioneer in this field was Wallas (1926), whose seminal qualitative work conceptualized four linear stages for creativity to occur: Preparation, Incubation, Illumination, and Verification (Wallas 1926). Since then, various researchers have proposed their own stage models (see Table 1). Thus, the study of the creative process heavily relies on stage models, which have become an essential tool for researchers (Runco 2004; Sadler-Smith 2015). These models have led to the development of divergent thinking tests such as (Torrance 1974) and insight problem-solving tasks (Davidson 2003). However, these models have been criticized for assuming that creativity follows a linear process. Montag et al. (2012) argue that the creative process is a dynamic and reiterative process that requires both divergent and convergent thinking, which qualitative evidence largely supports (Montag et al. 2012; Hargadon and Bechky 2006).

From Table 1, it can be observed that the creative process typically follows four common parts. Therefore, the common parts that occur most in many creative process models often include:

1. Problem identification/problem-framing
2. Generating ideas
3. Evaluating ideas
4. Deploying ideas (Validation or Implementation)

These steps are commonly recognized as key parts of the creative process (Leone et al. 2023; Lubart 2001; Puccio and Modrzejewska-Świgulska 2022). It is important to note that these steps are not always linear and can be influenced by various internal and external factors, as well as the specific context in which creativity is being applied. The integration of Generative Artificial Intelligence (GenAI) into this process has brought new possibilities, such as enabling rapid idea generation through GenAI brainstorming tools, providing immediate feedback and critiques on ideas, prototyping, and visual synthesis. However, this integration also has challenges, including potential biases in AI-generated suggestions, difficulty in managing human oversight and creative autonomy, and overly generic or superficial outputs. Therefore, effective GenAI integration requires domain expertise, ethical judgment, and context-sensitive human interpretation.

### 2.2. GenAI in Creativity Research and Gaps in the Literature

*There are studies* emerging on GenAI-supported or GenAI-driven creativity in various domains, such as visual art (Zhou and Lee 2024), music composition (Michele Newman and Lee 2023), narrative generation (Doshi and Hauser 2024; McGuire 2024), and design (Moreau et al. 2023). In addition, Urban et al. (2024) study found that ChatGPT improves the creative problem-solving of university students. As shown in the historical timeline of GenAI, early work in computational creativity often investigated “Rule-based” approaches that generate novel artifacts under constraints. The advances in deep learning or Generative Adversarial Networks (GANs) and transformer-based models have produced a new generation of creative AI systems that can automate generating paintings, music, and design prototypes. Studies e.g., (Lubart et al. 2021; Zhang et al. 2023; Mao et al. 2024) in AI creativity now often focus on “Creative Collaboration”, how human-AI collaboration or co-creation can generate novelty using GenAI tools such as ChatGPT, Dall-E, etc.

However, although previous research has broadly acknowledged the supportive role of GenAI in creative tasks, studies did not target specific phases of creativity that benefit most from GenAI assistance. There are a few exceptions that do look at specific phases; for example, Gindert and Müller (2024) found that GenAI tools can support teams in the idea generation phase. Similarly, Wan et al. (2024) explored human-AI co-creativity in prewriting tasks, and they also identified the creative process stages, such as ideation, illumination, and implementation, but without a detailed analysis of GenAI effectiveness in each specific stage.

While the research on AI creativity expands, the gaps in the literature need to be addressed. Much of the research focuses on isolated/single-use cases, and there is a need for studies on structured frameworks for GenAI in the creative process; for instance, in this study, we identify the four common steps of the creative process: Problem identification and Framing, Generating ideas, Evaluating ideas, Deploying and Implementing idea. There is a lack of guidelines or frameworks for researchers and practitioners seeking to integrate GenAI across all creative stages. As a result, we introduce GenAI tools for each step and give examples and guidelines on how to use them.

### 2.3. Positioning “AI as a Helper”

Several authors have proposed the frameworks or levels of human-AI collaboration. There are three ways/modes of human-AI creative collaboration (Davis et al. 2015; Karimi et al. 2018; Lubart et al. 2021; Zhang et al. 2023; Mao et al. 2024). The core idea is humans, and AI contributions can range from AI as a supportive tool (often seen as a Helper, as shown in this study), AI as a partner or co-creator, and AI as a principal Creator, depending on the level of AI autonomy and involvement. These three modes emphasize how shifts in autonomy and collaborative depth can define the creative relationship between humans and AI.

- AI as a Helper (as proposed in this study): In this mode, with the least AI involvement, humans perform most of the stages, and GenAI should provide assistance or act as a supportive tool for each part of the creative process (Zhang et al. 2023).
- AI as a Partner or Co-Creator: In this mode, with moderate AI involvement, humans and AI engage in communication to exchange information (Zhang et al. 2021) and provide mutual guidance (Oh et al. 2018), and AI helps build and test the creative outcome.
- AI as a Principal Creator: In this mode, with the highest AI involvement, “AI should be a more autonomous process, with AI participating in most of the stages based on requirements or inputs provided by humans” (Zhang et al. 2023).

Therefore, in this study, we proposed “AI as a Helper” to demonstrate one mode of human-AI collaboration. AI as a Helper highlights a human-centered approach where AI is used as a helper to assist (rather than compete) in the creative process. This human-centered approach supports the concept of collaborative innovation over pure automation. Rather than framing AI as a replacement for human creativity, we emphasize the potential boost from GenAI models such as text-based ones like ChatGPT or image-based ones like Dall-e or Midjourney to enhance rather than replace the creativity of individuals and teams. Such a human-centered approach, viewing AI systems as supportive partners, the “AI as a Helper” concept adds a novel dimension to the creative interactions and encourages researchers and practitioners to gain efficiency and fresh conceptual inputs while maintaining full human control over contextual and ethical decisions.

## 3. Conceptual Framework: Mapping GenAI to the Creative Process

### 3.1. Overview of the Four Common Parts of the Creative Process

- **Problem Identification and Framing**

The first stage of the creative process, known as “Preparation” in Wallas’s (1926) model, focuses on identifying problems and framing problems (Carson 1999; Wallas 1926). Creativity is sparked by an incident or idea encountered by an individual (Doyle 1998). This stage is vital to the creative process, with research indicating that the way individuals approach problem identification and construction significantly impacts their creative output (Getzels and Csikszentmihalyi 1976). This initial phase, the most common part of the creative process, focuses on identifying, recognizing, clarifying, or reframing the core problem or question. Scholars like Rhodes (1961) and Osborn (1979) highlight the importance of articulating the problem clearly and compellingly, as this shapes all subsequent ideation and solution-building efforts (Rhodes 1961; Osborn 1979). Amabile (1983, 1988) emphasizes that how a problem is posed interacts with an individual’s intrinsic motivation and domain-relevant skills, thus influencing creative potential (Amabile 1983, 1988). Similarly, Mumford and Reiter-Palmon (1994) and Runco and Dow (1999) highlight that a sound problem-construction process leads to higher-quality, more innovative outcomes (Mumford and Reiter-Palmon 1994; Runco and Dow 1999). Reiter-Palmon and Illies (2004) provide empirical support showing that the clarity of problem-framing predicts the success of creative solutions (Reiter-Palmon and Illies 2004), while Zhang and Bartol (2010) demonstrate how leadership and contextual support can enhance this stage (Zhang and Bartol 2010). Overall, carefully defining and reframing a problem sets the foundation for productive and impactful creativity.

- **Generating ideas**

According to Wallas (1926), the second stage is “Incubation” (Osborn 1979; Shaw 1989; Sadler-Smith 2015). Once a problem is well-defined, the focus turns to idea generation, which is the “Generating ideas” part. Osborn (1953) introduced brainstorming as a core technique, underscoring the need for freely generated ideas (Osborn 1979). During the ideation part, teams will work together to find solutions to the problem that was identified in the preparation phase. This second common part of the creative process involves gathering information to exchange ideas and generate novel ideas (Paulus and Yang 2000). Ideation refers to generating new and valuable solutions for potential opportunities. This process demands effective sharing of knowledge and information among team members, along with consideration of different perspectives. Moreover, it highlights the influence of social and motivational factors on how ideas emerge (Amabile 1983, 1988) while Nemiro (2002) examines how distributed teams effectively share and refine concepts. Consequently, the team integrates and develops individual ideas to create practical and innovative solutions. Reiter-Palmon and Illies (2004) again stress that effective problem-framing fosters more productive ideation (Reiter-Palmon and Illies 2004).

- **Evaluating ideas**

During the third stage, known as the “Selection” stage, a team has to determine the best idea from a range of ideas generated in the ideation phase. As noted by Reiter-Palmon et al. (2007), this part of the creative process involves evaluating the ideas and selecting the most promising ones (Reiter-Palmon et al. 2007). Usually, the idea selection process entails assessing ideas based on specific criteria to make a final decision. While at the individual level, idea selection is an intrapersonal process (Herman and Reiter-Palmon 2011), at the team level, it becomes a more interactive and interpersonal endeavor. Although research on idea selection is relatively limited, findings suggest that teams are generally more proficient in choosing the best ideas compared to individuals (Mumford et al. 2001b), particularly when they have fewer alternatives to consider (Mumford et al. 2001a). In this research, the Evaluating of ideas part is defined as “the process of evaluating possible new ideas and selecting the best one”. The process ends with team members selecting the best available idea.

- **Deploying and Implementing Idea**

During the last stage of the creative process, “Deploying and Implementing idea”, the abstract concept is transformed into a tangible output such as an implementation plan or a prototype. According to Reiter-Palmon and Illies (2004), translating a newly formed idea into practice needs structuring and implementing the plan, which includes clear project goals and timelines for implementation. Botella highlights the provisional object or draft of deploying and implementing the idea (Botella et al. 2013). Sawyer (2012) also emphasizes the “Externalization” of the creative thinking or idea in the deploying and implementing stage. In addition, Cropley (2015) suggests the communication and validation of an idea in the last stage of the creative process.

### 3.2. Roles of GenAI in Each Part

**Figure 1:**
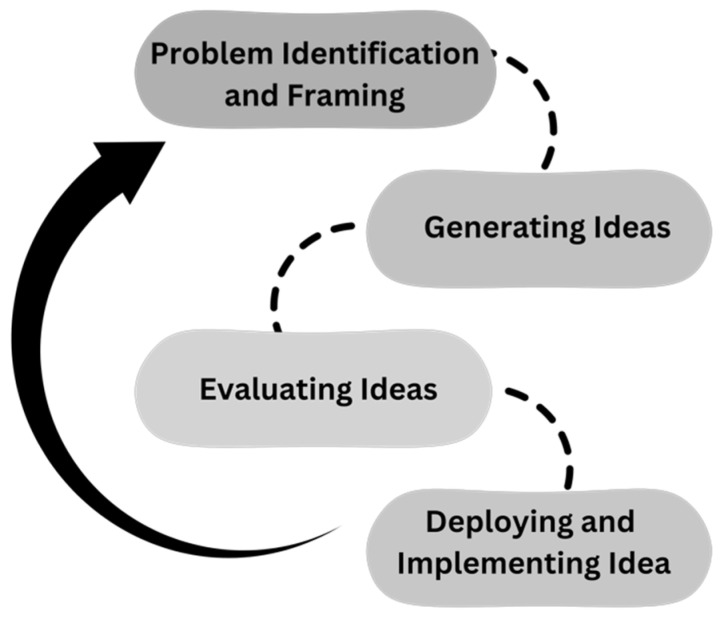
An illustration of Four common stages in creative process.

In aligning the specific capabilities of GenAI tools with four common steps of the creative process, it is recognized that GenAI does not act as a sole agent; rather, it acts as a Helper to enhance human creativity. For each common creative step: 1. Problem Identification and Framing, 2. Generating ideas, 3. Evaluating ideas, and 4. Deploying and implementing Ideas. Connecting GenAI across the steps can enhance and foster human creativity (see Figure 1).

- **Problem Identification and Framing:** GenAI language models (e.g., GPT-4) can rapidly synthesize large volumes of text to help humans understand the context and inform the definition of the problem. GenAI can also reframe and show the alternative aspect of the problem. Moreover, GenAI can help by scanning relevant information and providing quick assumptions and feedback to ensure the framing problem is comprehensive. However, GenAI may overlook and oversimplify complex issues or may introduce biased information from its training data, which leads to misleading problem definitions, so this requires validation by human expertise.
- **Generating ideas:** GenAI can help humans brainstorm and cultivate divergent thinking. It will act as an “idea catalyst”. Large language models (LLMs) or text-based models can generate a list of unconventional ideas or solutions and blend unrelated concepts in surprising ways. Image-based models (e.g., Dall-E) can generate visual pictures to spark idea generation, helping design inspiration. Also, some GenAI can be integrated into brainstorming workshops like Stormz AI, pushing the team’s ideas beyond the boundaries. Despite these advantages, GenAI-generated ideas might lack feasibility or practical value. There is also a risk of generating superficial ideas due to the absence of domain-specific expertise and contextual understanding.
- **Evaluating ideas:** Once multiple ideas are generated, GenAI can help compare and critique them. LLMs can help generate pros or opportunities and cons or constraints and suggest improvements, provide structured feedback and preliminary validation that humans can refine further. Thus, this GenAI still needs human oversight for contextual and ethical judgment. However, GenAI may lack contextual, ethical awareness and careful judgment, which leads to biased assessments. Thus, this step needs careful human oversight.
- **Deploying and implementing Idea**: GenAI text-based or image-based can generate rapid prototyping or UX/UI designs or solutions so that humans can adjust and finalize them further (e.g., Leo AI), thus speeding up idea prototyping and deployment. For example, ChatGPT can help human users write initial draft recommendations or user manuals of the product, etc. However, GenAI-generated prototypes may not fully align with user needs and practical constraints. Moreover, reliance on AI for the initial draft could lead to overlooking critical usability considerations, so human judgment is necessary to ensure that final implementations are context-appropriate, ethically sound, and address user requirements and constraints.

## 4. Methodology

In this research, we present the Selection Methodology as shown in Table 2.

This highlights the systematic approach to identifying and selecting the most potential GenAI systems for each common part of the creative process.

1. **Identify the Common Creative Steps**: (1) Problem Identification and Framing (2) Generating Ideas (3) Evaluating Ideas (4) Deploying and Implementing Idea
2. **Define relevant keywords search:** For each creative step, we identify keywords to guide the search in Table 3:
3. **Source of GenAI Tools:** We use two sources to search for GenAI tools for specific steps in the creative process. (1) Search “There is An AI for that” for Specific Tools: First, we searched for keywords on the “There is An AI for that” website (https://theresanaiforthat.com/) (accessed on 25 March 2025) for specialized GenAI Tools for specific tasks within each creative process. (2) Search “AI Insider” for Generic Tools: Additionally, we identified generic GenAI tools based on the “AI Insider” website (https://ainsider.tools/) (accessed on 25 March 2025) that can be applied across multiple creative steps, such as text-based GenAI tools like ChatGPT.
4. **List all relevant GenAI tools from both sources for each creative step**
5. **Select Top GenAI Tools Based on Step-Specific and Generic Criteria:** From the relevant GenAI lists, we then select the top 3 tools based on popularity scores that potentially meet the predefined step-specific criteria (from “There is An AI for that”) and include generic tools (from “AI Insider”) that support multiple creative steps.
6. **Use Case Problem Selection:** In this study, we choose the problem from UN Goals UN. Sustainable Development Goal 2 (SDG Goal 2): Zero Hunger. World hunger is one of the most serious global issues. The causes of world hunger include poverty, food shortages, war and conflict, climate change, poor nutrition, poor public policy and political instability, a bad economy, food waste, gender inequality, and forced migration. These factors contribute to hunger. Therefore, we have chosen this urgent global challenge as an example to highlight GenAI’s potential role in addressing such issues and to demonstrate its application across various steps of the creative process.

**Proposed Criteria to assess the effectiveness of GenAI tools for each step of the creative process**

We apply the core structure of Saaty (1980) and Davies (1994) Hierarchical Analysis by defining key criteria, scoring each criterion, and profiling results. Their “Hierarchical Analysis or Analytic Hierarchy Process” can be interpreted as a structured way of: Defining the primary goal, Breaking down that goal into major criteria (top-level dimensions), Further dividing those criteria into sub-criteria, if necessary, Weighting each criterion and sub-criterion based on its importance, Scoring alternatives (in this case, AI tools) against each criterion and Aggregating the scores to arrive at a final comparative ranking. The concept of hierarchical analysis has broad applications across different fields, including information systems (IS), where hierarchical models (e.g., analytical hierarchy process, multi-layered system designs) are commonly used (Saaty 1980).

**Criteria used to assess GenAI tools for four common steps of the creative process**

In this section, we propose criteria for assessing GenAI tools for each step of the creative process. Table 4 for problem identification and framing, Table 5 for generating ideas, Table 6 for evaluating ideas, and Table 7 for deploying and implementing ideas.

For each criterion, a five-point rating scale can be used to evaluate the output of a genAI tool.

- **1** = Very poor/Not demonstrated
- **2** = Below average/Needs improvement
- **3** = Average/Acceptable
- **4** = Good/Above average
- **5** = Excellent/Outstanding

## 5. Use Case Demonstrations and GenAI Systems Evaluation

### 5.1. Use Case 1: Problem Identification and Framing

We demonstrate how GenAI tools helped refine or reframe the problem for Sustainable Development Goal 2 (SDG Goal 2): Zero Hunger. There are 8 GenAIs for Problem Identification and Framing using the keyword “Research Assistance”, and Table 8 shows the Top 3 GenAIs for Problem Identification and Framing. The full list of 8 GenAIs can be found in Supplementary Material S (S1), and a demonstration of the Top 3 GenAIs can be found in Supplementary Material S (S2).

**Profiler of GenAI tools for Problem Identification and Framing step**

This is a hypothetical example (Table 9) of how we score the top three GenAI tools under the five criteria (Clarity, Contextual Relevance, Analytical Depth, Innovative Angle, and Ease of Use). The two researchers (authors) evaluated the performance of GenAIs according to the criteria using a five-point Likert scale. To ensure inter-rater reliability, we calculated the Spearman–Brown coefficient based on the ratings from two independent researchers. The resulting Spearman–Brown reliability coefficient was 0.90, indicating strong agreement between evaluators and confirming the consistency of judgments across the five evaluation criteria.

**Figure 2:**
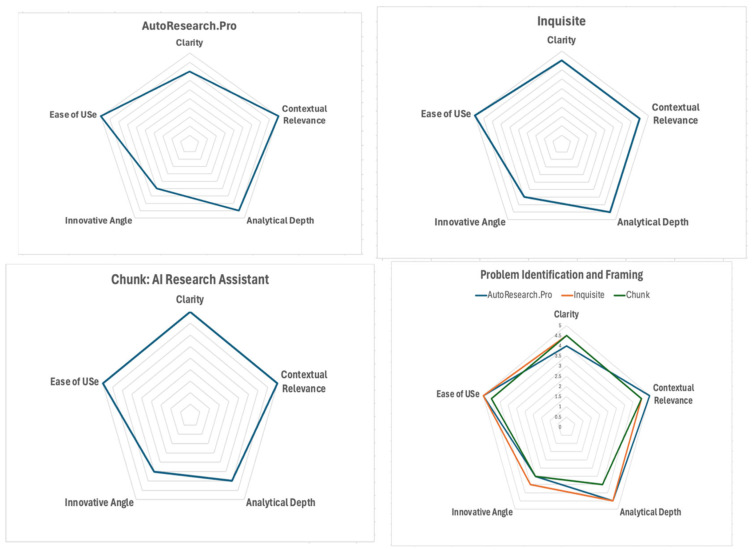
Radar Chart Comparison of 3 GenAI Tools (Autoresearch.pro, Inquisite, Chunk AI) for Problem Identification and Framing step.

The radar charts (Figure 2) represent a comparison of three GenAI tools (Autoresearch.pro, Inquisite, Chunk AI) that are used for the Problem Identification and Framing step across five criteria for evaluation: Clarity, Context Relevance, Analytical Depth, Innovative Angel, and Ease of Use.

- **Autoresearch.pro** performs strongly on Context Relevance and Ease of Use (score of five), suggesting that the tool effectively addresses user needs in the context of SDG Goal 2 and the tool is user-friendly. In addition, Clarity and Analytical Depth are well-rated. This suggests that the tool effectively and clearly communicates insightful information. The Innovative Angle still needs to improve.
- **Inquisite** has the highest rating for Ease of Use, which highlights the user-friendly nature of the tool. Notably, the tool scores high in Contextual Relevance, which aligns with user requirements. Clarity and Analytical Depth reflect good but moderate effectiveness. The lowest score concerns the Innovative Angel.
- **Chunk AI** demonstrates high performance in Clarity, Contextual relevance, and Ease of Use with a near-top rating. This suggests that the tool clearly communicates its output and aligns well with user contexts. However, the rating scores for Analytical Depth and Innovative Angel are lower, which indicates areas for improvement.

**Overall, Autoresearch.pro, Inquisite, and Chunk appear useful** in the Problem Identification and Framing step; among them, Autoresearch.pro stands out for its balance of clarity, depth, and usability. Inquisite leads in ease of use, and Chunk AI excels in delivering clear and contextually aligned insights, which makes each tool valuable depending on specific user priorities.

### 5.2. Use Case 2: Generating Ideas

We demonstrate how GenAI tools helped to generate ideas for Sustainable Development Goal 2 (SDG Goal 2): Zero Hunger. There are 10 Free AIs for generating ideas using the keyword “Brainstorming”, and Table 10 shows the Top 3 GenAIs for Generating Ideas. The full list of 10 GenAIs can be found in Supplementary Material S1 (S1), and a demonstration of the Top 3 GenAIs can be found in Supplementary Material S2 (S2).

**Profiler of GenAI tools for Generating ideas step**

This is a hypothetical example (Table 11) of how we score the top three GenAI tools on the five criteria (Idea Fluency, Novelty (Originality), Relevance, Depth/Elaboration and Ease of Use). The two evaluators independently assessed each tool using a five-point Likert scale. The Spearman–Brown coefficient was 0.80, indicating good agreement between the two judges.

**Figure 3:**
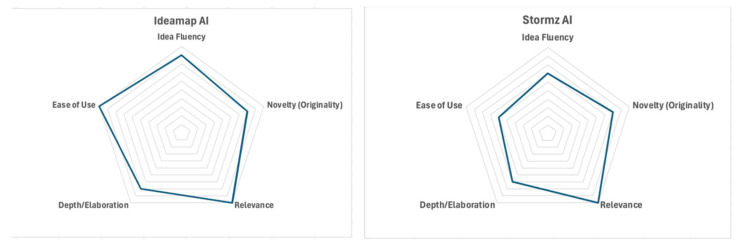

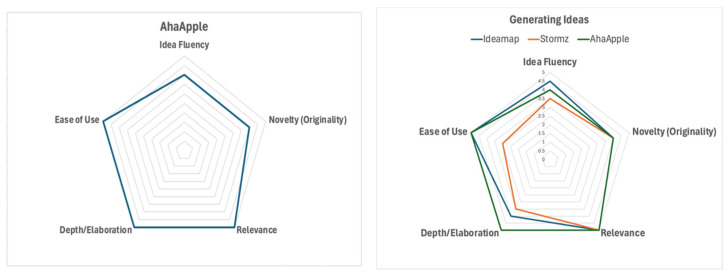
Radar Chart Comparison of 3 GenAI Tools (Ideamap, Stormz, AhaApple) for Generating Ideas step.

The radar charts (Figure 3) represent a comparison of 3 GenAI tools (Ideamap, Stormz, AhaApple) that are used for the Generating ideas step across 5 criteria for evaluation: Idea fluency, Novelty, Relevance, Depth/Elaboration, and Ease of Use.

- **Ideamap** performs strongly on Relevance and Ease of Use, suggesting that the tool generates ideas that are highly aligned with the problem context (in this case, SDG Goal 2) and is user-friendly. In addition, Idea Fluency and Novelty are well-rated. This suggests that Ideamap can produce a good volume of ideas with a fair level of novelty. However, Depth/Elaboration is slightly lower rated. Overall, Ideamap AI stands out as a capable tool for generating relevant and accessible ideas, with room to improve the depth of the creative outputs.
- **Stormz** has the highest Relevance rating, which highlights its alignment with the problem context (SDG Goal 2). Idea fluency and Novelty are rated moderately, which indicates that Stormz can generate a fair number of original ideas but this is not outstanding. In contrast, Depth/Elaboration and Ease of Use are the lowest rated.
- **AhaApple** demonstrates higher performance in all dimensions, particularly Relevance, Depth/Elaboration, and Ease of Use. This suggests that the tool produces highly context-appropriate ideas, develops them in depth, and is user-friendly. Novelty and Idea fluency also score high (4), which suggests that the ideas produced are in good quantity with a reasonable level of creativity.

**Overall, AhaApple** stands out as a comprehensive and well-balanced tool that combines originality/novelty, depth, and usability, making it effective for supporting idea generation.

### 5.3. Use Case 3: Evaluating Ideas

We demonstrate how GenAI tools helped to evaluate ideas for Sustainable Development Goal 2 (SDG Goal 2): Zero Hunger. There are 7 Free AIs for evaluating ideas using the keywords “Idea Evaluation, Idea Testing, and Idea Refinement”, and Table 12 shows the Top 3 GenAIs for Evaluating Ideas. The full list of 7 GenAIs can be found in Supplementary Material S1 (S1), and a demonstration of the Top 3 GenAIs can be found in Supplementary Material S2 (S2).

**Profiler of GenAI tools for Evaluating ideas step**

This is a hypothetical example (Table 13) of how we score the top three GenAI tools under the five criteria (Feasibility, Impact/Value, Risk Identification, Actionability/Next Steps, and Ease of Use). The two researchers (authors) evaluated the performance of the GenAI tools. The Spearman–Brown coefficient was 0.90, indicating strong agreement between evaluators, confirming the consistency of judgments across the five evaluation criteria.

**Figure 4:**
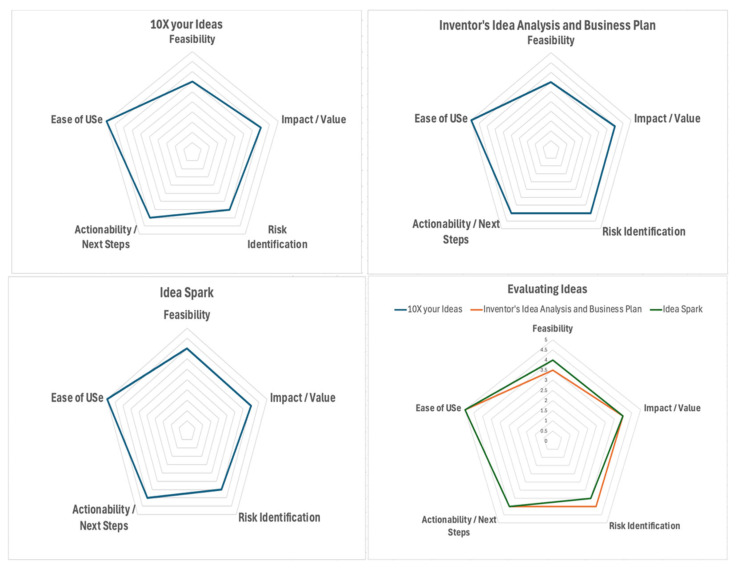
Radar Chart Comparison of 3 GenAI Tools (10X your Ideas, Inventor’s Idea Analysis and Business Plan, Idea Spark) for Evaluating Ideas step.

The radar charts (Figure 4) represent a comparison of three GenAI tools (10X your Ideas, Inventor’s Idea Analysis and Business Plan, Idea Spark) used for the Evaluating ideas step across five evaluation criteria: Feasibility, Impact/Value, Risk Identification, Actionability/Next Steps, and Ease of Use.

- **10X Your Ideas** and **Inventor’s Idea Analysis and Business Plan** show identical scores in all dimensions. It demonstrates steady capabilities in Feasibility (3.5), Impact/Value (4), Risk Identification (3.5 and 4, respectively), Actionability/Next Steps (4), and Ease of Use (5)
- **Idea Spark** slightly outperforms the other two in Feasibility with a score of four.

All three tools receive the highest score of five in Ease of Use. This suggests a strong focus on user-friendliness. Overall, the tools demonstrate similar strengths, particularly in usability and actionable insights. Idea Spark shows marginally higher feasibility and Inventor’s Idea appears slightly stronger in assessing risk. However, the output generated by the tool can be better according to specific prompts or questions (i.e., asking for impact/value or risk identification of SDG Goal 2).

### 5.4. Use Case 4: Deploying and Implementing Idea

We demonstrate how GenAI tools helped to deploy and implement ideas for Sustainable Development Goal 2 (SDG Goal 2): Zero Hunger. There are 4 GenAIs for Deploying and Implementing Ideas using the keywords “prototyping”, and Table 14 shows the Top 3 GenAIs for Deploying and Implementing Ideas. The full list of GenAIs can be found in Supplementary Material S1 (S1), and a demonstration of the Top 3 GenAIs can be found in Supplementary Material S2 (S2).

**Profiler of GenAI tools for Deploying and Implementing idea step**

This is a hypothetical example (Table 15) of how we score the top three GenAI tools under the five criteria (Feasibility, Impact/Value, Risk Identification, Actionability/Next Steps, and Ease of Use). The two researchers (authors) evaluated the performance of GenAI tools. The Spearman–Brown coefficient was 1.0, indicating a perfect agreement between evaluators.

In this step, the two researchers evaluated three GenAI tools based on five criteria. Although the ratings can be given, for some criteria (e.g., Security and Privacy, Reliability and Scalability), the tools Lovable and Prototype App Generator did not generate much relevant for these issues. It appears that the AI tools evaluated, which are the most popular, are not well aligned with what we want AI tools to do in the last step.

## 6. Discussion

### Key Themes Emerging from the Use Cases

**Effective GenAI tools in early-stage creative thinking:** Previous research generally identified GenAI as broadly supportive of creative tasks but did not clearly state which specific stages of the creative process benefit most (Gindert and Müller 2024; Wan et al. 2024). Our findings suggest that publicly available GenAI tools may be particularly useful for structuring early-stage creative thinking, such as during problem identification, idea generation, and idea evaluation. In these phases, GenAI tools such as Inquisite assist by reframing problems from various angles. Ideamap AI assists by rapidly producing diverse and novel ideas. Text-based GenAI tools like 10X your Ideas, Inventor’s Idea Analysis and Business Plan, and Idea Spark (on top of ChatGPT) support preliminary assessment by offering a quick comparison and evaluation framework. However, the effectiveness of existing genAI tools seems less promising in later stages, such as deploying and implementing ideas, where contextual complex, practical constraints, technical integration, and real-world testing require human expertise, deep domain knowledge, and cross-functional collaboration. Thus, this highlights a key strength of current GenAIs in fostering the cognitive and conceptual work at the front end of the creative process rather than executing complex and real-world implementation.

**GenAI-Driven Ideation still needs Human Oversight:** Whereas specific GenAI systems for the Generating Ideas step can generate a quick, wide array of novel ideas, sometimes it pushes creativity into entirely new directions but this is not infallible. Earlier studies (e.g., Urban et al. 2024; Duan et al. 2025) have shown the importance of human oversight. Urban et al. (2024) studied how students co-creating with ChatGPT can improve creative problem-solving performance. They mentioned Hybrid Human-AI Regulation theory (Molenaar 2022), which emphasizes human metacognitive regulation. Similarly, Duan et al. (2025) investigate the issue of narrow creativity in both humans and GenAI through the Circle Exercise. Their work identifies key challenges and opportunities for advancing GenAI-driven creativity support tools. They also argue that humans should oversee, evaluate, and guide GenAI creative process. However, these studies did not investigate precisely how human oversight improves GenAI-generated outcomes. In contrast, our use cases provide a more detailed view by suggesting that human domain expertise is essential for filtering, refining, and contextualizing the AI’s output. This human oversight can prevent biased, impractical, and ethically problematic suggestions from the final outputs/products. Thus, our findings extend and critically engage with the earlier studies by showing that the manner and depth of human oversight significantly impact the effectiveness of Human-AI co-creation.

**AI as a Helper is Efficient But Not Autonomous:** As use cases demonstrate, rapid prototyping, quick information summaries, and automated draft plans all shorten creativity and innovation process cycles. However, no use case suggests that GenAI can be entirely autonomous without human validation, leading to our proposition focused on “AI as a Helper”. The “Human-in-the-loop” (Munro 2021), remains necessary for quality control, ethical governance, and final decision-making. It is worth noting that the use cases examined show that no single genAI system offers the complete set of tools that can facilitate the creative process. Thus, the human operator retains a key role in orchestrating the choice of specific tools and their sequential use in creative thinking.

**The integration with Existing GenAI tools and Processes:** As use cases demonstrate, another theme we found to be important is the ease of integration. Individuals/teams benefit most when GenAI systems are compatible and embedded in collaborative systems. This seamless integration simplifies workflows and maximizes the value AI could provide. The features that facilitate certain AI systems to allow easy integration can be studied and promoted in forthcoming tools.

**Iterative Prompting and Feedback Loops:** Previous studies, e.g., (Duan et al. 2025) discussed iterative prompting techniques in isolation and did not show iteration from the human-AI feedback loop. Our analysis suggests that GenAI tools may be most effective when we use them iteratively. Creative teams often refine and fine-tune prompts and adjust the inputs or parameters to fit their style, depth, or format of outputs. Thus, over multiple iterations, the synergy between “Human expertise” and “Machine intelligence” is expected to yield more sophisticated and context-appropriate solutions. Further research is needed to see the additional value of these recursive loops in the prompting process. For example, to what extent does recursive feedback from humans in the loop lead to actual human-AI synergy, and is the expected improvement in output progressive, or described better as an abrupt boost in performance?

## 7. Implications and Limitations

When considering GenAI adoption within creative workflows, teams or organizations should start by “Identifying clear use cases” aligned with distinct stages of the creative process, such as problem identification, ideation, evaluation, or deployment and implementation. Teams then should roll out the small pilots or proof of concept to refine their processes. Next, selecting appropriate GenAI tools becomes crucial, and it requires a careful comparison of features and evaluation of GenAI tools, which are suited for each creative process (as we proposed the evaluation criteria for GenAI tools with the four common steps of the creative process and give examples on how to score and profiling). Notably, human-in-the-loop protocols should be established where regular checkpoints allow human reviewers to validate AI outputs and provide guidance. Through continuous monitoring and evaluation, organizations and teams can track both quantitative and qualitative metrics by applying lessons learned to update their AI models and refine collaborative processes. Finally, promoting cross-functional collaboration will ensure that AI initiatives align with organizational values or team goals and foster a collaborative culture (e.g., open feedback and shared learning).

When introducing GenAI in the organization, training, and skill development toward collaboration with GenAI are very important. In general terms, it is widely proposed that teams can benefit from practical workshops like prompt engineering, AI-tool customization and data governance that support the new adopters engaging with AI and reducing the overreliance on taking what AI produces as an end product. Ethical usage and responsible innovation should include regular audits to detect and mitigate biases such as GDPR privacy. Establishing collaboration strategies with GenAI that position it as a Helper can contribute to alignment with ethical standards, organizational vision and objectives.

The findings from the current study show specifically how well different AIs perform in the four proposed stages of the creative process. By proposing AI as a supportive yet influential partner, these findings expand existing models of creativity to include the first mode of human-AI collaboration (AI as a Helper), where the outputs require human oversight and expertise. This contributes to the evolving literature on human-AI collaboration dynamics, notably human-led oversight, and AI-driven ideation, which can coexist.

Several limitations remain for future studies to be conducted. First, there is a need for deeper exploration of human-AI collaboration modes; specifically, AI can take more roles as a partner or an autonomous agent (i.e., moving from this study “AI as a Helper” to “AI as a Partner” or even “Creator”). Such inquiries could examine how and when AI can responsibly adopt increased agency without decreasing human ownership. Second, the development of advanced evaluation criteria and metrics is needed. Existing measures of such examples proposed in this study may not fully capture the complexity of AI-assisted creativity, in particular in the required special domains focusing on cultural sensitivity, ethical considerations, or long-term user engagement. Last, longitudinal and comparative research across various domains could illuminate best practices for sustaining ethical and high-impact human-AI collaboration over time. By tackling these limitations, future research can refine theoretical models, inform best practices, and guide policy decisions in the AI-driven creativity era.

## 8. Conclusions

In this study, the proposed framework demonstrates how Generative AI (GenAI) tools can be methodically integrated into each common step of the creative process: (1) Problem Identification and Framing, (2) Generating Ideas, (3) Evaluating Ideas, and (4) Deploying and Implementing Ideas. Our proposition, “AI as a Helper”, leverages AI’s capabilities for data synthesis, novel idea generation, critique, and prototyping. By mapping GenAI tools to specific steps of the creative process and using AI features such as prompt-based text generation, image synthesis, etc., this framework/concept reduces ambiguity and shows when and how AI can add value. This approach ensures that human oversight remains central (human-centered), guiding AI outputs toward contextual awareness, ethical considerations, and high-quality solutions.

The central focus of this study is “AI as a Helper”, which places human expertise and AI agents at the forefront rather than replacing human creativity. AI acts as a supportive helper and ensures that decision-making is in human hands. Thus, it reduces the risks of bias and overreliance on AI. This study’s insights highlight the complementary role of AI in modern creative workflows (creative process), enabling teams to generate, refine, and implement their innovative ideas more efficiently.

## Figures and Tables

**Table 1:** The Creative process stage models and Common part of the process.

Model	Preparation				Ideation						Selection		Implementation Innovation	
1. Wallas (1926)	Preparation				Incubation		Illumination				Verification			
2. Osborn (1953)	Exploring the vision		Formulating challenges		Exploring ideas		Formulating solutions				Exploration/Acceptance		Formulating a plan	
3. Rhodes (1961)	Identifying a Problem or Opportunity				Gathering Ideas						Evaluating, Modifying, and Selecting Ideas			
4. Osborn (1953)	Fact finding				Idea finding						Solution finding			
5. Amabile (1983)	Problem or Opportunity Identification		Preparation		Idea generation						Idea Evaluation			
6. Amabile (1988)	Task presentation		Preparation		Idea generation						Idea Validation	Outcome Assessments		
7. Torrance (1974)	Identifying Problems				Making Guesses		Formulating Hypothesis				Discussing Ideas with Others			
8. Shaw (1989)	Immersion				Incubation		Illumination/Insight				Explain	Creative Synthesis	Validation	
9. Mumford and Reiter-Palmon (1994)	Problem Discover	Problem Definition									Problem Construction			
10. Runco and Dow (1999)	Problem finding				Incubation								Evaluation	
11. Taggar (2002)	Preparation				Synthesis of the team’s ideas								Goal Setting/Strategy to Achieve Team Goal	Participation
12. Nemiro (2002)					Idea generation						Idea development		Idea finalization/closure	Idea Evaluation
13. Reiter-Palmon and Illies (2004)	Problem identification construction		Information Search and Encoding		Idea and Solution Generation						Idea Evaluation and Selection		Implementation Planning and Monitoring	Conclusion
14. Hargadon and Bechky (2006)	Help Seeking		Help Giving		Reflective Reframing						Reinforcement			
15. Zhang and Bartol (2010)	Problem identification		Information Searching and Encoding		Idea and Alternative Generation									
16. Sawyer (2012)	Problem Finding and Formulation		Acquiring Knowledge	Gethering Information	Selecting the best	Externalizing			Combining Ideas		Selecting the best		Externalizing	
17. Botella et al. (2013)					General idea or “vision”						Documentation/Reflection	First sketches; Testing forms or ideas	Provisional object/Draft; Final work/Series	
18. Sadler-Smith (2015)	Preparation				Incubation		Intimation; Illumination				Verification			
19. Cropley (2015)	Preparation		Activation		Generation		Illumination				Verification		Communication	Validation
20. Bonnardel et al. (2018)	Definition and Redefinition (or problem framing) of the creative problem				Openness to aesthetic dimensions, new experiences and new ideas (contributing to divergent thinking)						Reflexive evaluation (contributing to convergent thinking)			
21. Botella-Nicolás and Peiró-Esteve (2018)	Immersion	Reflection	Research	Constraints	Inspiration	Illumination		Assembly		Ideation	Selection	Trials		
											Specification			

**Table 2:** Selection Methodology.

Selection Methodology
Identify the Common Creative Steps Problem Identification and Framing Generating Ideas Evaluating Ideas Deploying and Implementing Ideas Define Relevant Keywords Source GenAI Tools Search the website “There is An AI for that” for Specific Tools Search the website “AI Insider” for Generic Tools (e.g., ChatGPT) Compile a List of GenAI Tools Select the Top 3 Tools Based on Step-Specific and Generic Criteria

**Table 3:** Keywords search for four common parts of the creative process.

1. Problem Identification and Framing	2. Generating Ideas	3. Evaluating Ideas	4. Deploying and Implementing Idea
Research Assistance	Brainstorming	Idea Evaluation Idea Refinement	Prototyping App development

**Table 4:** Proposed criteria for assessing GenAI tools for **Problem Identification and Framing.**

Criterion	Description
**1. Clarity**	How well the AI restates and structures the problem with precision and readability (Reiter and Dale 1997)
**2. Contextual Relevance**	How effectively the AI ties the problem to real-world or organizational contexts (stakeholder needs, domain specifics, business goals) (Manning et al. 2008).
**3. Analytical Depth**	How thoroughly the AI probes underlying causes, sub-problems, and assumptions—laying a solid foundation for deeper exploration (Klein et al. 2006).
**4. Innovative Angle**	How creatively or uniquely the AI reframes the problem, providing fresh, original insights (Colton and Wiggins 2012).
**5. Ease of Use**	How user-friendly and easy to use the tool for the user (Nielsen 1993; Norman 1990).

**Table 5:** Proposed criteria for assessing GenAI tools for **Generating Ideas.**

Criterion	Description
**1. Idea Fluency**	Quantity and variety of ideas produced (Guilford 1967; Torrance 1974).
**2. Novelty (Originality)**	Degree to which ideas are fresh, unique, or unconventional (Runco and Jaeger 2012).
**3. Relevance**	Alignment with the problem statement, domain requirements, or organizational needs (Sternberg and Lubart 1998; Cropley 2006).
**4. Depth/Elaboration**	How well ideas are fleshed out with context, feasibility insights, or next-step thinking (Torrance 1974).
**5. Ease of Use**	How user-friendly and easy to use the tool for the user (Nielsen 1993; Norman 1990).

**Table 6:** Proposed criteria for assessing GenAI tools for **Evaluating Ideas.**

Criterion	Description
**1. Feasibility**	Ability to assess how realistic or implementable an idea is—considering resources, technology, timeline, etc. (Cooper 1990; Tidd and Bessant 2021).
**2. Impact/Value**	Potential positive outcomes (e.g., market potential, social benefit, cost savings) if the idea is implemented (Ulwick 2005).
**3. Risk Identification**	Degree to which the AI pinpoints possible pitfalls or negative consequences, including competitive threats or compliance issues.
**4. Actionability/Next Steps**	Clarity and specificity of how to move forward with an idea, including proposed milestones, stakeholder involvement, or prototyping suggestions (Kotter 1996; Brown 2008).
**5. Ease of Use**	How user-friendly and easy to use the tool for the user (Nielsen 1993; Norman 1990).

**Table 7:** Proposed criteria for assessing GenAI tools for **Deploying and Implementing Idea.**

Criterion	Description
**1. Technical Feasibility**	The tool’s ability to produce technically viable outputs, including code snippets, integration guides, or relevant documentation (Cooper 1990; Pressman 2005).
**2. Integration and Collaboration**	Capacity to integrate with other platforms, APIs, or enterprise systems; also supports multi-user workflows and version control (Crowston and Howison 2005).
**3. Security and Privacy**	Evaluate the measures in place for safeguarding sensitive data, including encryption standards and secure handling of credentials (Anderson 2020).
**4. Reliability and Scalability**	Stability under different loads, clarity on performance or error handling, and ability to scale for larger or more complex projects (Clements et al. 2012).
**5. Ease of Adoption**	How user-friendly and easy to use the tool for the user (Nielsen 1993; Norman 1990).

**Table 8:** Top 3 GenAIs for Problem Identification and Framing based on popularity score.

Top 3 GenAI for Problem Identification and Framing			
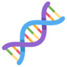	**AutoResearch.** **pro**	4101	https://autoresearch.pro/ (accessed on 25 March 2025)
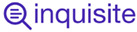	**Inquisite**	2068	https://www.inquisite.ai/ (accessed on 25 March 2025)
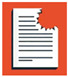	**Chunk: AI Research Assistant**	757	https://free.theresanaiforthat.com/ai/chunk-ai-research-assistant/ (accessed on 25 March 2025)

**Table 9:** Profiler of GenAI tools for Problem Identification and Framing step.

Problem Identification and Framing	AutoResearch Pro	Inquisite	Chunk: AI Research Assistant
**Clarity**	4	4.5	4.5
**Contextual Relevance**	5	4.5	4.5
**Analytical Depth**	4.5	4.5	3.5
**Innovative Angle**	3	3.5	3
**Ease of Use**	5	5	4.5

**Table 10:** Top 3 GenAIs for Generating Ideas based on popularity scores.

Top 3 GenAI for Generating Ideas			
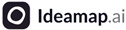	**Ideamap AI**	6376	https://ideamap.ai/ (accessed on 25 March 2025)
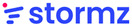	**Stormz AI**	3021	https://stormz.me/ (accessed on 25 March 2025)
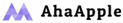	**AhaApple**	1578	https://www.ahaapple.com/idea (accessed on 25 March 2025)

**Table 11:** Profiler of GenAI tools for Generating Ideas step.

Generating Ideas	Ideamap AI	Stormz AI	AhaApple
**Idea Fluency**	4.5	3.5	4
**Novelty (Originality)**	4	4	4
**Relevance**	5	5	5
**Depth/Elaboration**	4	3.5	5
**Ease of Use**	5	3	5

**Table 12:** Top 3 GenAIs for Evaluating Ideas step based on popularity scores.

Top 3 GenAI for Evaluating Ideas			
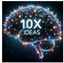	**10X your Ideas**	279	https://chatgpt.com/g/g-629Xxl3wr-10x-your-ideas (accessed on 25 March 2025)
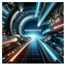	**Inventor’s Idea Analysis and Business Plan**	191	https://chatgpt.com/g/g-oN68t5PO7-inventor-s-idea-analysis-and-business-plan (accessed on 25 March 2025)
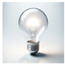	**Idea Spark**	190	https://chatgpt.com/g/g-CYdjsVrEK-idea-spark (accessed on 25 March 2025)

**Table 13:** Profiler of GenAI tools for Evaluating Ideas step.

Evaluating Ideas	10X Your Ideas	Inventor’s Idea Analysis and Business Plan	Idea Spark
**Feasibility**	3.5	3.5	4
**Impact/Value**	4	4	4
**Risk Identification**	3	4	3.5
**Actionability/Next Steps**	3	4	3
**Ease of Use**	5	5	5

**Table 14:** Top 3 GenAIs for Deploying and Implementing Idea step based on popularity scores.

Top 3 GenAI for Deploying and Implementing Ideas			
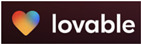	**Lovable**	3794	https://lovable.dev/ (accessed on 25 March 2025)
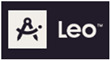	**Leo AI**	627	https://app.getleo.ai/ (accessed on 25 March 2025)
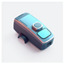	**Prototype App Generator**	138	https://theresanaiforthat.com/@wandame/prototype-generator/ (accessed on 25 March 2025)

**Table 15:** Profiler of GenAI tools for Deploying and Implementing Idea step.

Deploying and Implementing Idea	Lovable	Leo AI	Prototype App Generator
**Technical Feasibility**	5	4.5	3
**Integration and Collaboration**	3	3.5	1
**Security and Privacy**	1	3.5	1
**Reliability and Scalability**	1	3.5	1
**Ease of Adoption**	5	5	5

1 = not offered by the tool.

## Data Availability

The original contributions presented in this study are included in the article/Supplementary Material. Further inquiries can be directed to the corresponding author(s).

## Supplementary Materials

The following supporting information can be downloaded at https://www.mdpi.com/article/10.3390/jintelligence13050057/s1.
