# Supplementary material for: AI as a Helper: Leveraging Generative AI Tools Across Common Parts of the Creative Process
Source: J Intell. 2025 May 20;13(5):57. doi: 10.3390/jintelligence13050057 (PMC12112575; doi:10.3390/jintelligence13050057)
Supplement: Supplementary file 1 [file jintelligence-13-00057-s001.zip › Supplementary material S1 All GenAI tools.pdf]

Supplementary material S1

The supplementary material S1 presents All GenAIs in each step of the creative process.

Use Case 1: Problem Identification and Framing

- **Demonstration of how GenAI tools helped refine or reframe the problem for** Sustainable Development Goal 2 (SDG Goal 2): Zero Hunger

From "There is an AI for that" website Date 25/03/2025 Time 12:00PM
Keywords

- Research Assistance

There are 8 Free GenAIs for Problem Identification and Framing
Keyword: Research Assistance

Table S1.1 8 GenAIs for Problem Identification and Framing

GenAI	Name	Popularity score	Web link	Description
	AutoResearch. pro	4,101	https://autoresearch.pro/	The Research Problem Identifier is a specialized GPT designed to aid in formulating research topics for Masters and PhD studies. Its functionality caters to both knowledgeable professionals and laypersons. Its application spans various disciplines, with specific reference to physics and computer science amongst others, indicating its adaptability across a range of scientific fields.
inquisite	Inquisite	2,068	https://www.inquisite.ai/	Inquisite is the AI-native collaborative science platform that accelerates science R&D teams and streamlines time-intensive workflows by working with AI Assistants. Streamline background research, grant searching and proposal writing so you can get more science done, quicker.
	Chunk: AI Research Assistant	757	https://free.theresanaiforthat.com/ai/chunk-ai-research-assistant/	Chunk: AI Research Assistant is a tool designed to assist users in conducting research with efficiency and precision. Available on a range of Apple devices. With the help of artificial intelligence, Chunk helps to identify, interpret, and summarize relevant data.Using powerful AI algorithms, Chunk effectively screens through large volumes of content, ensuring that users are only presented with the most pertinent information.
Paperguide	Paperguide	595	https://paperguide.ai/	Paperguide is an AI-powered tool designed to streamline the research process for users in academic and professional settings. It functions primarily as a research assistant, reference manager, and writing assistant.
scienceOS	scienceOS	495	https://www.scienceos.ai/	The AI Science Chatbot by scienceOS is an AI-powered research assistant designed to assist researchers in literature exploration. It provides scientific answers, creates tables and diagrams, and draws citation networks, using an extensive database of research papers.
Emergent Mind beta	Emergent Mind	332	https://www.emergentmind.com/	Emergent Mind is an AI Research Assistant primarily designed to assist in computer science inquiries. Its primary function is to provide research-based solutions to questions within the realm of computer science.
Ai	Research bot	237	https://theresanaiforthat.com/@cedbaba/research-bot/	Research bot: Your AI-powered research assistant. Uncover relevant papers, websites, and videos on any topic. Get curated links and recommendations to accelerate your learning and exploration. Dive deep into knowledge effortlessly.
TextLayer	TextLayer	158	https://textlayer.ai/	TextLayer is designed for those working on the front lines of techology. Whether you're a developer, data scientist, researcher, or engineering manager, TextLayer can help you find, understand, and use the latest research papers in your projects.

Use Case 2: Generating Ideas

- **Demonstration of how GenAI tools helped idea generation** for Sustainable Development Goal 2 (SDG Goal 2): Zero Hunger

From "There is an AI for that" website Date 25/03/2025 Time 12:00PM
Keywords
- Brainstorming

There are 10 Free AIs for Generating Ideas.
Keyword: Brainstorming

Table S1.2 10 GenAIs for Generating Ideas

GenAI	Name	Popularity score	Web link	Description
Ideamap.ai	Ideamap AI	6,376	https://ideamap.ai/	Ideamap is an AI-powered tool engineered to facilitate effective and creative brainstorming. Enabling teams to collaborate on ideas, the tool leverages AI to enhance creativity and fast-track the brainstorming process.
stormz	Stormz AI	3,021	https://stormz.me/	Stormz AI Brainstorming tool is designed to help facilitators run brainstorming sessions enhanced with AI features. The tool includes a variety of capabilities to support ideation sessions.
AhaApple	AhaApple	1,578	https://www.ahaapple.com/idea	AhaApple is an AI Idea Generator tool that aims to facilitate the generation of creative and novel ideas. With just one click, users can leverage the power of AI technology and innovative brainstorming techniques to generate a multitude of ideas easily.
Clarify AI Brainstorming	Clarify AI	1,104	https://www.clarify-ai.com/	Clarify AI is a tool designed to help users transform vague ideas into structured insights in a matter of minutes. It aims to assist individuals facing challenges in articulating their thoughts and is tailored for those who navigate ambiguity daily in the workplace.
	Concept Ideas	679	https://free.theresanaiforthat.com/@killithco/concept-ideas/	Concept Ideas: Your creative AI brainstorming partner. Generate five innovative concepts for any project, complete with audience insights, objectives, and standout features. Unleash originality and transform ideas into tangible projects.
	Cove AI	342	https://cove.ai/	Cove is a sophisticated artificial intelligence tool designed to enhance and streamline the conceptualisation and thinking processes. It is mainly aimed at businesses and individuals that want to utilise AI capabilities to improve their ideation strategies.
	Ai Idea Execution Assistant	184	https://free.theresanaiforthat.com/@markwryte/ai-idea-execution-assistant-create-manage-develop/	Ai Idea Execution Assistant transforms abstract concepts into actionable plans. It breaks down ideas, identifies key steps, and provides guidance on resources, challenges, and milestones. Perfect for turning your creative sparks into tangible results.
	Intelligent AI	112	https://free.theresanaiforthat.com/@devashish-3/intelligent-ai/	Intelligent AI: Your advanced brainstorming partner. Conducts real-time research, generates factual insights, and sparks creative ideas. Collaborate dynamically, explore multiple perspectives, and uncover innovative solutions with this powerful AI assistant.
ai	Ai ideas	69	https://free.theresanaiforthat.com/@xneonx/ai-ideas/	Ai ideas: Your creative catalyst. Unleash innovation with tailored, actionable concepts across diverse fields. Get 5+ unique ideas with concise descriptions, blending trends and tech for groundbreaking solutions to your challenges.

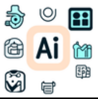	AI Innovative	65	https://free.theresanaiforthat.com/@erahoxha/ai-innovative/	AI Innovative: Your AI-powered innovation consultant. Generate 5+ unique, feasible ideas for products, services, or technologies in any industry. Get brief descriptions, benefits, and target audiences to revolutionize your sector.

Use Case 3: Evaluating Ideas

- **Demonstration of how GenAI tools helped evaluating ideas** for Sustainable Development Goal 2 (SDG Goal 2): Zero Hunger

From "There is an AI for that" website Date 25/03/2025 Time 12:00PM
Keywords
- Idea Evaluation
- Idea Refinement

There are 2 Free AIs for Evaluating Ideas
Keyword: Idea evaluation.

Table S1.3 2 GenAIs for Evaluating Ideas

GenAI	Name	Popularity score	Web link	Description
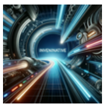	Inventor's Idea Analysis and Business Plan	191	https://chatgpt.com/g/g-oN68t5PO7-inventor-s-idea-analysis-and-business-plan	Inventor's Idea Analysis and Business Plan is a GPT that assists inventors in the crucial process of analyzing their ideas and devising an effective business plan.<br>This application is built on the capabilities of ChatGPT and requires a ChatGPT Plus subscription for access. It serves as a practical tool for potential inventors to clarify and refine their ideas as well as layout a coherent and successful development blueprint for their ventures.
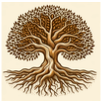	Sage Advisor	74	https://chatgpt.com/g/g-qtL7vElWe-sage-advisor	Sage Advisor is a GPT formulated to score the significance of various projects, ideas or jobs by assessing their meaningfulness. It serves as a tool to assist users in understanding the value and impact their endeavors might have.

There are 5 Free AIs for Evaluating Ideas
Keyword: Idea Refinement.

Table S1.4 5 GenAIs for Evaluating Ideas

GenAI	Name	Popularity score	Web link	Description
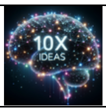	10X your Ideas	279	https://chatgpt.com/g/g-629Xxl3wr-10x-your-ideas	10X your Ideas is a GPT designed to stimulate, refine, and expand ideas using 'First Principles Thinking. Developed by Cuantics Creatives, its main utility is to aid users in enhancing the depth and breadth of their thinking process.
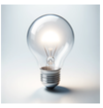	Idea Spark	190	https://chatgpt.com/g/g-CYdjsVrEK-idea-spark	Idea Spark is a GPT that operates on top of ChatGPT. It primarily serves as an innovative facilitator for brainstorming sessions. Designed attentively by Glauser Creative AB, it is deployed with the mission to assist users in coming up with inventive ideas.
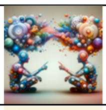	Idea Refiner	127	https://chatgpt.com/g/g-LaqM0nKzt-idea-refiner	Idea Refiner is a GPT designed specifically to aid users in the refinement of their ideas. Its primary objective is to aid in the identification of potential blind spots or unseen implications associated with conceptualization.
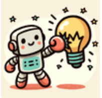	BREAKING IDEAs	119	https://chatgpt.com/g/g-M7V7JmKdP-breaking-ideas	BREAKING IDEAs is a Generative Pre-training Transformer (GPT) developed to assist users in ideation and refining their thoughts. The tool offers two distinct functionalities.

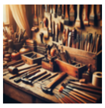	Master Artisan	47	https://chatgpt.com/g/g-TBZCYmlUx-wm-master-artisan	Master Artisan is a GPT designed to guide idea development, with a focus on promoting creativity and practicality. Implemented on top of ChatGPT, it allows users to receive assistance in crafting and refining ideas, with a scope of keeping them creative yet practical.

Use Case 4: Deploying and Implementing Idea

- **Demonstration of how GenAI tools helped** Deploying and Implementing Ideas for Sustainable Development Goal 2 (SDG Goal 2): Zero Hunger

From "There is an AI for that" website Date 21/01/2025 Time 12:00PM
Keywords
- Prototyping
- App development

There are 4 AIs for Deploying and Implementing Ideas
Keyword: Prototyping and App development

Table S1.5 4 GenAIs for Deploying and Implementing Ideas

GenAI	Name	Popularity score	Web link	Description
	Lovable	3,794	https://lovable.dev/	Lovable is an AI-powered platform that enables users of any skill level to create full-stack web applications without requiring coding expertise by simply describing what they want in plain English. Instead of hiring developers, users can generate web apps or websites instantly. - taking you from idea to app as fast as possible.
	Leo AI	627	https://app.getleo.ai/	Leo is presented as the world's pioneering engineering design copilot, that leverages the power of generative AI to enable engineers to convert their ideas into tangible products in a streamlined manner. Leo's AI platform is capable of transforming multiple sources like text, sketches, specifications, and CAD constraints into DFMA-optimized, fully assembled 3D CAD models, thus facilitating a radical shift towards increased efficiency.
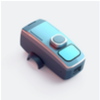	Prototype App Generator	138	https://theresanaiforthat.com/@wandame/prototype-generator/	Prototype Generator: AI-powered design tool that transforms ideas into visual, innovative product prototypes. Crafts detailed, realistic 3D images with multiple angles, balancing functionality and aesthetics for enhanced usability and appeal.
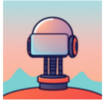	Idea-to-Prototyping AI	110	https://theresanaiforthat.com/@aidammit/idea-to-prototyping-ai/	Idea-to-Prototyping AI: Your digital innovation partner. Transform concepts into actionable project outlines, complete with user personas, sketches, and development roadmaps. Unleash your creativity and bring ideas to life efficiently.

There are Generic AIs for all creative stages

Text-based tools

Table S1.6 Generic AIs for all creative stages (Text-based tools)

	ChatGPT	https://chatgpt.com/	ChatGPT is an AI system that provides conversational interfaces for users. It listens to user input, learns from it and then provides a response. Utilizing OpenAI technology, the system is able to understand and process natural language input, such as speech and text.<br>It can provide an automated response to the user, or it can challenge the user to provide additional information. Using a combination of machine learning algorithms and natural language processing, the system is able to provide meaningful conversations with users.<br>The system is versatile and can be used in a variety of applications, such as customer service, virtual agents, and automated chatbots. By leveraging the power of OpenAI technology, ChatGPT can give users a conversational AI system that is capable of understanding and responding to their needs.
	Gemini	https://gemini.google.com/	Gemini is a high-end Artificial Intelligence model developed by Google. It represents Google's most advanced general AI model to date and is built with multimodal capabilities. These capabilities offer flexibility by supporting varied modes of artificial intelligence, allowing for an interactive, adaptable, and broad-spectrum AI experience.
	Claude Anthropic	https://claude.ai/	Anthropic's Claude is an AI assistant that can be customized to perform a wide range of tasks with a human-like touch. As a Constitutional AI, Claude is designed to be reliable, interpretable, and steerable, with harmlessness training to ensure that it can handle even unpleasant or malicious conversational partners with grace. The tool can be integrated into any product with minimal effort, via standard APIs. It can be trained to handle customer service requests, parse legal documents, act as a virtual sales representative, assist with coaching for personal or career development, and much more.
	Perplexity	https://www.perplexity.ai/	Perplexity is an AI-powered answer engine, designed to provide real-time, accurate and trusted responses to a variety of questions. Its purpose is to serve as a central hub of knowledge and information, available without cost to its users. The AI technology underpinning Perplexity is sophisticated enough to handle a diverse range of queries, covering a broad spectrum of topics, from city infrastructure and current trends, to events and cultural information.
	Meta AI	https://ai.meta.com/	AI Studio, currently available in the US, is a platform where anyone can create a character powered by artificial intelligence. These AI characters are crafted based on the users' interests. This provides a unique opportunity for creators to construct an AI representation of themselves. These AI entities can be connected and used across social media and messaging platforms including Instagram, Messenger, and WhatsApp.
	Microsoft Copilot	https://copilot.microsoft.com/	Microsoft Copilot is an advanced AI assistant developed by Microsoft that is integrated into Microsoft 365 applications to enhance productivity and creativity. Launched in 2023, Copilot leverages powerful language models like GPT-4 and DALL-E 3 to provide a wide range of intelligent features.

Image-based tools

Table S1.7 Generic AIs for all creative stages (Image-based tools)

	Midjourney	https://www.midjourney.com/	Midjourney is an independent research lab dedicated to advancing the imaginative powers of the human species. It is comprised of 11 full-time staff, an incredible set of advisors, legal and financial advisors, and Discord moderators and guides.
	DALL·E 3	https://openai.com/index/dall-e-3/	DALL·E 3, integrated within ChatGPT, advances the frontier of AI-driven image generation by accurately translating textual descriptions into intricate visual artworks. This tool empowers users—ranging from creative professionals to researchers—to effortlessly conceptualize and visualize complex ideas through detailed prompts. Leveraging state-of-the-art AI capabilities, DALL·E 3 enables precise customization, allowing users to refine images to match their vision with minimal effort.
	Bing	https://www.bing.com/images/	The Image Creator tool from Microsoft Bing is an Artificial Intelligence (AI) search feature that generates images based on user input text. The tool uses AI technology to analyze the text provided by the user and automatically creates an image that best matches the given description.
	Leonardo AI	https://app.leonardo.ai/	Leonardo AI is a comprehensive suite of artificial intelligence tools designed to elevate various creative projects through the generation of art, images, and video.
	Stable Diffusion	https://stability.ai/	Stable Diffusion is a versatile platform that leverages generative AI to activate human potential across multiple domains. It provides open models in diverse modalities, making them accessible to all users across different locations.
	Runway	https://runwayml.com/	Runway is an AI-powered content creation suite designed to help users create, edit and collaborate on content. It offers a wide range of AI-powered creative tools, such as text to image generation, erase and replace, AI training, text to color grade, super-slow motion, image to image generation, and infinite image.
