# Supplementary material for: AI as a Helper: Leveraging Generative AI Tools Across Common Parts of the Creative Process
Source: J Intell. 2025 May 20;13(5):57. doi: 10.3390/jintelligence13050057 (PMC12112575; doi:10.3390/jintelligence13050057)

# Supplementary material S2

The supplementary material S2 presents the prompts and results of the top 3 most popular GenAI tools for each step of the creative process

## Use Case 1: Problem Identification and Framing

- **Demonstration of how GenAI tools helped refine or reframe the problem for** Sustainable Development Goal 2 (SDG Goal 2): Zero Hunger

## Problem Identification and Framing

From "There is an AI for that" website Date 26/03/2025 Time 10:30 AM
Keywords
1. Research Assistance

## There are 10 Free GenAIs for Problem Identification and Framing
## Keyword: Research Assistance

**Table S2.1 Most 3 popular tools for Problem Identification and Framing step**

| GenAI | Name | Popularity score | Web link | Description |
|---|---|---|---|---|
| | AutoResearch.pro | 4,101 | https://autoresearch.pro/ | The Research Problem Identifier is a specialized GPT designed to aid in formulating research topics for Masters and PhD studies. Its functionality caters to both knowledgeable professionals and laypersons. Its application spans various disciplines, with specific reference to physics and computer science amongst others, indicating its adaptability across a range of scientific fields. |
| inquisite | Inquisite | 2,068 | https://www.inquisite.ai/ | Inquisite is the AI-native collaborative science platform that accelerates science R&D teams and streamlines time-intensive workflows by working with AI Assistants. Streamline background research, grant searching and proposal writing so you can get more science done, quicker. |
| | Chunk: AI Research Assistant | 757 | https://free.theresanaiforthat.com/ai/chunk-ai-research-assistant/ | Chunk: AI Research Assistant is a tool designed to assist users in conducting research with efficiency and precision. Available on a range of Apple devices. With the help of artificial intelligence, Chunk helps to identify, interpret, and summarize relevant data.Using powerful AI algorithms, Chunk effectively screens through large volumes of content, ensuring that users are only presented with the most pertinent information. |

**Illustrations for Problem Identification and Framing**

Prompt 1: Please identify problem related to SDG Goal 2, Zero Hunger

AutoResearch.pro

Results:

It shows 10 pages presentation about Prompt 1: Please identify problem related to SDG Goal 2, Zero Hunger

## Zero Hunger: Addressing Challenges to SDG Goal 2

A presentation identifying key problems hindering the achievement of SDG Goal 2: Zero Hunger.

**SDG Goal 2: Zero Hunger - A World Without Hunger?**

Exploring the critical challenges in achieving food security and ending hunger by 2030.

### The Dream of Zero Hunger

Identifying Key Problems Impeding Progress Towards SDG Goal 2

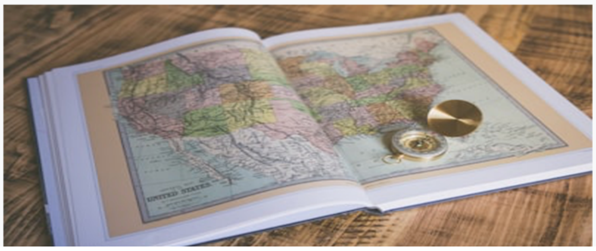

## Climate Change: A Major Threat to Food Production

Erratic weather patterns, droughts, and floods disrupt agriculture and reduce crop yields.

# Climate Crisis, Food Crisis

- - Increased frequency of droughts and floods
- - Rising temperatures impacting crop growth
- - Sea-level rise affecting coastal agriculture
- - Unpredictable rainfall patterns

## Conflict and Instability: Driving Food Insecurity

Wars and civil unrest displace populations, disrupt farming, and hinder humanitarian aid.

# Hunger Amidst Conflict

- - Displacement of farming communities
- - Destruction of agricultural infrastructure
- - Disruption of markets and supply chains
- - Hindrance to humanitarian access

## Poverty and Inequality: Root Causes of Hunger

Limited access to resources, income, and opportunities traps people in cycles of hunger and malnutrition.

# The Poverty-Hunger Trap

- - Lack of purchasing power for food
- - Limited access to land and credit for small farmers
- - Inequality in resource distribution
- - Vulnerability to economic shocks

## Unsustainable Agriculture: Depleting Resources

Practices that degrade land, overuse water, and rely heavily on chemicals threaten long-term food security.

# Farming Our Future Away?

- - Soil degradation and erosion
- - Water scarcity and pollution
- - Over-reliance on chemical fertilizers
and pesticides
- - Deforestation and habitat loss

## Food Waste and Loss: A Significant Inefficiency

Large quantities of food are lost or wasted across the supply chain, from farm to table.

### Wasting Food, Wasting Resources

- - Post-harvest losses in developing countries
- - Food waste in processing and retail
- - Household food waste
- - Inefficient supply chain management

## Lack of Access and Distribution Challenges

Even when food is available, getting it to those in need remains a significant hurdle.

### The Last Mile Problem

- - Inadequate infrastructure in rural areas
- - Supply chain bottlenecks and inefficiencies
- - Market access limitations for smallholder farmers
- - High transportation costs

## Malnutrition in All Forms: A Hidden Crisis

Undernutrition, micronutrient deficiencies, and obesity coexist, impacting health and development.

### Beyond Just Calories: The Malnutrition Challenge

- - Stunting and wasting due to undernutrition
- - Micronutrient deficiencies (vitamin and mineral)
- - Rise of overweight and obesity
- - Impact on physical and cognitive development

## Global Supply Chain Vulnerabilities: Fragility Exposed

Pandemics and geopolitical tensions highlight the risks in relying on complex global food systems.

### Fragile Food Systems

- - Pandemic-related disruptions to trade and transport
- - Geopolitical conflicts impacting food exports
- - Over-dependence on global markets
- - Need for localized and resilient food systems

## Conclusion: Towards a Zero Hunger Future

Addressing these complex challenges requires concerted global action, innovation, and sustainable solutions.

# Moving Forward Together

Addressing the Root Causes of Hunger is Crucial for Achieving SDG Goal 2

- - Sustainable Agriculture and Food Systems
- - Climate Action and Resilience
- - Conflict Resolution and Peacebuilding
- - Poverty Reduction and Social Equity
- - Global Cooperation and Innovation

The interface of AutoResearch.pro

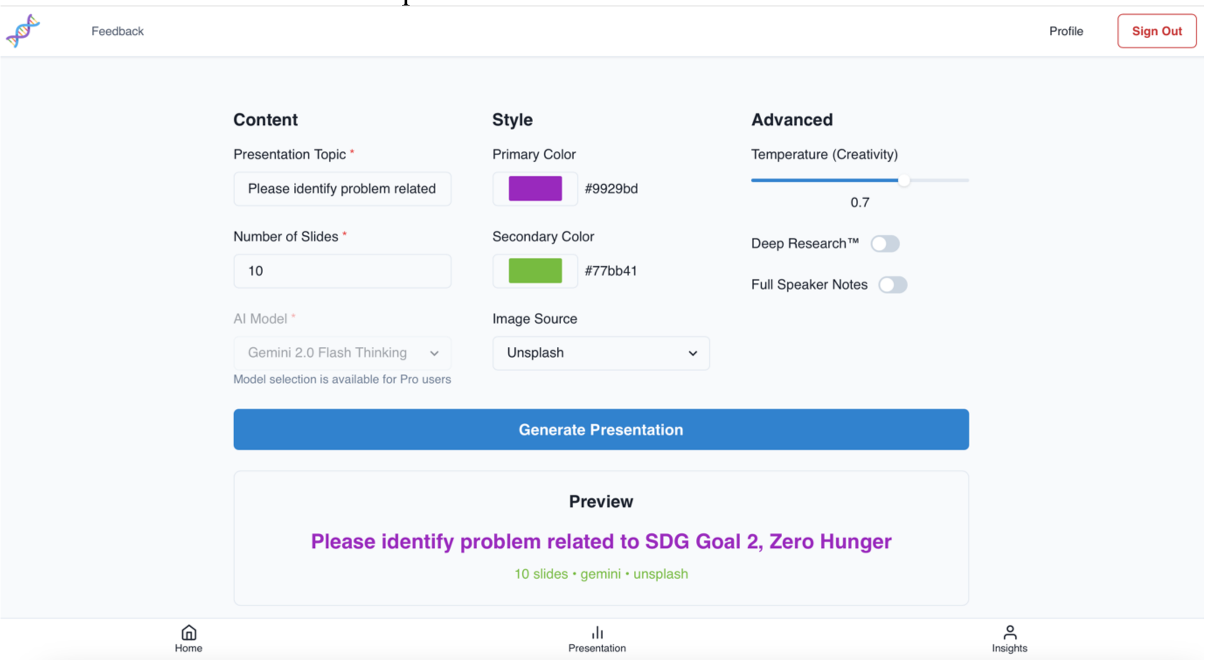

inquisite

Inquisite

Prompt 1: Please identify problem related to SDG Goal 2, Zero Hunger

**Results:**

The UN Sustainable Development Goal (SDG) 2, also known as 'Zero Hunger', aims to end all forms of hunger and malnutrition by 2030 (Atukunda et al. 2021). However, several significant problems are hindering the achievement of this goal.

Many developing countries, especially in Africa, face extreme hunger challenges that are often caused or compounded by bad governance, conflicts, and climate change (Otekunrin et al. 2020). Since 2020, progress towards achieving SDG 2 has faced a sudden stall due to an ongoing "polycrisis," and while some countries are on track, a significant global effort is still necessary to achieve the SDG2 targets (Sporchia et al. 2024). Most African countries have recorded steady reduction in child mortality rates, but they continue to experience high prevalence of undernourishment, stunting, and child wasting, indicating significant challenges hampering the achievement of the zero hunger target (Otekunrin et al. 2020). The COVID-19 pandemic has significantly decelerated progress toward zero hunger by causing an economic downturn that has brutally affected society's ideal living standards and raised social problems such as extreme poverty, famishment, malnutrition, and medical conditions, specifically among vulnerable children (Rahman and Yasin 2022). (Rahman and Yasin 2022). Additionally, food imports are increasingly common in some regions like Pacific Island Countries and Territories (PICTs), providing cheap but not particularly nutritious foods that lead to generalized health problems, with increased reliance on imports itself being an indicator of diminishing food security (Georgeou et al. 2022).

Relevant Sources Sort Export

| Paper | Relevance | Highlights |
|---|---|---|
| Unlocking the potential for achievement of the UN Sustainable Development Goal 2 – 'Zero Hunger' – in Africa: targets, strategies, synergies and challenges Open Access<br>Atukunda et al. 2021<br>*Food & Nutrition Research*<br>66 citations | | • The paper discusses the need for diverse strategies to achieve UN Sustainable Development Goal 2 ('Zero Hunger') by 2030.<br>• It emphasizes overcoming challenges and creating synergies between various SDG targets to end hunger and malnutrition. |
| Assessing the Zero Hunger Target Readiness in Africa in the Face of COVID-19 Pandemic Open Access<br>Otekunrin et al. 2020<br>*Caraka Tani Journal of Sustainable Agriculture*<br>52 citations | | • The paper assesses Africa's readiness to achieve the zero hunger target by 2030, highlighting challenges such as high rates of undernourishment and child wasting despite reductions in child mortality rates.<br>• It recommends that African governments prioritize sustainable agricultural practices and implement policies to combat hunger exacerbated by the COVID-19 pandemic. |
| Food security and small holder farming in Pacific Island countries and territories: A scoping review Open Access<br>Georgeou et al. 2022<br>*PLOS Sustainability and Transformation*<br>20 citations | | • The review identifies the crucial role of smallholder agriculture in supporting food security and economic development in Pacific Island Countries and Territories (PICTs).<br>• Key themes include community adaptation, decreased availability of local foods, dietary changes, and the impact of gender on agricultural production. |
| Zero hunger: future challenges and the way forward towards the achievement of sustainable development goal 2 Open Access<br>Sporchia et al. 2024<br>*Sustainable Earth Reviews*<br>4 citations | | • The paper highlights that progress towards Sustainable Development Goal 2 (Zero Hunger) has stalled due to a global 'polycrisis', necessitating significant global efforts to meet its targets.<br>• Key challenges include the need for a systemic approach and cooperation among various stakeholders to address the complex dimensions of food systems. |
| Children Rights to 'Zero Hunger' and the Execution Challenges during the COVID-19 Crisis<br>Rahman and Yasin 2022<br>*Hasanuddin Law Review*<br>2 citations | | • The paper highlights that the COVID-19 pandemic has significantly hindered progress towards the 'zero hunger' goal, exacerbating issues like extreme poverty and malnutrition among vulnerable children.<br>• It recommends urgent humanitarian relief for food distribution and emphasizes the need for governments to comply with Sustainable Development Goal 2 to protect at-risk children. |
| Achieving Zero Hunger by 2030 A Review of Quantitative Assessments of Synergies and Tradeoffs amongst the UN Sustainable Development Goals<br>Valin et al. 2021<br>7 citations | | • The paper highlights the complex interrelations between Sustainable Development Goal 2 (SDG2) and other goals, emphasizing the need for joint efforts to address SDG2 alongside Goals 1 and 3 for effective outcomes.<br>• It concludes that achieving SDG2 will require significant transformations in food systems, including efficient production, integrated resource management, and changes in consumption patterns, while also addressing trade-offs with other SDGs. |

The interface of inquisite:

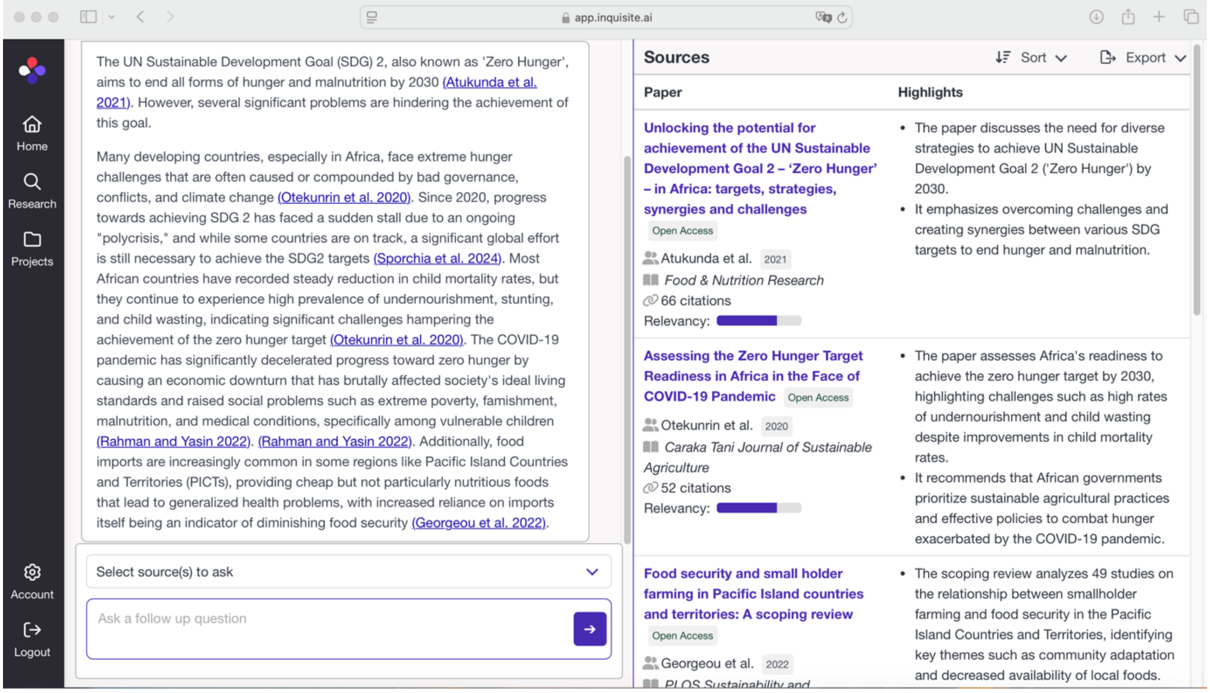


Chunk: AI Research Assistant

Identifying Problems Related to SDG Goal 2: Zero Hunger

Sustainable Development Goal 2 (SDG 2) aims to end hunger, achieve food security and improved nutrition, and promote sustainable agriculture by 2030. However, several challenges hinder progress towards this critical goal. Below are some key issues associated with SDG 2:

1. Food Insecurity
Statistics: According to the World Food Programme, approximately 349 million people worldwide suffer from acute hunger. This reflects a significant increase in food insecurity affecting millions globally.

2. Climate Change Impact
Challenges: Climate change is altering agricultural conditions, leading to reduced crop yields and increased food production costs. This affects food availability and the nutritional quality of food produced.

3. Urbanization
Effects on Food Supply: Rapid urbanization contributes to the formation of food deserts, areas with limited access to affordable and nutritious food, which further exacerbates food insecurity.

4. Economic Factors

- Price Volatility: Global food prices surged due to disruptions in supply chains caused by the COVID-19 pandemic, leading to heightened food insecurity. Rising costs of agricultural inputs and logistics also contribute to market uncertainty.

5. Malnutrition

- Malnutrition Rates: The current annual rate of reduction in stunting, a key indicator of malnutrition, must increase significantly (by 2.2 times) to meet global targets for reducing malnutrition.

6. Sustainable Agricultural Practices

- Need for Innovation: Achieving SDG 2 requires investment in sustainable agricultural practices and innovations to boost productivity while ensuring environmental sustainability.

7. Financial and Market Access

- Barriers: Farmers face barriers to accessing markets, technology, and financial resources necessary for improving agricultural productivity and ensuring food security.

Conclusion

Addressing these issues is critical for meeting SDG 2 and ensuring that all individuals have access to sufficient, safe, and nutritious food year-round. Immediate and intensified efforts are needed across multiple sectors to transform food systems.

Additional Resources and Visuals
To understand these challenges better, here are some informative infographics related to SDG 2:

For further reading and details on specific challenges and pathways towards achieving Zero Hunger, you can refer to the following resources:

- SDG Observatory - University of Texas
- Challenges for Zero Hunger
- Department of Economic and Social Affairs

If you have more specific areas of interest regarding SDG 2 or if you would like to explore other related topics, let me know!
The interface of Chunk:

Interface of Chunk AI:

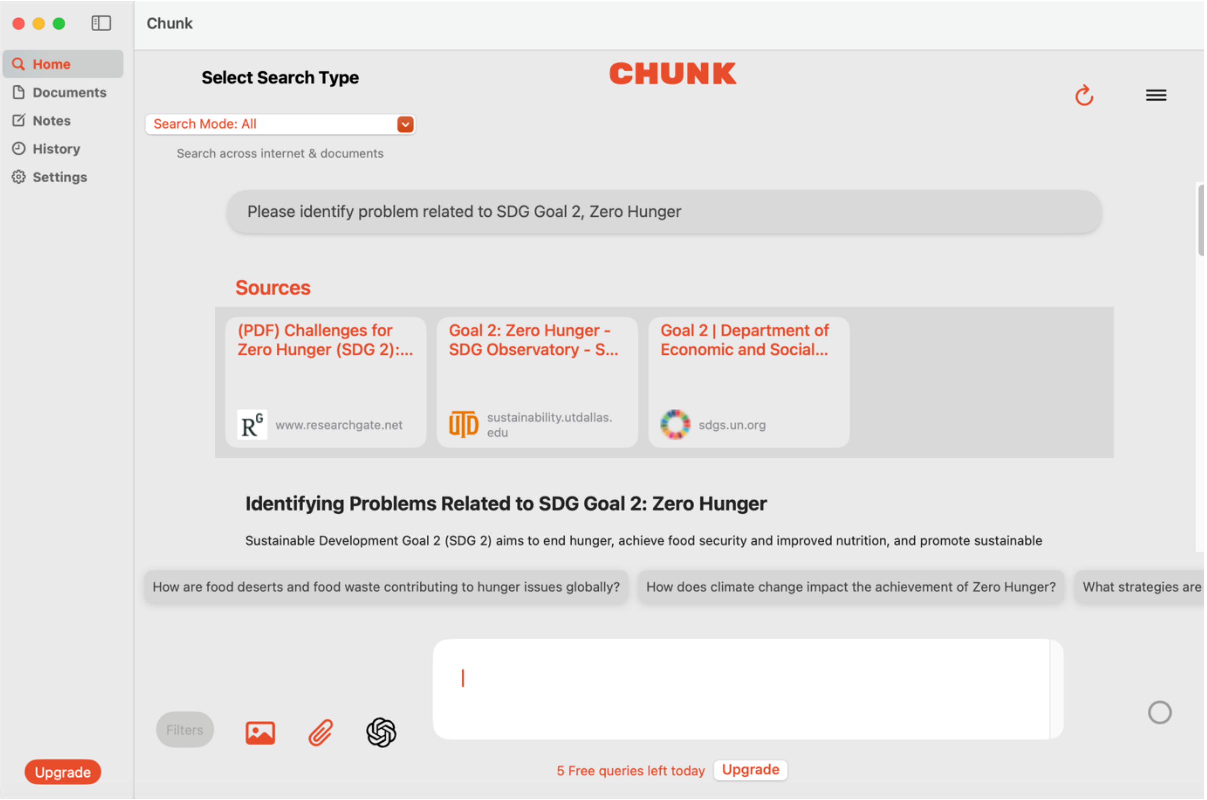


## Use Case 2: Generating Ideas

- **Demonstration of how GenAI tools helped idea generation** for Sustainable Development Goal 2 (SDG Goal 2): Zero Hunger

From "There is an AI for that" website Date 26/03/2025 Time 10:30 AM
Keywords

1. Brainsrorming

## There are 13 Free AIs for Brainstorming
## Keyword: Brainstorming

**Table S2.2 Most 3 popular tools for Generating Ideas step**

| GenAI | Name | Popularity score | Web link | Description |
|---|---|---|---|---|
| Ideamap.ai | Ideamap AI | 6,376 | https://ideamap.ai/ | Ideamap is an AI-powered tool engineered to facilitate effective and creative brainstorming. Enabling teams to collaborate on ideas, the tool leverages AI to enhance creativity and fast-track the brainstorming process. |
| stormz | Stormz AI | 3,021 | https://stormz.me/ | Stormz AI Brainstorming tool is designed to help facilitators run brainstorming sessions enhanced with AI features. The tool includes a variety of capabilities to support ideation sessions. |
| AhaApple | AhaApple | 1,578 | https://www.ahaapple.com/idea | AhaApple is an AI Idea Generator tool that aims to facilitate the generation of creative and novel ideas. With just one click, users can leverage the power of AI technology and innovative brainstorming techniques to generate a multitude of ideas easily. |

Ideamap.ai

Prompt 1: Create many novel and innovative ideas for Solving SDGs Global challenges as Zero Hunger (Your proposed idea should never be applied somewhere before)

The interface of Ideamap:

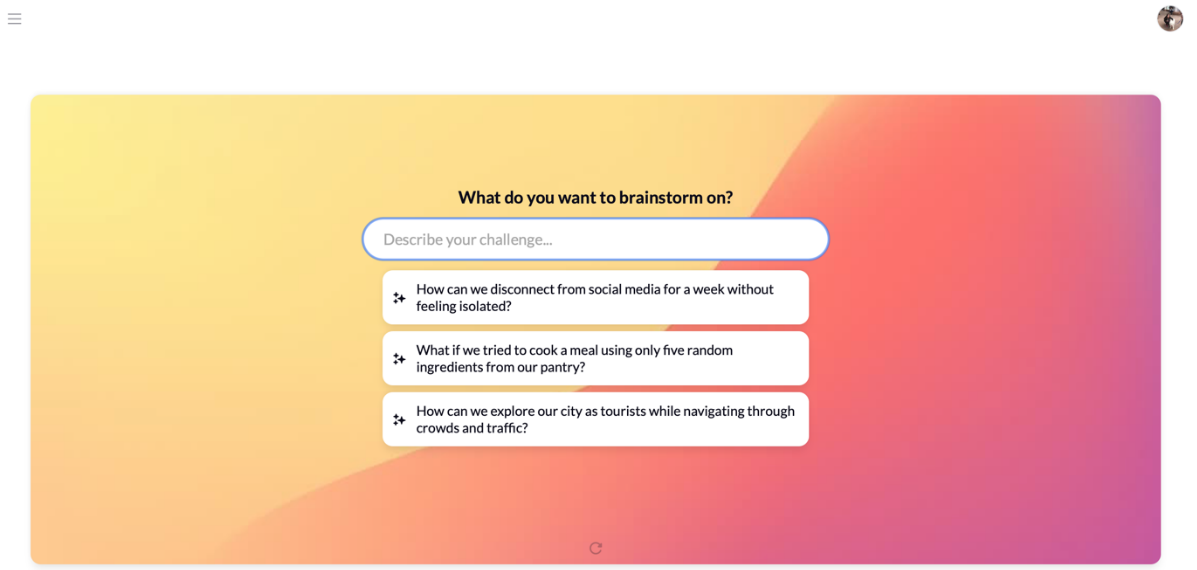


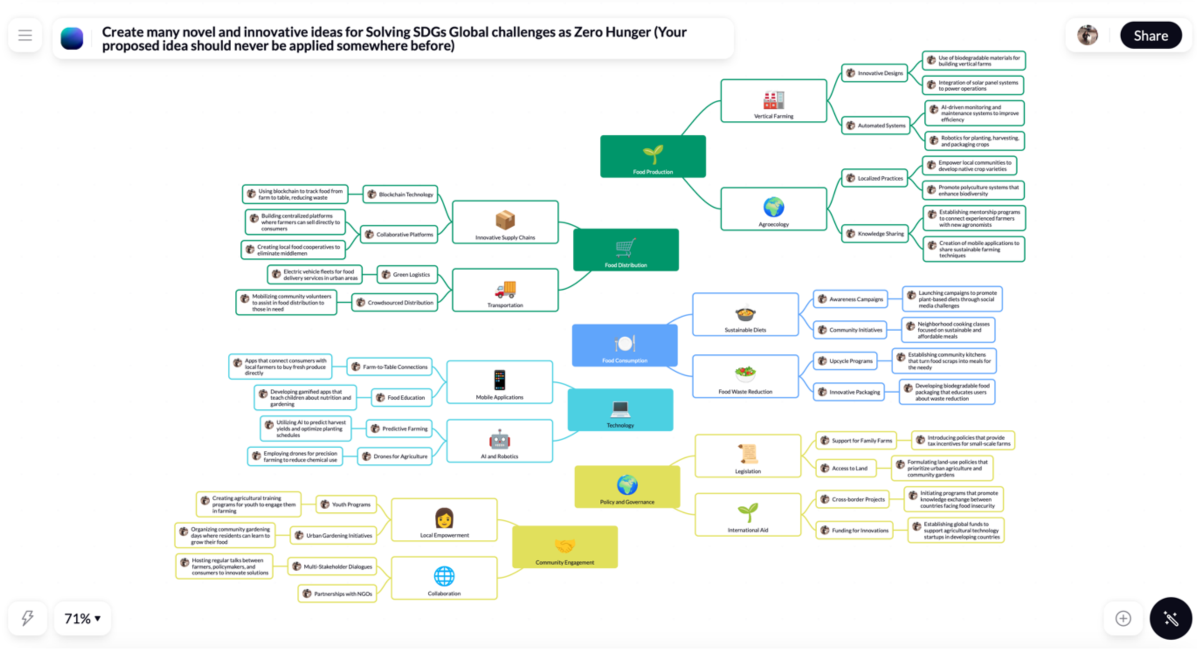

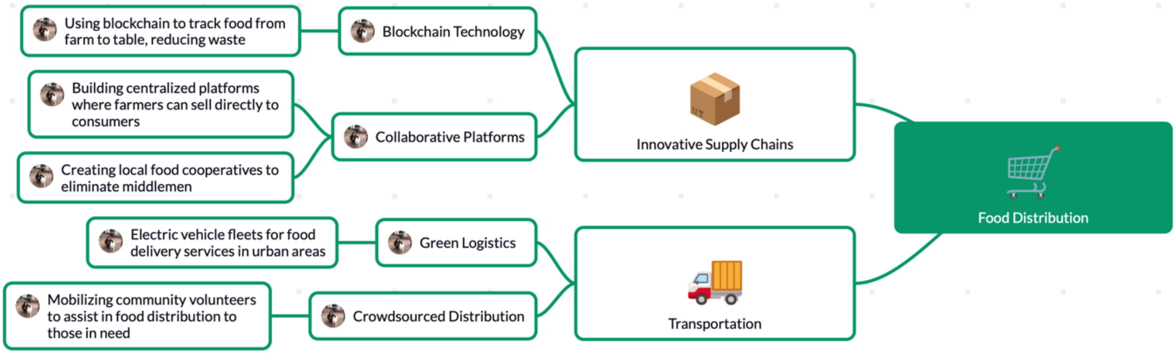
Using blockchain to track food from farm to table, reducing waste
Blockchain Technology
Building centralized platforms where farmers can sell directly to consumers
Creating local food cooperatives to eliminate middlemen
Collaborative Platforms
Innovative Supply Chains
Food Distribution
Electric vehicle fleets for food delivery services in urban areas
Green Logistics
Mobilizing community volunteers to assist in food distribution to those in need
Crowdsourced Distribution
Transportation

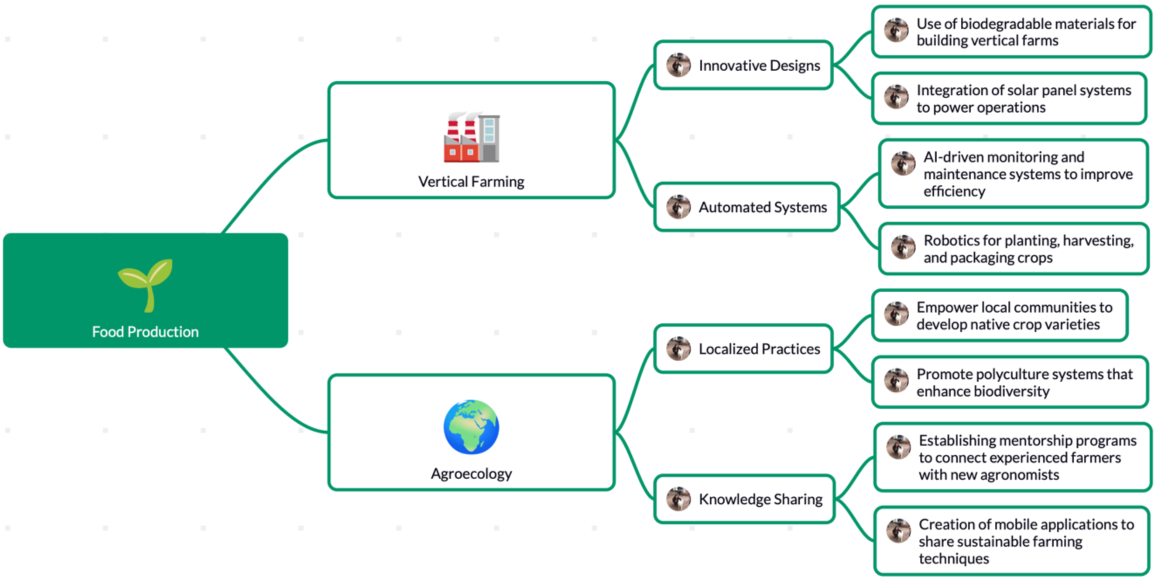
Food Production
Vertical Farming
Innovative Designs
Use of biodegradable materials for building vertical farms
Integration of solar panel systems to power operations
Automated Systems
AI-driven monitoring and maintenance systems to improve efficiency
Robotics for planting, harvesting, and packaging crops
Agroecology
Localized Practices
Empower local communities to develop native crop varieties
Promote polyculture systems that enhance biodiversity
Knowledge Sharing
Establishing mentorship programs to connect experienced farmers with new agronomists
Creation of mobile applications to share sustainable farming techniques

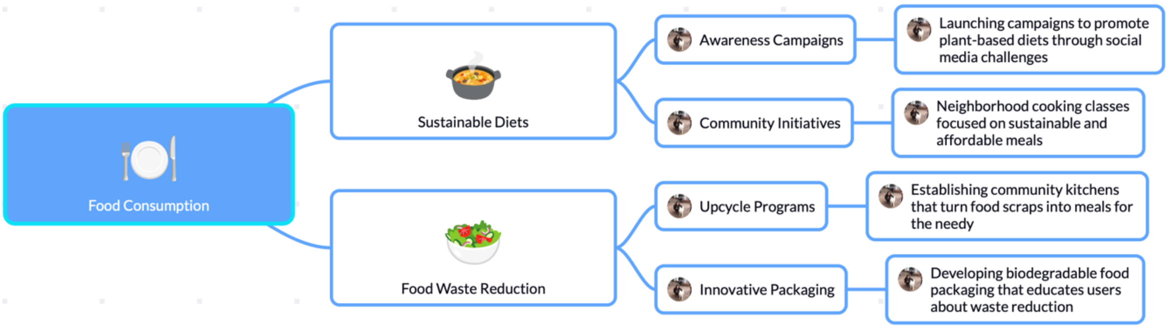
Food Consumption
Sustainable Diets
Awareness Campaigns
Launching campaigns to promote plant-based diets through social media challenges
Community Initiatives
Neighborhood cooking classes focused on sustainable and affordable meals
Food Waste Reduction
Upcycle Programs
Establishing community kitchens that turn food scraps into meals for the needy
Innovative Packaging
Developing biodegradable food packaging that educates users about waste reduction

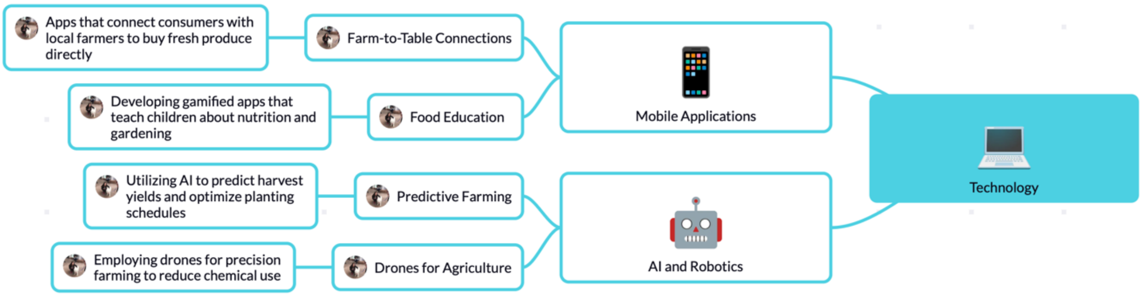
Apps that connect consumers with local farmers to buy fresh produce directly
Farm-to-Table Connections
Developing gamified apps that teach children about nutrition and gardening
Food Education
Mobile Applications
Technology
Utilizing AI to predict harvest yields and optimize planting schedules
Predictive Farming
Employing drones for precision farming to reduce chemical use
Drones for Agriculture
AI and Robotics

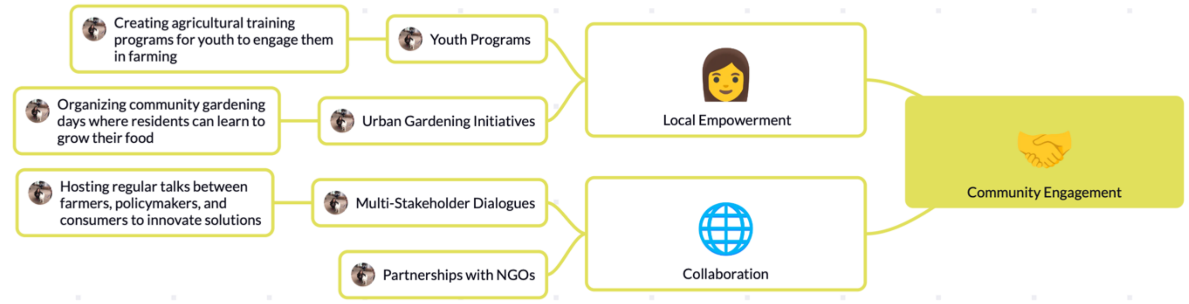


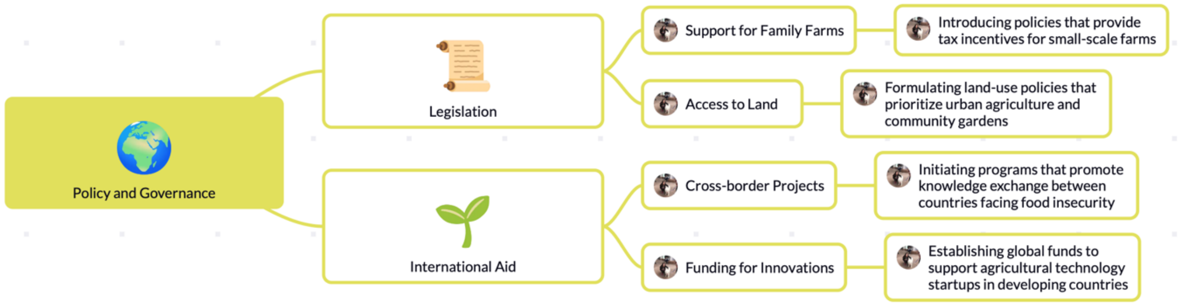


Prompt 2: Select the best novel, innovative idea from the proposed ideas for solving Zero Hunger. Please give scores for each novel idea and why it is the best idea.

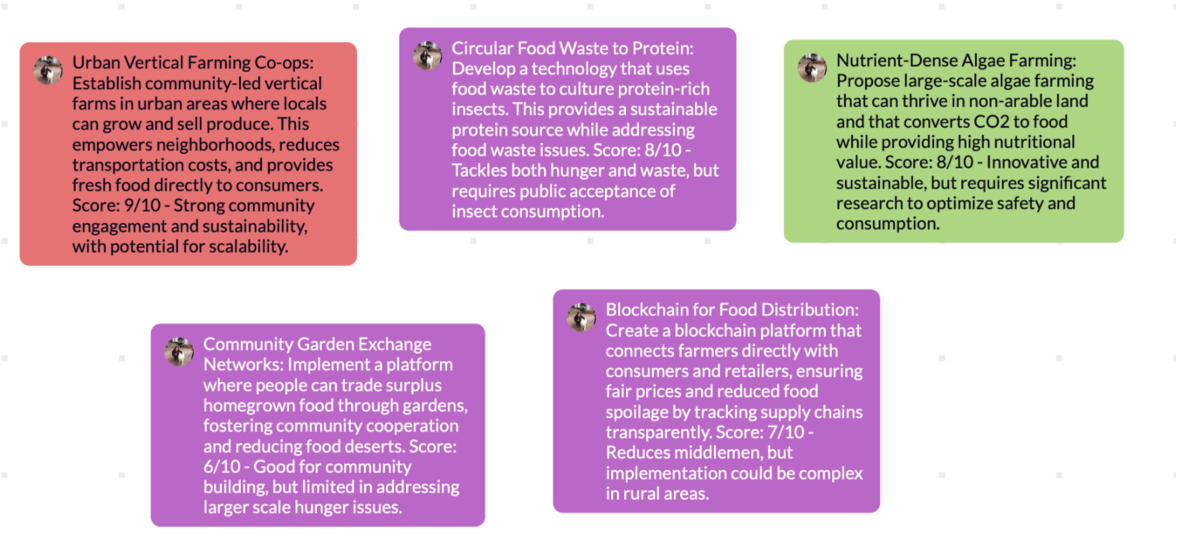

How to use Stormz Can be found in https://www.youtube.com/watch?v=LH5NLC5UFf0

The interface:

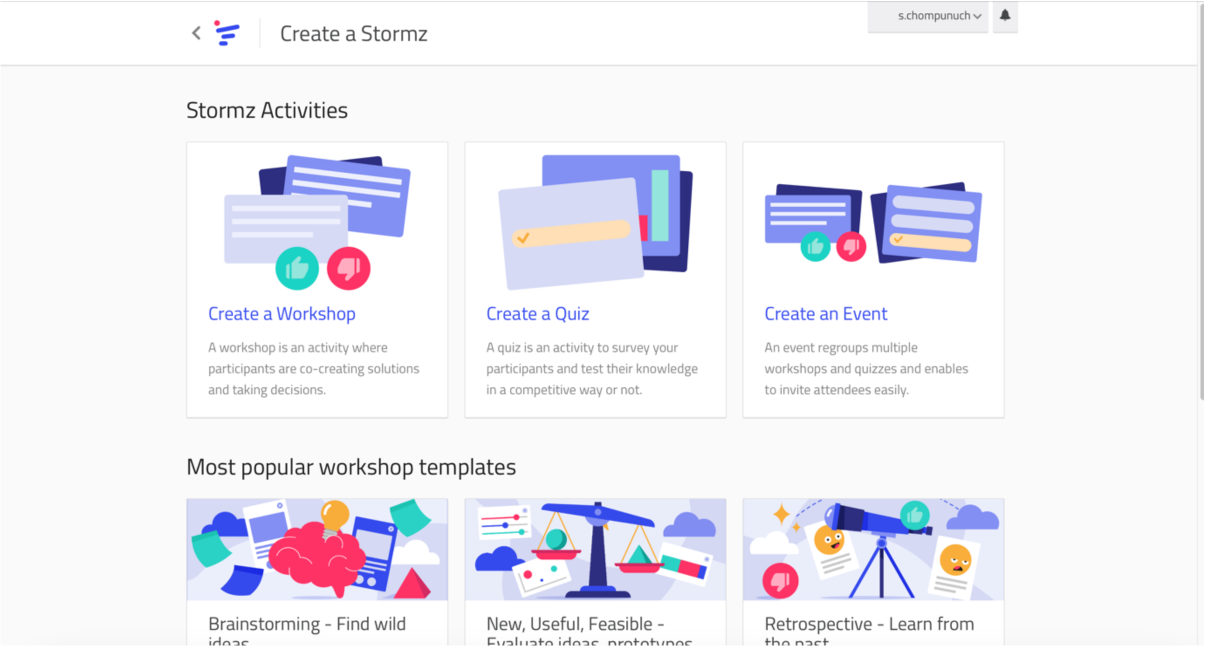


Prompt 1: Create many novel and innovative ideas for Solving SDGs Global challenges as Zero Hunger (Your proposed idea should never be applied somewhere before)

AI generating Ideas:

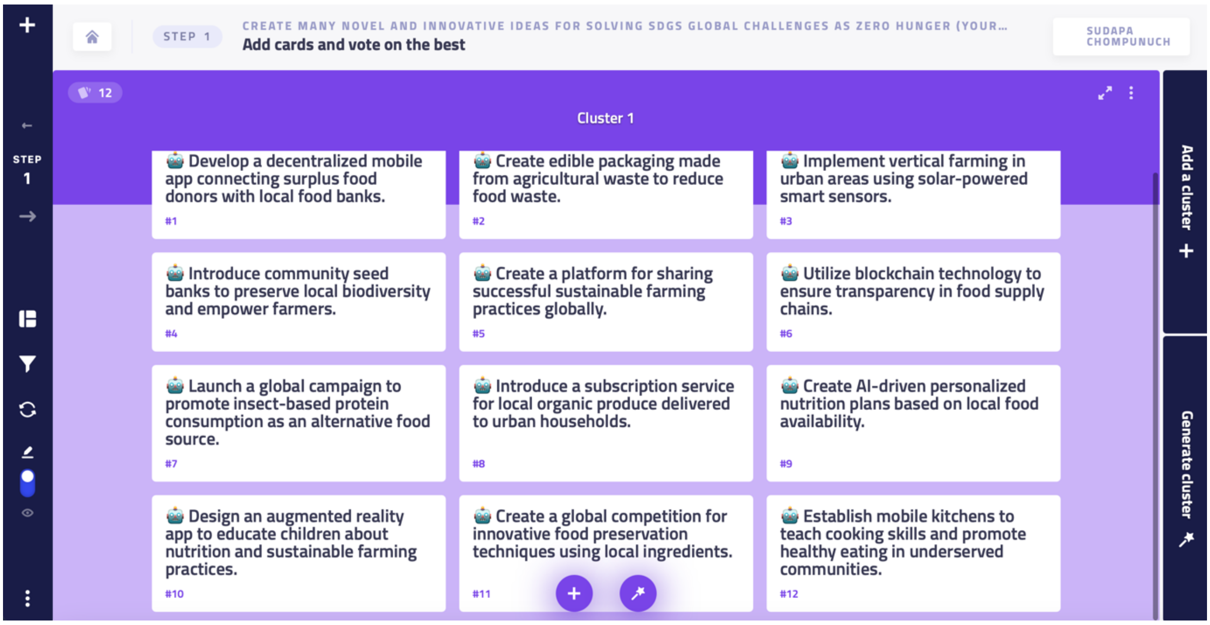

AI Clustering ideas:

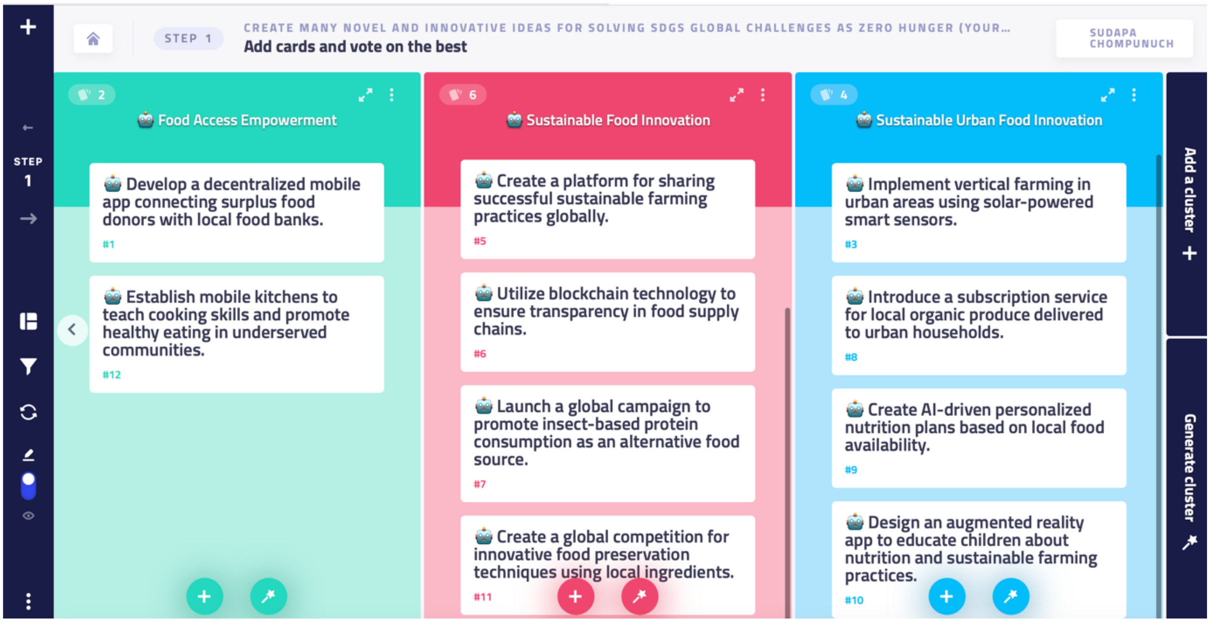


Prompt 2: Select the best novel, innovative idea from the proposed ideas for solving Zero Hunger. Please give scores for each novel idea and why it is the best idea.

**We need human give and rate the best novel idea!**

AhaApple

Prompt 1: Create many novel and innovative ideas for Solving SDGs Global challenges as Zero Hunger (Your proposed idea should never be applied somewhere before)

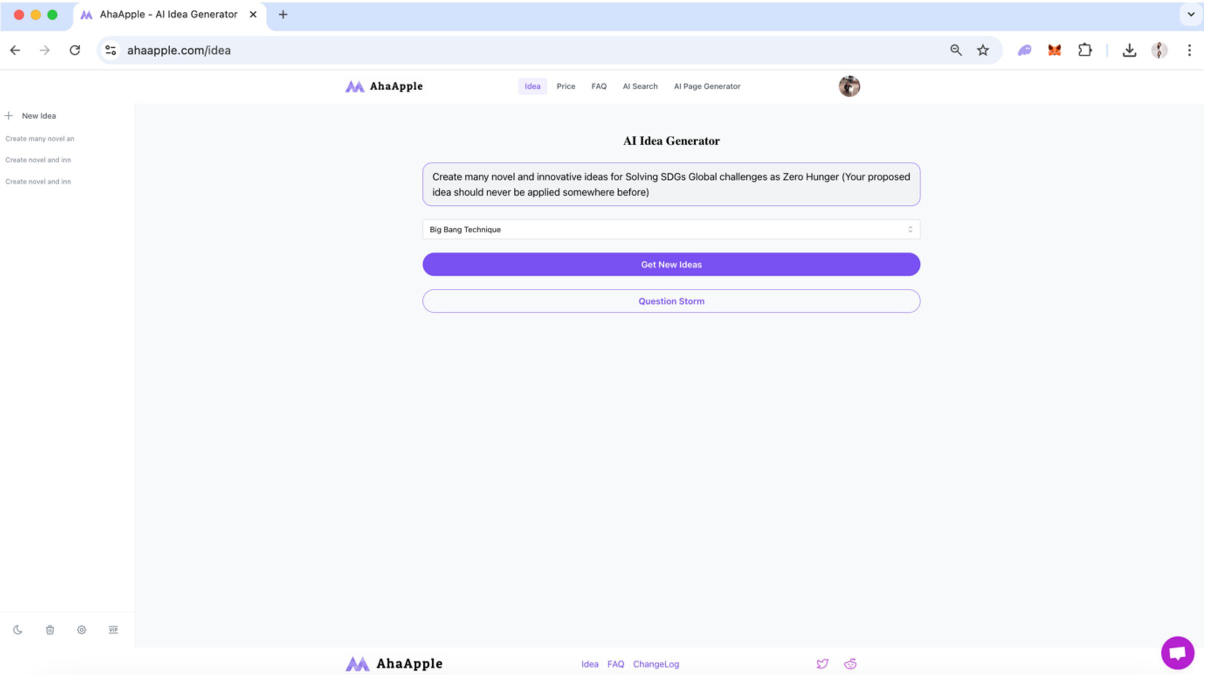


Generate ideas:

1. ecosystem: Develop an AI-driven ecosystem management tool that monitors local biomes and optimizes land use for sustainable farming, enhancing biodiversity while increasing crop yields.
2. agriculture: Create a mobile application that connects urban dwellers with local farmers to facilitate direct food purchases and distribution, reducing reliance on industrial agriculture.
3. food: Design a blockchain-powered food traceability system that helps consumers understand the journey of their food from farm to table, promoting transparency and encouraging support for sustainable practices.
4. community: Establish an augmented reality platform that educates communities about nutrition, cooking techniques, and sustainable farming through interactive experiences on family farms.
5. resources: Launch a global competition to develop new tools or devices that convert food waste into energy, usable fertilizers, or other resources essential for sustainable agriculture.
**6. innovation: Form partnerships with technology companies to create drones equipped with sensors that can detect crop health and automate pesticide application, reducing chemical use while increasing viable crop output.**
7. technologies: Introduce vertical hydroponic farms in urban areas that use advanced technologies to grow food without soil, reducing land use, and maximizing output while improving accessibility to fresh produce.
8. policies: Collaborate with governments to design incentive programs for local organic farmers, providing tax breaks and subsidies to reduce food costs and promote sustainable practices.

9. nutrition: Set up community kitchens that offer cooking classes focused on low-cost, nutritious meals, empowering families with knowledge and skills to improve their diets sustainably.
10. well-being: Create an international initiative that sponsors local farmer's markets, creating certifications for markets committed to selling organic and sustainably produced foods.
11. knowledge: Implement a global network of farmer-to-farmer exchange programs, allowing farmers from different regions to share best practices for sustainable agriculture and pest management.
12. network: Develop a social media platform designed specifically for food producers, allowing them to connect directly with consumers while exchanging knowledge and promoting their products.
13. solutions: Invent a sensor-equipped composting bin that provides users with real-time data on compost quality and guidelines for use, promoting household participation in reducing food waste.
14. diversity: Create a seed bank that preserves native plant seeds and educates farmers about polyculture farming, emphasizing diversified crop production to enhance resilience and nutrition.
15. environment: Launch a campaign promoting edible landscaping in residential areas to convert ornamental plants into nutritious food sources, creating self-sufficient communities.
16. plans: Generate a database that matches vacant urban land with local food cooperatives aiming to convert these spaces into community gardens or urban farms.
17. equity: Start a microloan program specifically for women in rural areas to launch small-scale, sustainable agricultural ventures, thereby increasing female representation in farming.
18. collaboration: Introduce a tool for businesses aimed at facilitating collaboration between chefs and local farmers to create menus based on seasonal availability, boosting local sourcing.
19. security: Develop a mobile game that simulates agricultural challenges, teaching players the importance of sustainable farming practices to ensure global food security in an engaging way.
20. access: Create a smartphone feature that provides users with instant access to recipes based on locally-sourced ingredients that they can find at farmer's markets, maximizing local food utilization.

**It seems like the innovative solution is #6** innovation: Form partnerships with technology companies to create drones equipped with sensors that can detect crop health and automate pesticide application, reducing chemical use while increasing viable crop output

For each idea we can click into “Deep into idea” and “Evalaution”:

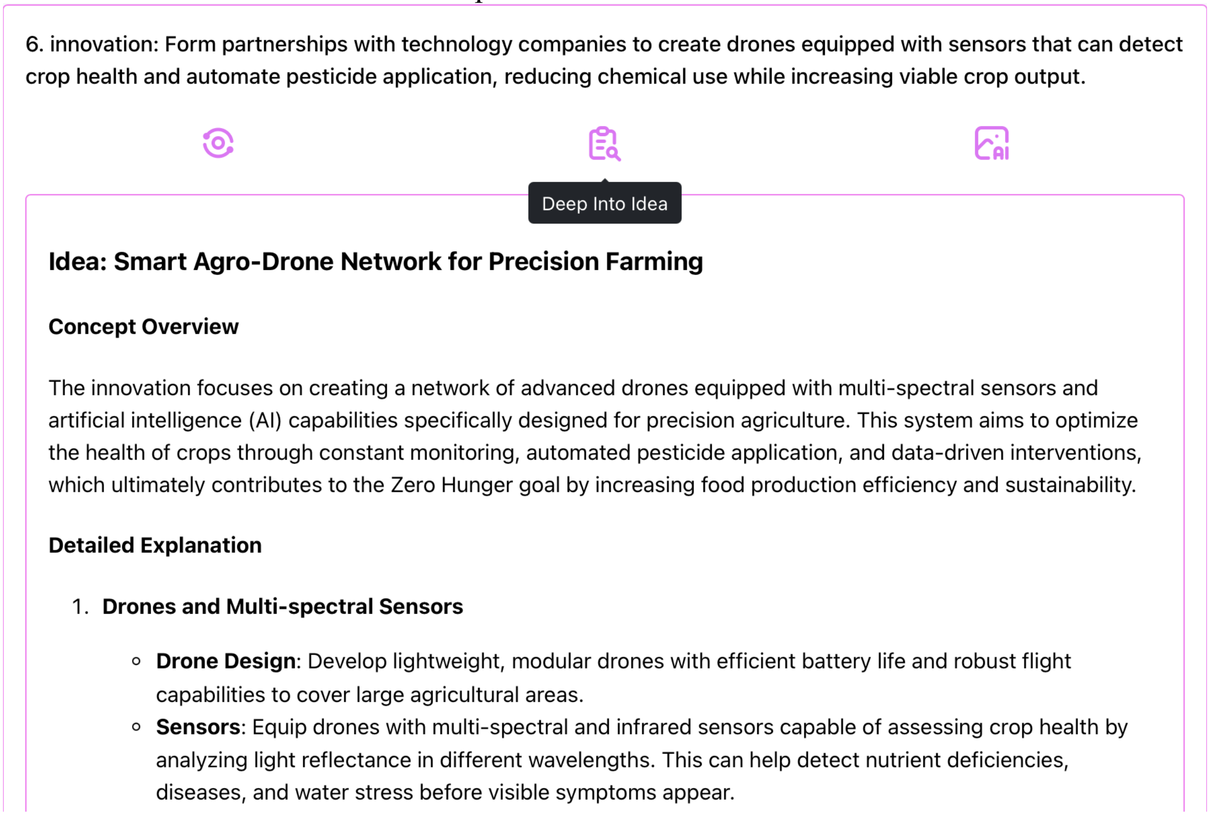


### Idea: Smart Agro-Drone Network for Precision Farming

#### Concept Overview
The innovation focuses on creating a network of advanced drones equipped with multi-spectral sensors and artificial intelligence (AI) capabilities specifically designed for precision agriculture. This system aims to optimize the health of crops through constant monitoring, automated pesticide application, and data-driven interventions, which ultimately contributes to the Zero Hunger goal by increasing food production efficiency and sustainability.

#### Detailed Explanation

1. **Drones and Multi-spectral Sensors**
   - **Drone Design**: Develop lightweight, modular drones with efficient battery life and robust flight capabilities to cover large agricultural areas.
   - **Sensors**: Equip drones with multi-spectral and infrared sensors capable of assessing crop health by analyzing light reflectance in different wavelengths. This can help detect nutrient deficiencies, diseases, and water stress before visible symptoms appear.
   - **AI Integration**: Utilize deep learning algorithms that analyze data collected by the drones, enabling real-time diagnostics of crop health.

2. **Data Algorithms for Crop Management**
   - **Data Capture**: Drones regularly collect data on soil moisture, nutrient levels, pest presence, and overall crop vitality.
   - **AI Processing**: Implement AI-based analytics that process and interpret sensor data, creating actionable insights to inform farmers of specific areas needing attention.

  - **Decision Support System**: Develop a dashboard interface for farmers, offering visual maps and data points that guide the application of water, fertilizers, and pesticides accurately.

3. **Automated Pesticide Application**
   - **Targeted Spraying**: Based on the diagnostic insights, drones can autonomously spray targeted pesticides or organic treatments only in affected areas, minimizing chemical use and maximizing effectiveness.
   - **Eco-Friendly Alternatives**: Explore integrating bio-pesticides and environmentally friendly fungicides into the drone's spraying system to mitigate environmental impacts further.

4. **Partnerships and Community Involvement**
   - **Local Distribution Networks**: Collaborate with local startups and agricultural cooperatives to distribute drone services to farmers at a reasonable cost. This grants smaller and resource-constrained farmers access to advanced technology.
   - **Training Programs**: Implement educational programs for farmers on interpreting data analytics, fostering a more profound understanding of their fields and promoting sustainable agricultural practices.

5. **Environmental Impact Assessment**
   - **Sustainable Practices**: Monitor and reduce the overall chemical footprint in farming through automated precision applications.
   - **Biodiversity Preservation**: Use non-invasive sensors and AI technology to identify areas of high biodiversity, creating micro-reserves within farming areas to promote ecosystem health.

6. **Implementation Phases**
   - **Pilot Program**: Initiate a pilot program with select farmers to test the drone system in a controlled environment, gather feedback, and make necessary adjustments.
   - **Scalability**: Based on pilot program success, scale the network by incorporating more drones and expanding to a broader range of crops and regions.
   - **Cloud Infrastructure**: Establish a centralized cloud infrastructure for the data collected, allowing for over-the-air software updates and broader accessibility to analytical tools for all participating farmers.

7. **Funding and Sustainability**
   - **Grants and Subsidies**: Seek funding from agricultural innovation grants offered by governments and NGOs focused on sustainable food production and technology integration.
   - **Subscription Model**: Consider a subscription-based model where farmers pay a fee for drone services that provide continuous data monitoring and support, thus ensuring the project's long-term sustainability.

### Visual Representation
A conceptual diagram showing:
- Drones flying over various fields equipped with sensors.
- Screens showing the data analytics dashboard, including health status maps of crops.
- Visuals of drone applications targeting specific crop areas, highlighting precise pesticide application.

### Conclusion

This innovative solution leverages cutting-edge drone technology and AI to create a more sustainable, efficient approach to farming that caters to both the health of the crops and the environment. By enabling precise, data-driven farming methods, it directly addresses the Zero Hunger SDG by potentially increasing crop yields, reducing food waste, and promoting environmentally friendly agricultural practices.

Evaluation

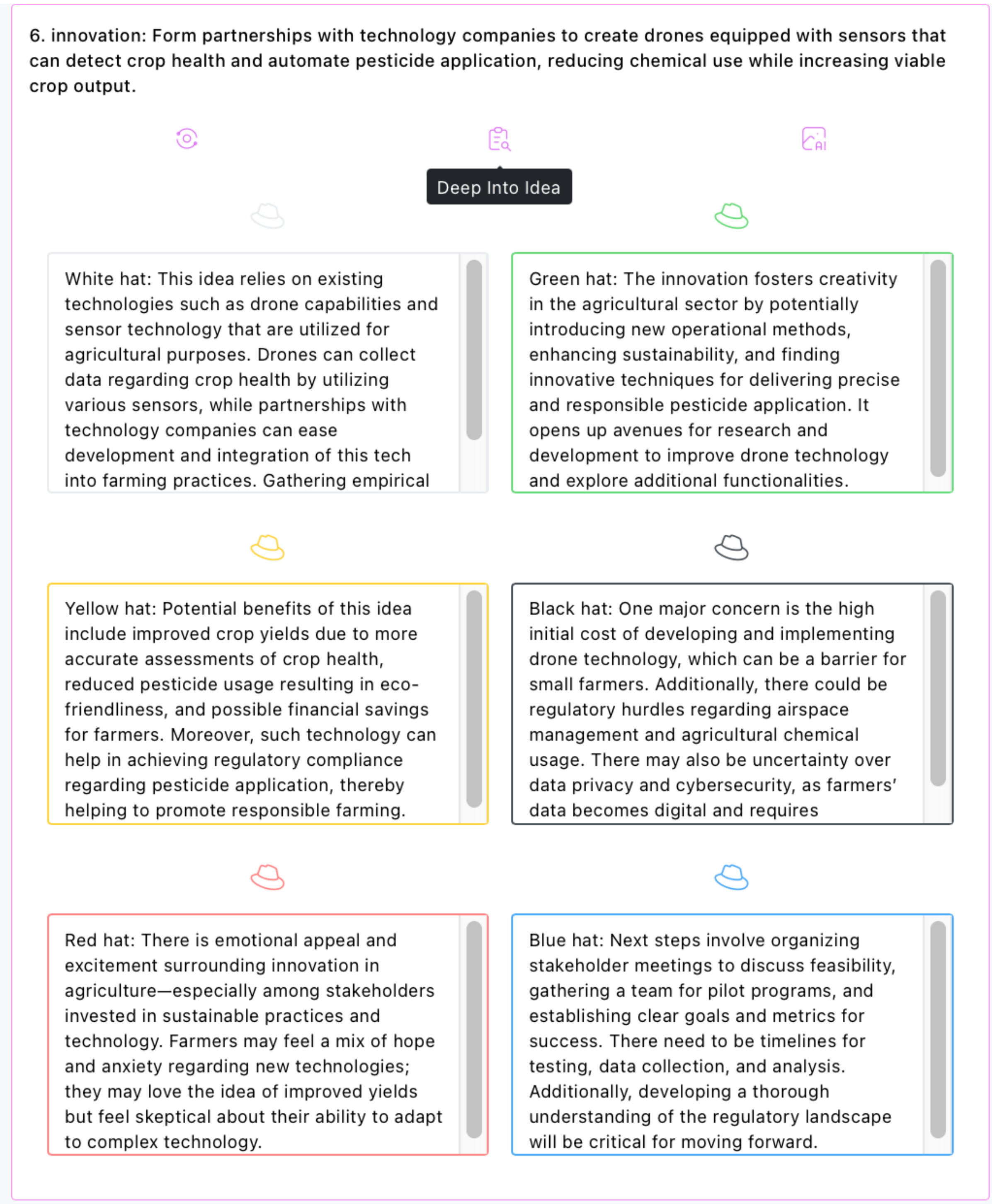

## Use Case 3: Evaluating Ideas

- **Demonstration of how GenAI tools helped evaluating ideas** for Sustainable Development Goal 2 (SDG Goal 2): Zero Hunger

## Evaluating Ideas

From "There is an AI for that" website Date 26/03/2025 Time 10:30AM
Keywords
1. Idea Evaluation
2. Idea Refinement

## Illustrations for Evaluating Ideas

**Table S2.3 Most 3 popular tools for Evaluating ideas step**

| GenAI | Name | Popularity score | Web link | Description |
|---|---|---|---|---|
| 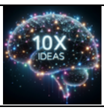 | 10X your Ideas | 279 | https://chatgpt.com/g/g-629Xxl3wr-10x-your-ideas | 10X your Ideas is a GPT designed to stimulate, refine, and expand ideas using 'First Principles Thinking. Developed by Cuantics Creatives, its main utility is to aid users in enhancing the depth and breadth of their thinking process. |
| 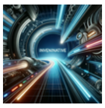 | Inventor's Idea Analysis and Business Plan | 191 | https://chatgpt.com/g/g-oN68t5PO7-inventor-s-idea-analysis-and-business-plan | Inventor's Idea Analysis and Business Plan is a GPT that assists inventors in the crucial process of analyzing their ideas and devising an effective business plan. This application is built on the capabilities of ChatGPT and requires a ChatGPT Plus subscription for access. It serves as a practical tool for potential inventors to clarify and refine their ideas as well as layout a coherent and successful development blueprint for their ventures. |
| 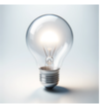 | Idea Spark | 190 | https://chatgpt.com/g/g-CYdjsVrEK-idea-spark | Idea Spark is a GPT that operates on top of ChatGPT. It primarily serves as an innovative facilitator for brainstorming sessions. Designed attentively by Glauser Creative AB, it is deployed with the mission to assist users in coming up with inventive ideas. |

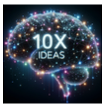

10X your Ideas

Prompt 1:

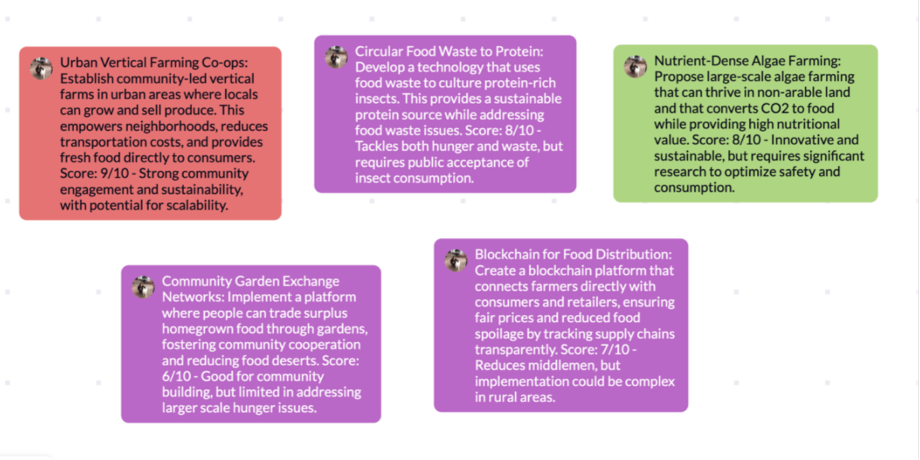


Select the best novel, innovative idea from the proposed ideas for solving Zero Hunger. Please give scores for each novel idea and why it is the best idea.

The interface same as ChatGPT:

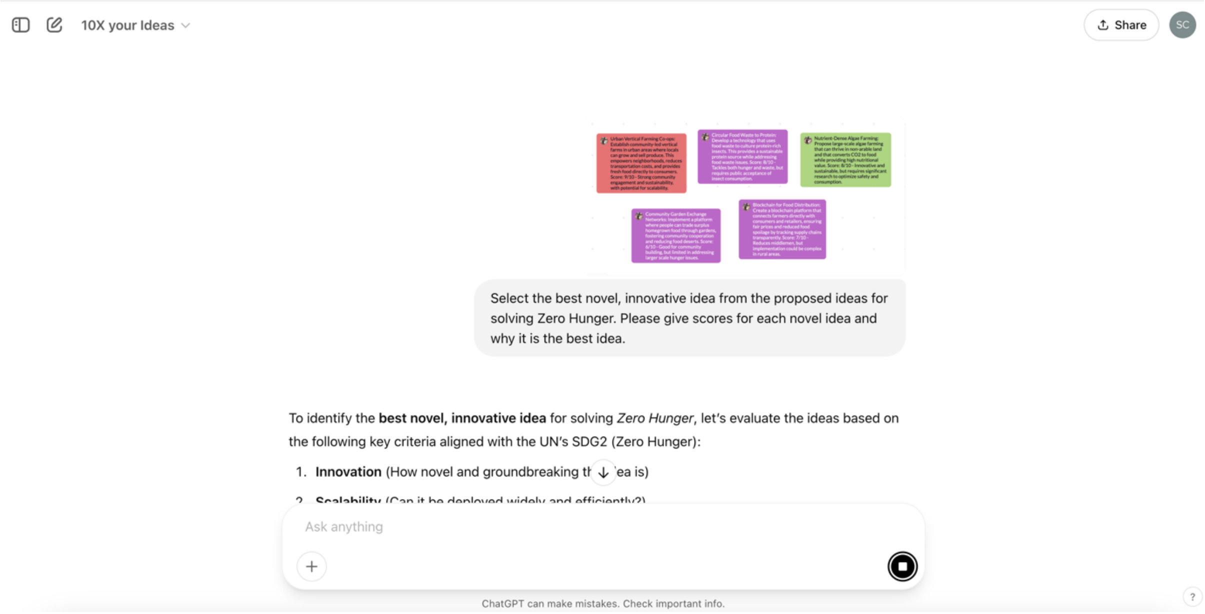


Result:

To identify the **best novel, innovative idea** for solving *Zero Hunger*, let's evaluate the ideas based on the following key criteria aligned with the UN's SDG2 (Zero Hunger):

1. **Innovation** (How novel and groundbreaking the idea is)
2. **Scalability** (Can it be deployed widely and efficiently?)
3. **Sustainability** (Environmental, social, and economic impact)
4. **Feasibility** (How practical is the implementation?)
5. **Impact on Hunger** (Directly addresses food insecurity or hunger?)

---

## 🔴 Urban Vertical Farming Co-ops

- **Innovation**: 7/10 – Not new, but applying a cooperative model adds uniqueness.
- **Scalability**: 8/10 – High in cities with space adaptation.
- **Sustainability**: 9/10 – Local, fresh food with lower emissions.
- **Feasibility**: 8/10 – Technically feasible but may face zoning or funding hurdles.
- **Impact on Hunger**: 8/10 – Reduces food deserts and empowers communities.
- ⭐ **Final Score**: **8/10**
  📝 *Strong, proven approach with social innovation—but not the most novel.*

---

## 🟣 Circular Food Waste to Protein (Insect Protein Tech)

- **Innovation**: 10/10 – Highly novel use of circular economy principles.

- **Scalability**: 9/10 – Tech-based, scalable with infrastructure.
- **Sustainability**: 10/10 – Solves both food waste and protein scarcity.
- **Feasibility**: 6/10 – Major barrier: consumer adoption of insect-based foods.
- **Impact on Hunger**: 9/10 – High nutritional impact and efficient protein source.
- ⭐ **Final Score**: **8.8/10**
  📝 *Leverages biotech and circular economy to create low-input, high-yield protein.*

---

## 🟢 Nutrient-Dense Algae Farming

- **Innovation**: 9/10 – Novel use of non-arable land and carbon.
- **Scalability**: 7/10 – Technically possible, but infrastructure-heavy.
- **Sustainability**: 10/10 – Converts $CO_2$ into food, very climate-friendly.
- **Feasibility**: 5/10 – Requires deep R&D and consumer trust.
- **Impact on Hunger**: 7/10 – High potential, but indirect today.
- ⭐ **Final Score**: **7.6/10**
  📝 *Promising future tech, but more theoretical today.*

---

## 🟣 Blockchain for Food Distribution

- **Innovation**: 8/10 – Innovative use of blockchain for transparency.
- **Scalability**: 7/10 – Tech-scalable but infrastructure-intensive.
- **Sustainability**: 6/10 – Tech helps reduce waste, but energy use can be high.
- **Feasibility**: 6/10 – Rural and low-tech areas may struggle.
- **Impact on Hunger**: 6/10 – Indirect; supports logistics, not production.
- ⭐ **Final Score**: **6.6/10**
  📝 *Useful enabler, but not directly attacking the core of hunger.*

---

## 🟣 Community Garden Exchange Networks

- **Innovation**: 6/10 – Modest innovation in a known model.
- **Scalability**: 6/10 – Limited to areas with gardening capacity.
- **Sustainability**: 8/10 – Encourages reuse and local food culture.
- **Feasibility**: 9/10 – Highly doable with minimal tech.
- **Impact on Hunger**: 5/10 – Good locally, not impactful at scale.
- ⭐ **Final Score**: **6.8/10**
  📝 *Valuable for community-building, but not a game-changer for global hunger.*

---

## 🏆 Best Novel Idea: Circular Food Waste to Protein (Insect Farming Tech)

**Final Justification**:
It's the **most innovative** and addresses **two problems at once**: food waste and protein scarcity. It's **sustainable, scalable**, and has the **potential to transform global food systems**, especially in regions with limited arable land or water resources. The **main challenge is cultural acceptance**, which can be overcome with education and product innovation (e.g., insect protein powders, bars).

Would you like help mapping out how this idea could be brought to market or scaled across different regions?

Prompt 2:

Best Idea for SDG Goal 2, Zero Hunger: Circular Food Waste to Protein (Insect Farming Tech)

1. What is the concept of your project? (e.g., Circular Food Waste to Protein: Insect Farming Tech)

2. Who is your target audience?

3. What are the core features of your project? (e.g., digital platforms, mobile apps, online communities)

Result:

Absolutely, let's build this out into a sharp, strategic concept aligned with **SDG Goal 2: Zero Hunger**, using a first-principles approach.

---

## ✅ 1. What is the concept of your project?

**Circular Food Waste to Protein: Insect Farming Tech**

This project converts **organic food waste** into **nutrient-rich insect protein** using **bioconversion technology**. The insects (e.g., black soldier fly larvae) are farmed in controlled environments and processed into **high-protein animal feed** or **human-grade protein products**, creating a **circular, sustainable, and scalable food system** that addresses both **malnutrition** and **food waste**.

---

## ✅ 2. Who is your target audience?

We segment the audience by both *end users* and *enablers*:

🎯 **Primary Target Audiences:**

- **Undernourished populations** in food-insecure regions (via NGOs or government distribution)
- **Farmers & fishery operators** needing low-cost, sustainable animal feed
- **Low-income urban communities** lacking access to affordable protein

🔗 **Secondary Stakeholders:**

- **Municipal waste management bodies** (as waste suppliers)
- **Governments & development agencies** (as policy/investment partners)
- **Sustainable food startups and social enterprises**
- **Investors in agritech / circular economy ventures**

---

## ✅ 3. What are the core features of your project?

Let's break this into three layers: **Tech Infrastructure**, **Distribution**, and **Community Engagement**

---

🧠 **Core Tech Infrastructure:**

- **Modular insect farming units** (container-based or warehouse setups)
- **AI-driven waste input optimization system** (ensures right nutrient mix)
- **Automated insect lifecycle monitoring** (via IoT sensors and computer vision)
- **Protein processing units** (to convert larvae into feed or human protein)

---

📱 **Digital Platforms:**

- **Waste input management platform** for food producers to schedule and track food waste collection
- **Mobile app for farmers** to access protein feed, pricing, and delivery schedules
- **Blockchain-based traceability system** for ensuring protein quality and origin
- **Data dashboard** for impact metrics (waste diverted, protein produced, $CO_2$ saved)

---

🌍 **Community and Distribution:**

- **Micro-entrepreneurship kits**: empower local operators to run small-scale insect farms
- **Partnerships with food banks & aid orgs** to distribute insect-based protein in high-need areas
- **Educational campaigns** to normalize insect consumption (especially in pilot regions)
- **School nutrition programs** introducing insect protein snacks or powders

---

Would you like to go deeper into any of these sections—like building out a pilot model, unit economics, or the behavioral side (consumer acceptance of insect protein)?

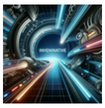

Inventor's Idea Analysis and Business Plan

Prompt 1:

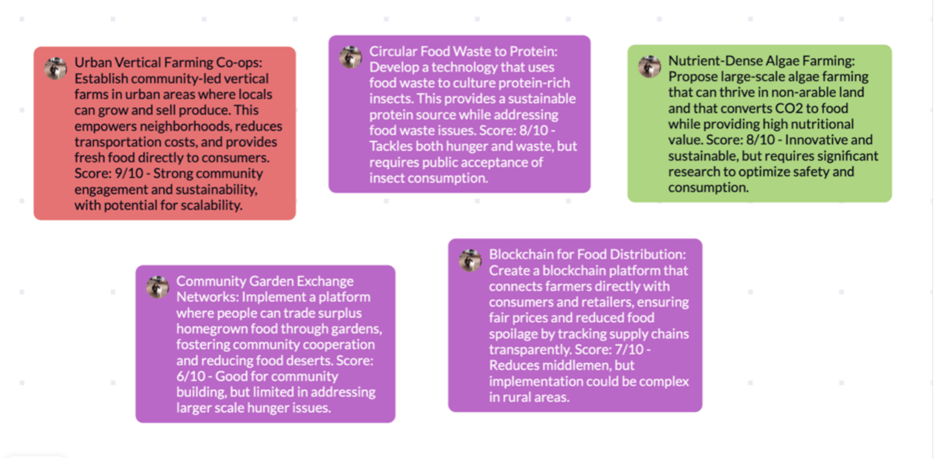


Select the best novel, innovative idea from the proposed ideas for solving Zero Hunger. Please give scores for each novel idea and why it is the best idea.

Result:

Here's an evaluation of the five proposed ideas based on their novelty, scalability, sustainability, and impact potential to solve the "Zero Hunger" goal:

---

## 🥇 Best Idea: Urban Vertical Farming Co-ops

**Score: 9/10**
**Why It's Best:**

- **High Novelty & Community Empowerment:** Establishes a local, scalable solution that allows urban communities to directly grow and sell food.
- **Sustainability:** Reduces transportation emissions, boosts local food resilience.
- **Impact:** Strong potential to alleviate food deserts and empower underserved populations.
- **Scalability:** Easily replicable across urban centers worldwide.

---

## 🟣 Circular Food Waste to Protein

**Score: 8/10**

- **Innovation:** Tackles both food waste and protein deficiency using insects.
- **Challenges:** Requires cultural acceptance of insect-based food; could face regulatory hurdles.
- **Impact Potential:** High for long-term global protein sustainability.

---

## 🟢 Nutrient-Dense Algae Farming

**Score: 8/10**

- **Innovation:** Converts $CO_2$ to food on non-arable land—very forward-thinking.
- **Challenges:** Needs heavy R&D for safe consumption and scalability.
- **Impact Potential:** Very high in addressing nutrition and environmental goals.

---

## 🟪 Blockchain for Food Distribution

**Score: 7/10**

- **Innovation:** Enhances transparency and fairness in food distribution.
- **Challenges:** Complex to implement in rural or underdeveloped areas.
- **Impact:** Reduces food loss and price manipulation, especially in middle-income economies.

---

## 🟪 Community Garden Exchange Networks

**Score: 6/10**

- **Innovation:** Encourages local cooperation and food sharing.
- **Limitations:** Less scalable, more suited to small communities.
- **Impact:** Good for community-building, but not a large-scale hunger solution.

---

## 🔚 Summary

**Winner: Urban Vertical Farming Co-ops**
It strikes the best balance of innovation, feasibility, sustainability, and immediate social

impact. Its community-driven model can be rapidly deployed and scaled, making it a highly effective strategy for addressing Zero Hunger.

Would you like me to develop this into a full business plan using the template?

Prompt 2:

Best Idea for SDG Goal 2, Zero Hunger: Urban Vertical Farming Co-ops

1. What is the concept of your project? (e.g., Urban Vertical Farming Co-ops)

2. Who is your target audience?

3. What are the core features of your project? (e.g., digital platforms, mobile apps, online communities)

Result:

Here's a detailed breakdown of the **best idea** aligned with **SDG Goal 2: Zero Hunger** – **Urban Vertical Farming Co-ops**:

---

### 1. What is the concept of your project?

**Urban Vertical Farming Co-ops** is a community-led initiative that establishes vertical farms in urban areas. These farms are managed and operated by local residents who grow fresh produce using vertical hydroponic or aeroponic systems in repurposed urban spaces (e.g., rooftops, shipping containers, unused lots). The food is then sold or distributed locally, reducing transportation costs, increasing food accessibility, and creating job opportunities within the community.

---

### 2. Who is your target audience?

- **Urban low-income communities** and **food deserts** (areas with limited access to affordable and nutritious food).
- **Local governments and municipalities** aiming to enhance food security and sustainability.
- **Environmental and agricultural NGOs** focused on sustainable food solutions.
- **Urban planners and developers** looking to integrate green infrastructure.
- **Youth and unemployed individuals** seeking employment or skills in agri-tech.

---

### 3. What are the core features of your project?

- 🌱 **Vertical Farming Units:** Modular, space-efficient systems that use hydroponic or aeroponic technologies to grow crops year-round.
- 🧑‍🤝‍🧑 **Community Ownership Model:** Co-operative business structure where locals manage and benefit from the farm's outputs.
- 📱 **Digital Platform:** A mobile and web app for:
  - Farm management (crop tracking, resource use).
  - Community scheduling (volunteering, training).
  - Marketplace integration (local sales, donations).
- 🎓 **Training & Education Programs:** Workshops to teach urban agriculture, sustainability, and entrepreneurship.
- 🌍 **Sustainability Features:** Renewable energy integration (solar panels), water recycling systems, and composting units.
- 🛒 **Local Food Distribution Channels:** Partnerships with schools, local markets, food banks, and restaurants to ensure fresh produce reaches those in need.

---

Would you like to expand this into a pitch deck, full business plan, or prototype roadmap next?

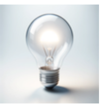

Idea Spark

Prompt 1:

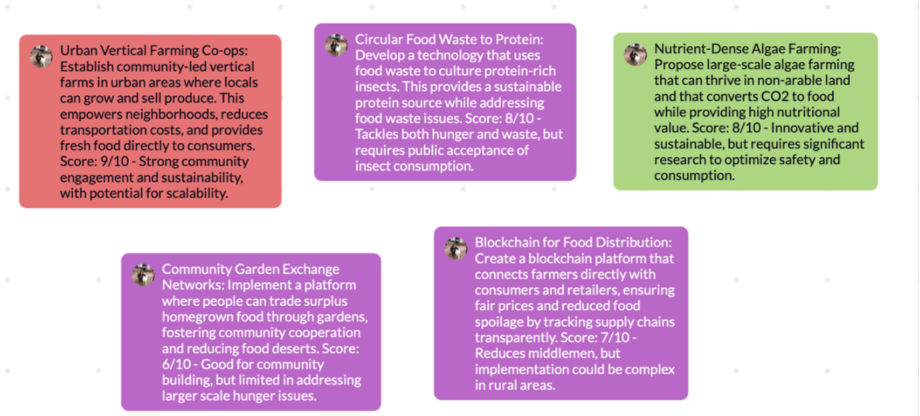


Select the best novel, innovative idea from the proposed ideas for solving Zero Hunger. Please give scores for each novel idea and why it is the best idea.

Result:

Based on the image, let's break down and score each idea for **novelty and innovation** specifically, and then pick the most standout one for solving **Zero Hunger** effectively through a **novel approach**.

---

### 1. Urban Vertical Farming Co-ops

**Innovation Score: 7/10**

- While vertical farming is a growing trend and community-led models add a twist, it's **not entirely novel**.
- However, it's very **scalable and impactful** for urban food deserts.

---

### 2. Circular Food Waste to Protein (Insect Farming)

**Innovation Score: 9/10**

- Tackles **two major issues**: food waste and protein scarcity.
- Highly **novel in approach**, especially using **insects as a sustainable protein** source.
- Public acceptance is a challenge, but the **tech and sustainability angle is cutting-edge**.

---

### 3. Nutrient-Dense Algae Farming

**Innovation Score: 9/10**

- Very **forward-thinking**: turning CO2 and non-arable land into nutritious food via algae.
- Solves hunger **while benefiting the environment**.
- Needs more R&D but has massive **global scalability** potential.

---

### 4. Community Garden Exchange Networks

**Innovation Score: 6/10**

- Nice grassroots concept with local community focus.
- But **not very novel**—essentially a digital swap meet for garden produce.
- Limited in tackling **hunger at scale**.

---

### 5. Blockchain for Food Distribution

**Innovation Score: 7/10**

- Brings tech innovation to supply chains, which is **exciting and modern**.
- But blockchain in agriculture has been **explored already**, and it's more about optimization than new food creation.

---

## 🏆 Top Pick for Best Novel, Innovative Idea:

**Circular Food Waste to Protein (Insect Farming)**

**Why?**

- It hits the **sweet spot** of novelty, sustainability, and addressing **both food waste and malnutrition**.
- Though public perception is a hurdle, the **tech and ecosystem disruption potential** is significant.
- Plus, it aligns with future food trends as demand for **alt-protein sources rises**.

---

Would you like to dive deeper into how this idea could be implemented or adapted for different communities?

Prompt 2:

Best Idea for SDG Goal 2, Zero Hunger: Circular Food Waste to Protein (Insect Farming)

1. What is the concept of your project? (e.g., Circular Food Waste to Protein: Insect Farming)

2. Who is your target audience?

3. What are the core features of your project? (e.g., digital platforms, mobile apps, online communities)

Result:

Absolutely! Here's a solid, concise breakdown tailored to SDG Goal 2: **Zero Hunger** using the *Circular Food Waste to Protein: Insect Farming* concept:

---

## 1. What is the concept of your project?

**Circular Food Waste to Protein: Insect Farming**
This project turns food waste into high-quality, sustainable protein by cultivating insects like black soldier flies or mealworms. The insects are farmed using discarded food scraps and

organic waste, then processed into protein-rich food products for both human and animal consumption. This circular system not only reduces food waste but also provides an affordable, nutrient-dense food source—especially critical in areas struggling with hunger or malnutrition.

---

## 2. Who is your target audience?

- **Low-income and food-insecure communities** (urban and rural)
- **Small-scale farmers and local agricultural cooperatives**
- **Governments and NGOs** tackling food security and sustainability
- **Alternative protein companies and animal feed producers**
- **Eco-conscious consumers** open to sustainable food options

---

## 3. What are the core features of your project?

- 🔄 **Waste Collection Network**: Partners with local food producers, markets, and households to collect organic waste for insect feed.
- 🐛 **Insect Farming Units**: Modular, scalable insect farms (urban or rural), optimized for low-resource environments.
- 📱 **Digital Platform/Mobile App**:
  - Track food waste input and protein output
  - Manage logistics (pickup schedules, supply chain, etc.)
  - Provide data dashboards for impact tracking (waste reduced, protein produced, emissions saved)
- 🌍 **Community Training & Outreach**: Workshops and digital learning modules to teach farming techniques, safety, and acceptance of insect protein.
- 🤝 **Distribution Network**: Channels to deliver the resulting protein (as food or feed) to local markets, schools, or emergency relief organizations.

---

Want help sketching out a name, visual brand, or even a pitch deck for this idea? Let's make it shine!

## Use Case 4: Deploying and Implementing Idea

- **Demonstration of how GenAI tools helped** Deploying and Implementing Idea for Sustainable Development Goal 2 (SDG Goal 2): Zero Hunger

## Deploying and Implementing Ideas

From "There is an AI for that" website Date 26/03/2025 Time 10:30AM
Keywords
1. Prototyping
2. App development

## Illustrations for Deploying and Implementing Ideas

**Table S2.4 Most 3 popular tools for Deploying and Implementing ideas step**

| GenAI | Name | Popularity score | Web link | Description |
|---|---|---|---|---|
| lovable | Lovable | 3,794 | https://lovable.dev/ | Lovable is an AI-powered platform that enables users of any skill level to create full-stack web applications without requiring coding expertise by simply describing what they want in plain English. Instead of hiring developers, users can generate web apps or websites instantly. - taking you from idea to app as fast as possible. |
| Leo™ | Leo AI | 627 | https://app.getleo.ai/ | Leo is presented as the world's pioneering engineering design copilot, that leverages the power of generative AI to enable engineers to convert their ideas into tangible products in a streamlined manner. Leo's AI platform is capable of transforming multiple sources like text, sketches, specifications, and CAD constraints into DFMA-optimized, fully assembled 3D CAD models, thus facilitating a radical shift towards increased efficiency. |
| 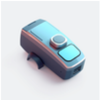 | Prototype App Generator | 138 | https://theresanaiforthat.com/@wandame/prototype-generator/ | Prototype Generator: AI-powered design tool that transforms ideas into visual, innovative product prototypes. Crafts detailed, realistic 3D images with multiple angles, balancing functionality and aesthetics for enhanced usability and appeal. |

Prompt 1:

Absolutely, let's build this out into a sharp, strategic concept aligned with **SDG Goal 2: Zero Hunger**, using a first-principles approach.

---

## ✅ 1. What is the concept of your project?

### Circular Food Waste to Protein: Insect Farming Tech

This project converts **organic food waste** into **nutrient-rich insect protein** using **bioconversion technology**. The insects (e.g., black soldier fly larvae) are farmed in controlled environments and processed into **high-protein animal feed** or **human-grade protein products**, creating a **circular, sustainable, and scalable food system** that addresses both **malnutrition** and **food waste**.

---

## ✅ 2. Who is your target audience?

We segment the audience by both *end users* and *enablers*:

### 🎯 Primary Target Audiences:

- **Undernourished populations** in food-insecure regions (via NGOs or government distribution)
- **Farmers & fishery operators** needing low-cost, sustainable animal feed
- **Low-income urban communities** lacking access to affordable protein

### 🔗 Secondary Stakeholders:

- **Municipal waste management bodies** (as waste suppliers)
- **Governments & development agencies** (as policy/investment partners)
- **Sustainable food startups and social enterprises**
- **Investors in agritech / circular economy ventures**

---

## ✅ 3. What are the core features of your project?

Let's break this into three layers: **Tech Infrastructure**, **Distribution**, and **Community Engagement**

---

### 🧠 Core Tech Infrastructure:

- **Modular insect farming units** (container-based or warehouse setups)
- **AI-driven waste input optimization system** (ensures right nutrient mix)
- **Automated insect lifecycle monitoring** (via IoT sensors and computer vision)
- **Protein processing units** (to convert larvae into feed or human protein)

---

### 📱 Digital Platforms:

- **Waste input management platform** for food producers to schedule and track food waste collection
- **Mobile app for farmers** to access protein feed, pricing, and delivery schedules
- **Blockchain-based traceability system** for ensuring protein quality and origin
- **Data dashboard** for impact metrics (waste diverted, protein produced, $CO_2$ saved)

---

### 🌍 Community and Distribution:

- **Micro-entrepreneurship kits**: empower local operators to run small-scale insect farms
- **Partnerships with food banks & aid orgs** to distribute insect-based protein in high-need areas
- **Educational campaigns** to normalize insect consumption (especially in pilot regions)
- **School nutrition programs** introducing insect protein snacks or powders

Result:

Lovable AI created website from Prompt 1.

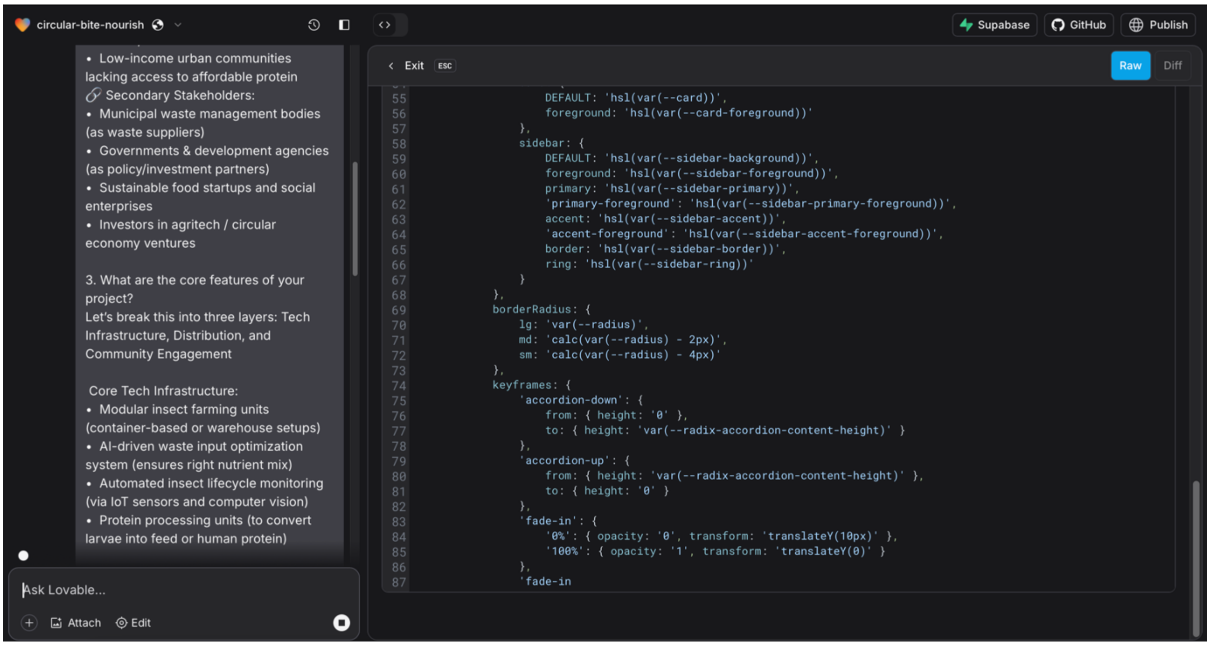

I've created an elegant, minimalist website for your **Circular Food Waste to Protein** project that showcases your innovative technology and impact with beautiful animations and a clean design.

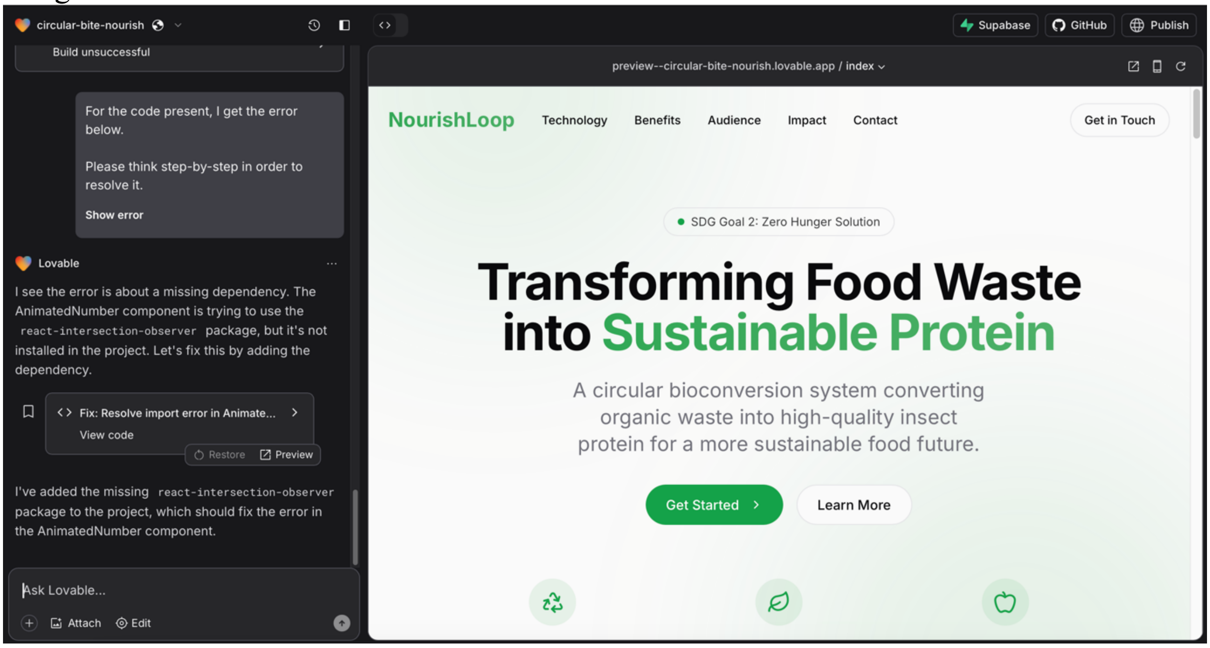

NourishLoop Technology Benefits Audience Impact Contact Get in Touch

SDG Goal 2: Zero Hunger Solution

# Transforming Food Waste into Sustainable Protein

A circular bioconversion system converting organic waste into high-quality insect protein for a more sustainable food future.

Get Started › Learn More

**Circular System**
Converting waste into valuable resources

**Sustainable**
Reducing emissions and conserving resources

**Nutritious**
High-quality protein for humans and animals

## Our Technology

State-of-the-art insect farming and processing technology that transforms organic waste into valuable protein.

**Waste Collection & Processing**
Food waste is collected, sorted, and pre-processed to create the optimal nutritional environment for insect growth.

**AI-Driven Optimization**
Our proprietary algorithms continuously optimize growing conditions and nutritional inputs for maximum efficiency.

**Bioconversion Process**
Black soldier fly larvae convert organic matter into high-quality protein within a controlled, modular environment.

**Protein Processing & Distribution**
Harvested larvae are processed into various forms of protein for animal feed or human consumption and distributed to markets.

Request Demo

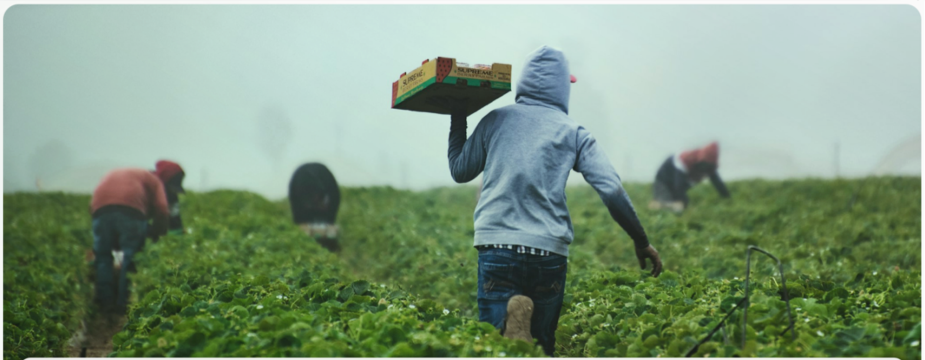

**Modular & Scalable**
Our technology adapts to different scales, from micro-entrepreneurship kits to large industrial installations, making it deployable worldwide.

## Key Benefits

Our circular food system delivers multiple advantages for people and planet, addressing pressing global challenges with an innovative approach.

### Food Security

Provides sustainable protein sources to undernourished populations, helping address malnutrition in food-insecure regions.

### Waste Reduction

Diverts tons of food waste from landfills, reducing methane emissions and environmental impact while creating value.

### Economic Opportunity

Creates jobs and micro-entrepreneurship opportunities in both urban and rural communities through our farming kits.

### Environmental Impact

Significantly lower carbon footprint, water usage, and land requirements compared to traditional protein production methods.

## Who We Serve

Our circular food system creates value for multiple stakeholders across the food value chain, from producers to consumers.

### Undernourished Communities

Working with NGOs and governments to provide high-quality protein to food-insecure regions, addressing malnutrition through sustainable solutions.

### Farmers & Fisheries

Supplying sustainable, cost-effective animal feed that reduces reliance on fishmeal and soy, helping farmers improve their environmental footprint.

### Municipal Waste Managers

Partnering with cities and waste management companies to divert organic waste from landfills, reducing costs and environmental impact.

### Sustainable Food Startups

Collaborating with innovative food companies looking to incorporate insect protein into next-generation sustainable food products.

### Ready to join our ecosystem?

We're building partnerships across the value chain to scale our impact.

Become a Partner

## Our Impact

As we scale our circular food system, the environmental and social impacts grow exponentially.

**1,000** tons
Food waste diverted from landfills annually

**200** tons
High-quality protein produced per year

**95** %
Lower water usage than conventional protein

**2,500+**
People receiving better nutrition

### Our commitment to the SDGs

Beyond Zero Hunger (SDG 2), our solution contributes to Climate Action (SDG 13), Responsible Consumption (SDG 12), and Life on Land (SDG 15).

Learn More About Our Goals

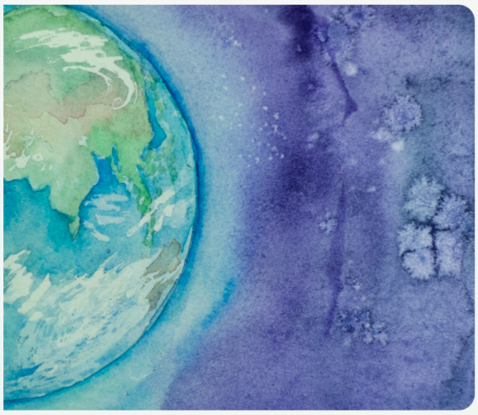

## Get in Touch

Interested in learning more about our technology, becoming a partner, or investing in our mission? We'd love to hear from you.

### Contact Information

Email
contact@nourishloop.com

Phone
+1 (555) 123-4567

Location
San Francisco, CA, USA

### Join Our Mission

We're building a global network of partners committed to addressing food insecurity and environmental challenges through innovative technology.

- Inquire about technology partnerships
- Explore investment opportunities
- Learn about pilot programs

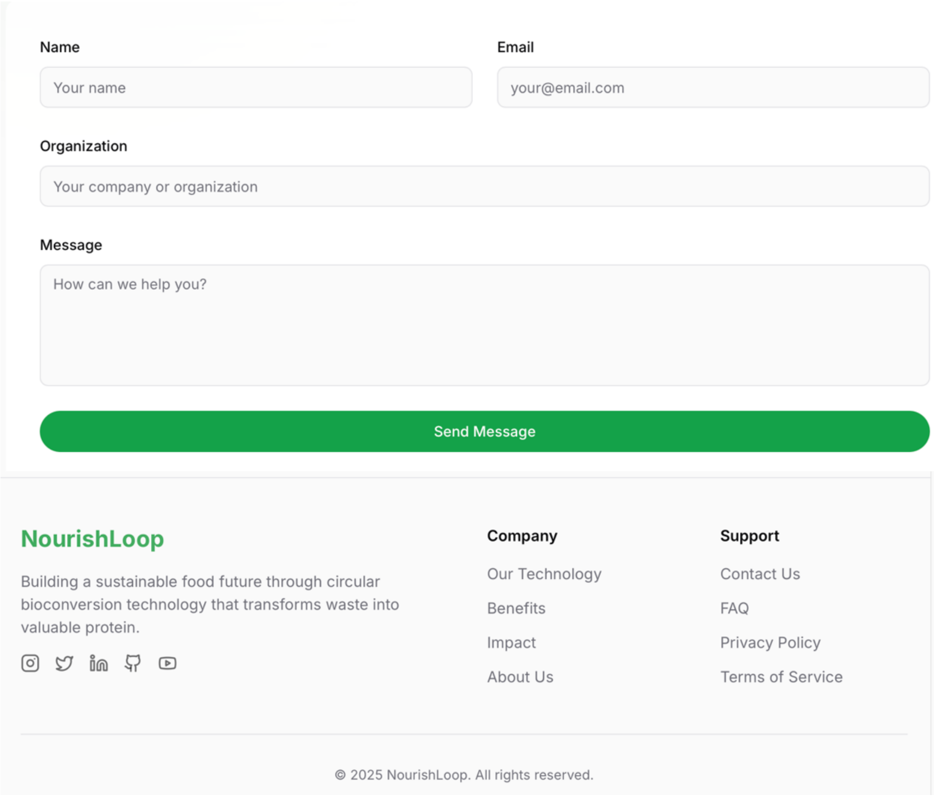


Leo AI Interface:

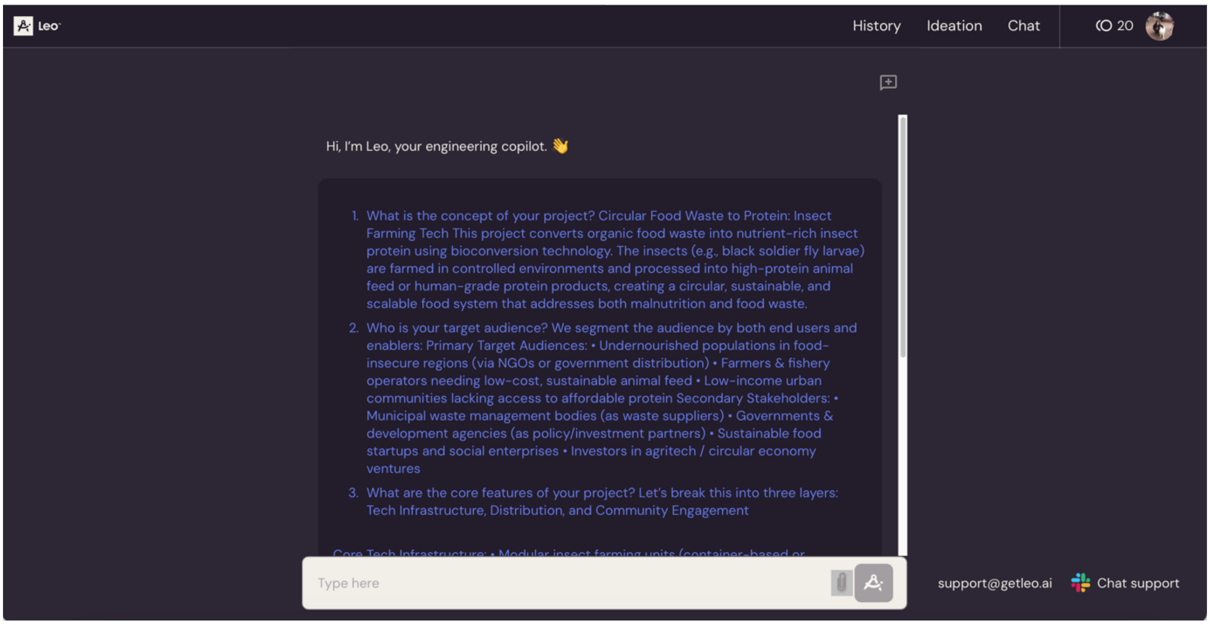

## Primary Target Audiences:

1. **Undernourished populations in food-insecure regions**:
   - These populations can be reached via NGOs or government distribution programs to address malnutrition and food scarcity .
2. **Farmers & fishery operators**:
   - They require low-cost, sustainable animal feed options to support their livestock and aquaculture operations .
3. **Low-income urban communities**:
   - These communities lack access to affordable protein sources, and insect protein can provide a cost-effective alternative .

## Secondary Stakeholders:

1. **Municipal waste management bodies**:
   - These bodies can act as waste suppliers, providing organic food waste for insect farming .
2. **Governments & development agencies**:
   - They can serve as policy and investment partners to support the scalability and sustainability of the project .
3. **Sustainable food startups and social enterprises**:
   - These entities can collaborate to promote and distribute insect-based protein products .
4. **Investors in agritech / circular economy ventures**:
   - Investors can provide the necessary funding and support for the development and expansion of insect farming technologies .

## Digital Platforms for Waste Input Management

- **Waste Input Management Platform**: This platform allows food producers to schedule and track food waste collection. It ensures that waste is collected efficiently and timely, reducing the amount of waste that ends up in landfills .

## Mobile Apps for Farmers

- **Mobile App for Farmers**: This app provides farmers with access to protein feed, pricing, and delivery schedules. It helps farmers manage their feed supplies more effectively and ensures they have the necessary resources for their livestock .

## Blockchain-Based Traceability Systems

- **Blockchain Traceability System**: This system ensures the quality and origin of the protein produced. By using blockchain technology, the system provides a transparent and tamper-proof record of the protein's production journey, from waste collection to final product .

## Data Dashboards for Impact Metrics

- **Data Dashboard**: This dashboard tracks key impact metrics such as the amount of waste diverted, protein produced, and $CO_2$ saved. It provides a comprehensive overview of the project's impact and helps stakeholders monitor progress and make informed decisions .

These digital platforms and systems are crucial for the efficient operation and management of the project, ensuring transparency, efficiency, and sustainability at every step.

To address the core features of your project, I have gathered detailed information on modular insect farming units, AI-driven waste input optimization systems, automated insect lifecycle monitoring, and protein processing units.

## Modular Insect Farming Units

- Modular insect farming units can be designed as container-based or warehouse setups, allowing for scalability and flexibility in deployment .
- These units are equipped with environmental control systems to maintain optimal conditions for insect growth .

## AI-Driven Waste Input Optimization Systems

- AI-driven systems optimize the nutrient mix of the food waste to ensure the right balance for insect growth .
- These systems use machine learning algorithms to analyze the composition of the waste and adjust the input parameters accordingly .

## Automated Insect Lifecycle Monitoring

- IoT sensors and computer vision technologies are used for real-time monitoring of the insect lifecycle .
- These technologies help track various parameters such as temperature, humidity, and growth stages of the insects, ensuring optimal conditions are maintained .

## Protein Processing Units

- Protein processing units convert the harvested larvae into high-protein animal feed or human-grade protein products .
- These units involve processes such as drying, grinding, and packaging to produce the final protein products .

These core features collectively contribute to creating a sustainable and scalable insect farming system that efficiently converts food waste into valuable protein sources.

Best practices for community engagement in agritech projects include:

1. **Developing a Network of Skills and Equipment**:
    - Establish a network of multiple locations that can interact physically and electronically to help each other make things. This network should tap into a

wide range of locations with different skills and resources to better prepare for the future and increasingly rely on themselves .

2. **Face-to-Face Contact**:
   - Bring together key personnel from interfacing organizations, at least for short periods, to enhance the unification and optimization of the workflow and manage the process. This initial face-to-face contact helps establish mutual understanding, trust, and respect, which can then be maintained through virtual communications .
3. **Community Inclusion**:
   - The community should consist of people affected by the products throughout their life cycle. Stakeholders should be prioritized based on their influence on profits due to not meeting their demands. This prioritization helps in creating environmental objectives that align with both stakeholder demands and the values and culture of the manufacturing firm .
4. **Environmental Stewardship**:
   - Demonstrate environmental awareness by creating and publishing an environmental policy, including information about recycled content on packaging, and joining environmental organizations that promote sustainable development and environmental stewardship .
5. **Educational Campaigns**:
   - Implement educational campaigns to normalize insect consumption and introduce insect protein snacks or powders in school nutrition programs. This helps in community acceptance and understanding of the benefits of agritech projects .
6. **Micro-entrepreneurship Kits**:
   - Empower local operators to run small-scale insect farms by providing micro-entrepreneurship kits. This initiative encourages local participation and ownership, fostering a sense of community involvement and sustainability .
7. **Partnerships with Food Banks and Aid Organizations**:
   - Form partnerships with food banks and aid organizations to distribute insect-based protein in high-need areas. This ensures that the benefits of the agritech project reach the most vulnerable populations .

By implementing these best practices, agritech projects can effectively engage the community, ensuring sustainable and inclusive growth.

Bioconversion technology for converting food waste into insect protein involves the use of biological processes to transform organic waste into valuable protein sources. Here are the key aspects of this technology:

1. **Raw Material**: Food waste, including agricultural residues, food processing by-products, and other organic matter, serves as the primary input for the bioconversion process .
2. **Insect Farming**: Insects such as black soldier fly larvae (BSFL) are used for bioconversion. These insects are efficient at converting organic waste into high-protein biomass. The larvae consume the organic waste, and through their digestive processes, they convert it into body mass, which is rich in proteins and fats .
3. **Controlled Environment**: The farming of insects is done in controlled environments to optimize the conditions for their growth and reproduction. This includes

maintaining appropriate temperature, humidity, and aeration levels to ensure the larvae thrive and efficiently convert waste into protein .

4. **Automated Monitoring**: Advanced technologies such as IoT sensors and computer vision are employed to monitor the lifecycle of the insects. These technologies help in tracking the growth, health, and environmental conditions of the insect farming units, ensuring optimal bioconversion efficiency .
5. **Protein Processing**: Once the insects reach the desired stage of growth, they are harvested and processed into protein products. The processing involves drying, grinding, and sometimes further refining to produce high-protein animal feed or human-grade protein products .
6. **Waste Management**: The bioconversion process also addresses waste management issues by diverting organic waste from landfills and converting it into valuable products. The residue from the insect farming process can be used as organic fertilizer, contributing to a circular economy .

The bioconversion technology thus provides a sustainable solution for managing food waste while producing valuable protein products for animal feed and human consumption.

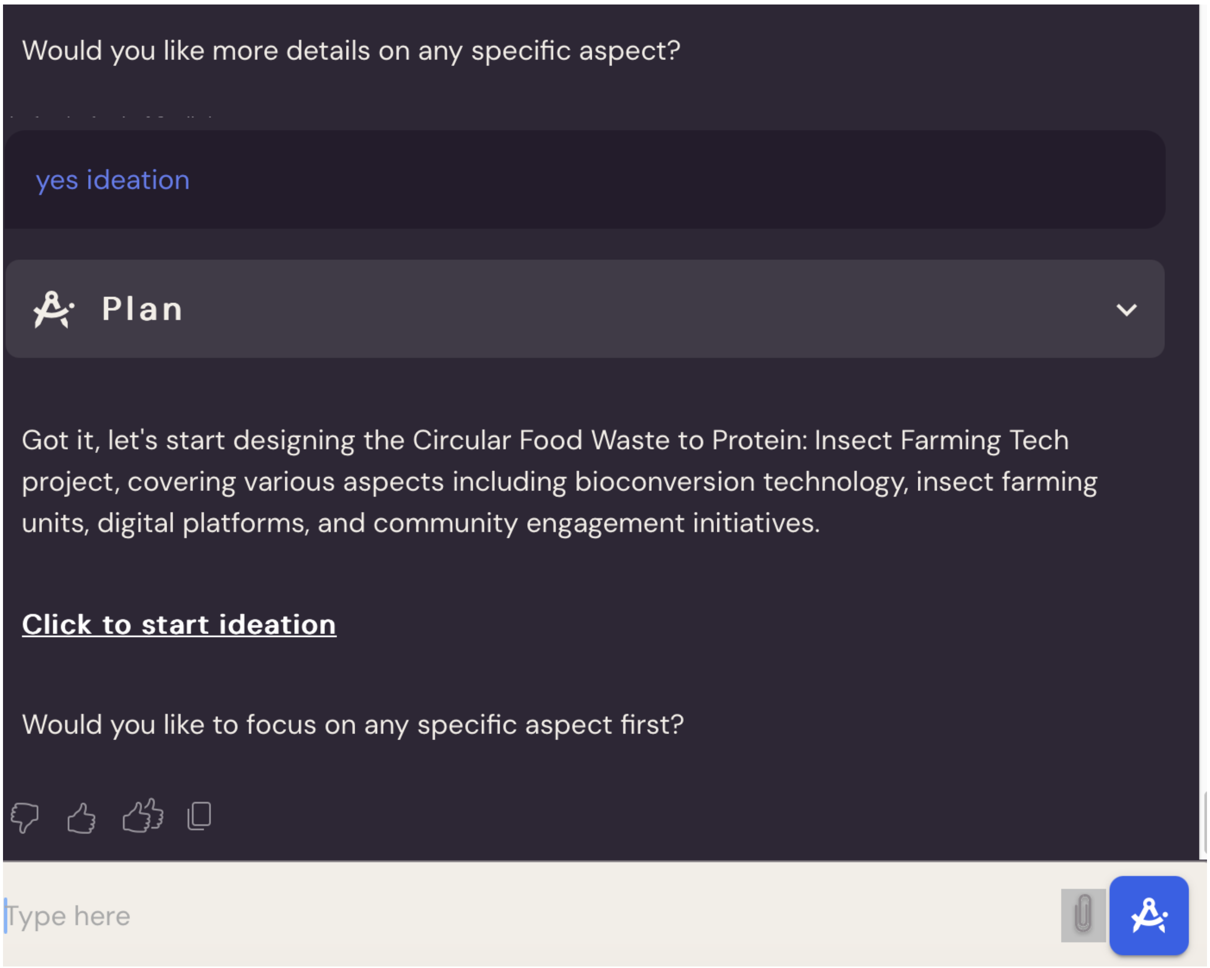

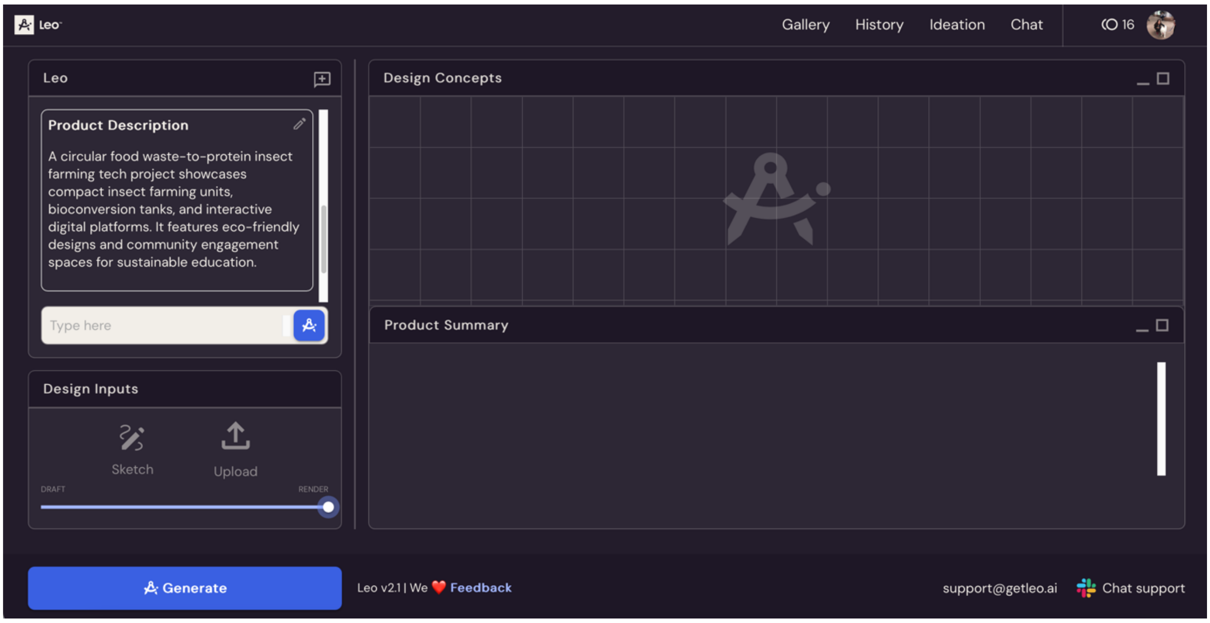
Leo
Gallery
History
Ideation
Chat
16
Leo
Design Concepts
Product Description
A circular food waste-to-protein insect farming tech project showcases compact insect farming units, bioconversion tanks, and interactive digital platforms. It features eco-friendly designs and community engagement spaces for sustainable education.
Type here
Product Summary
Design Inputs
Sketch
Upload
DRAFT
RENDER
Generate
Leo v2.1 | We Feedback
support@getleo.ai
Chat support

Result:

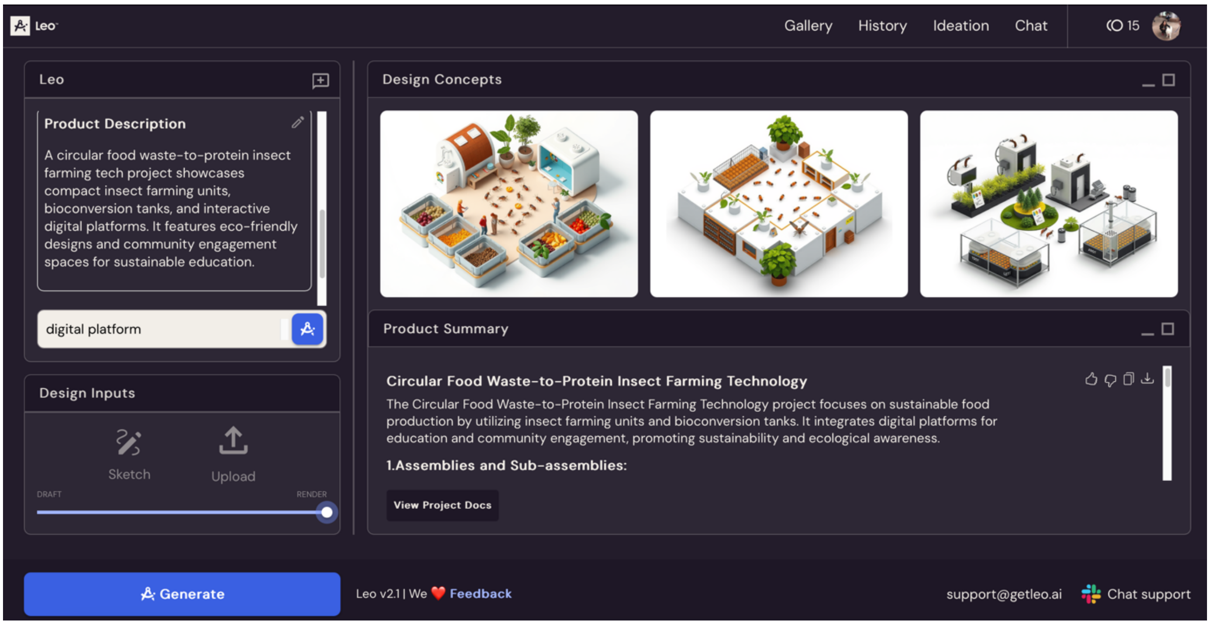
Leo
Gallery
History
Ideation
Chat
15
Leo
Design Concepts
Product Description
A circular food waste-to-protein insect farming tech project showcases compact insect farming units, bioconversion tanks, and interactive digital platforms. It features eco-friendly designs and community engagement spaces for sustainable education.
digital platform
Product Summary
Circular Food Waste-to-Protein Insect Farming Technology
The Circular Food Waste-to-Protein Insect Farming Technology project focuses on sustainable food production by utilizing insect farming units and bioconversion tanks. It integrates digital platforms for education and community engagement, promoting sustainability and ecological awareness.
1.Assemblies and Sub-assemblies:
View Project Docs
Design Inputs
Sketch
Upload
DRAFT
RENDER
Generate
Leo v2.1 | We Feedback
support@getleo.ai
Chat support

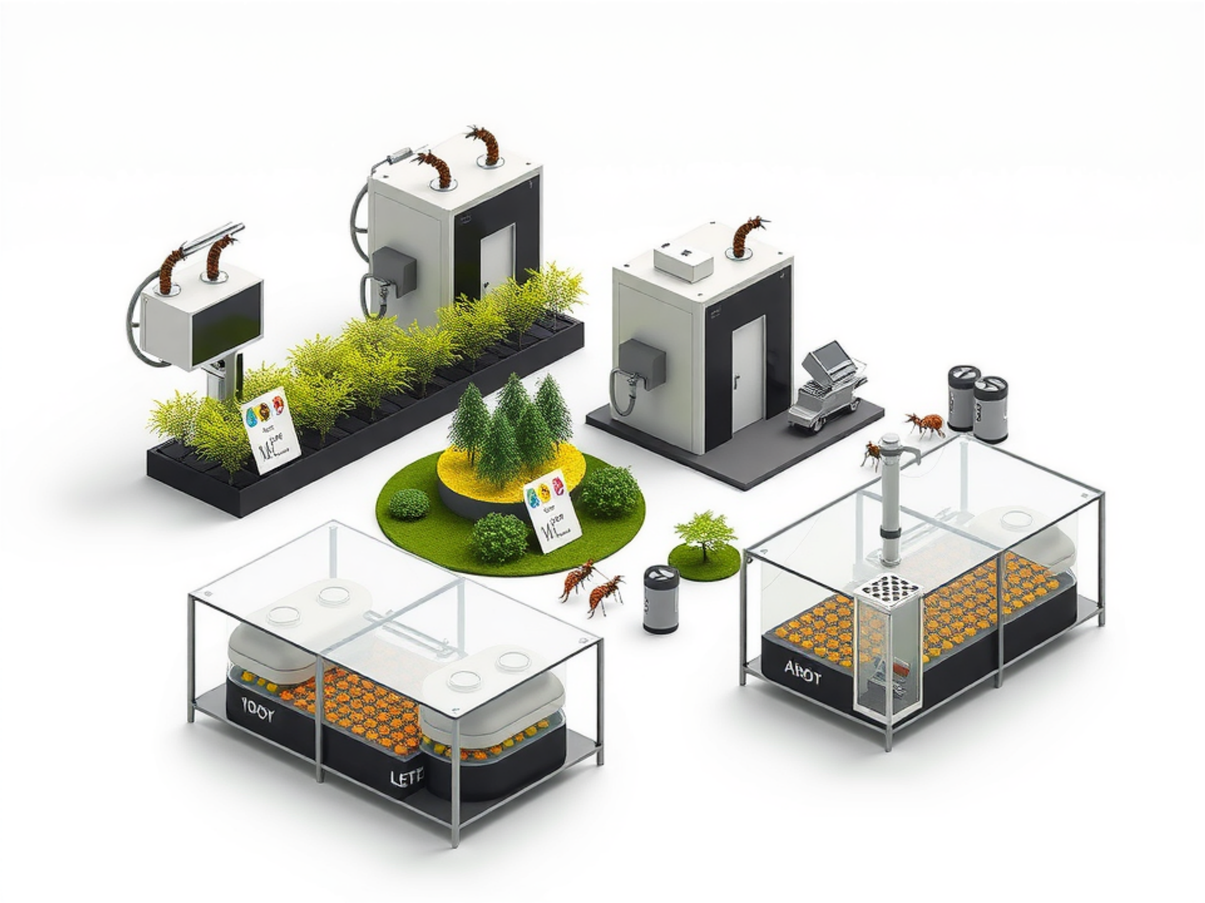

Circular Food Waste-to-Protein Insect Farming Technology

The Circular Food Waste-to-Protein Insect Farming Technology project focuses on sustainable food production by utilizing insect farming units and bioconversion tanks. It integrates digital platforms for education and community engagement, promoting sustainability and ecological awareness.

1.Assemblies and Sub-assemblies:

1.1 Insect Farming Units: Compact structures designed for optimal breeding and nurturing of insect species, with adjustable climate control systems.

1.2 Bioconversion Tanks: Specialized tanks that convert food waste into protein-rich insect biomass through controlled bioconversion processes.

1.3 Digital Interaction Platform: An online platform for education, tracking, and community engagement related to the project, featuring interactive tools and resources.

1.4 Community Engagement Spaces: Design-build areas designed to host workshops, training sessions, and educational events for local communities.

2.Materials and Standards:

2.1 Insect Farming Units: Built with eco-friendly materials such as recycled plastics and breathable mesh for effective air circulation.

2.2 Bioconversion Tanks: Constructed from corrosion-resistant materials, designed to manage organic waste and enhance decomposition.

2.3 Digital Platform: Developed using standard web technologies, ensuring compatibility across devices and compliance with data protection regulations.

2.4 Community Spaces: Designed using sustainable architectural practices, incorporating natural light and renewable energy solutions.

3.Tests and Quality Assurance:

3.1 Bioconversion Efficiency Test: Assessing the effectiveness of waste-to-protein conversion under various conditions.

3.2 Insect Growth Monitoring: Continuous tracking and testing of insect growth rates and health throughout breeding cycles.

3.3 Platform Usability Testing: Evaluating user interaction and satisfaction with the digital platform for educational effectiveness.

3.4 Sustainability Assessment: Regular audits to ensure environmental impacts are minimized throughout the project's lifecycle.

Prototype App Generator

Result:

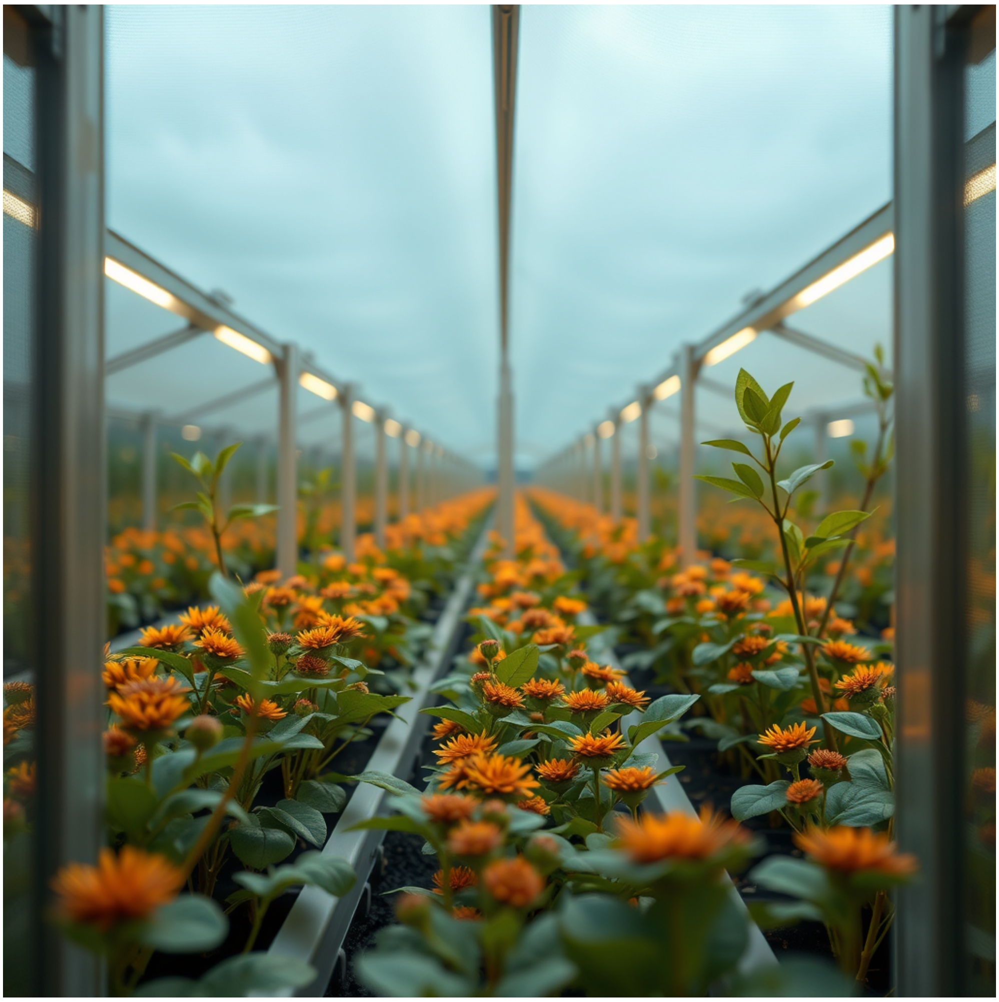

Supplement: Supplementary file 1 [file jintelligence-13-00057-s001.zip › Supplementary material S2 Top 3 popular for each step.pdf]
