# Supplementary material for: AI as a Helper: Leveraging Generative AI Tools Across Common Parts of the Creative Process
Source: J Intell. 2025 May 20;13(5):57. doi: 10.3390/jintelligence13050057 (PMC12112575; doi:10.3390/jintelligence13050057)

# Supplementary material S3

The supplementary material S3 presents the Case of combining GenAI tools for all steps of the creative process. **Date 30/03/2025 Time 17:30 PM**

1. **Demonstration of how GenAI tools helped refine or reframe the problem for** Sustainable Development Goal 2 (SDG Goal 2): Zero Hunger

   **Illustrations for Problem Identification and Framing**

   We use Inquisite as example.

**Prompt 1:** Please identify problem related to SDG Goal 2, Zero Hunger

**Result:**

inquisite

The UN Sustainable Development Goal (SDG) 2 ('Zero Hunger') aims to end all forms of hunger and malnutrition by 2030 (Atukunda et al. 2021). However, several significant problems are hindering progress toward this goal:

Many developing countries, especially African countries, are challenged with extreme hunger that are often caused or compounded by bad governance, conflicts and climate change (Otekunrin et al. 2020). Most African countries have recorded steady reduction in their child mortality rates but high prevalence of undernourishment, stunting and child wasting indicates significant challenges hampering the achievement of the zero hunger target (Otekunrin et al. 2020). Since 2020, the progress towards the achievement of SDG 2 has faced a sudden stall due to an ongoing "polycrisis." While some countries are on track, a great effort is still globally necessary to achieve the SDG2 targets (Sporchia et al. 2024). The mission has been further complicated as the world faced economic turndown due to the outbreak of the COVID-19 virus. These circumstances have brutally affected society's ideal living standards and raised social problems such as extreme poverty, famishment, malnutrition, and medical conditions, specifically among vulnerable children. The progress of zero hunger is decelerated as the pandemic has caused additional challenges (Rahman and Yasin 2022). (Rahman and Yasin 2022). In Pacific Island Countries and Territories, food imports are increasingly common and can provide cheap, although not particularly nutritious foods, leading to generalized health problems. Increased reliance on imports is itself an indicator of diminishing food security (Georgeou et al. 2022). The lack of a systemic approach, together with the complex, global, and nested dimensions of food systems are identified as key elements that need careful consideration when designing sustainability strategies (Sporchia et al. 2024).

**Sources** Sort Export

| Paper | Highlights |
|---|---|
| Unlocking the potential for achievement of the UN Sustainable Development Goal 2 – 'Zero Hunger' – in Africa: targets, strategies, synergies and challenges Open Access<br>Atukunda et al. 2021<br>*Food & Nutrition Research*<br>66 citations<br>Relevancy: | • The paper discusses the need for diverse strategies to achieve UN Sustainable Development Goal 2 ('Zero Hunger') by 2030.<br>• It emphasizes overcoming challenges and creating synergies between various SDG targets to end hunger and malnutrition. |
| Assessing the Zero Hunger Target Readiness in Africa in the Face of COVID-19 Pandemic Open Access<br>Otekunrin et al. 2020<br>*Caraka Tani Journal of Sustainable Agriculture*<br>52 citations<br>Relevancy: | • The paper assesses Africa's readiness to achieve the zero hunger target by 2030, highlighting challenges such as high rates of undernourishment and child wasting despite reductions in child mortality rates.<br>• It recommends that African governments prioritize sustainable agricultural practices and implement policies to combat hunger exacerbated by the COVID-19 pandemic. |
| Food security and small holder farming in Pacific Island countries and territories: A scoping review Open Access<br>Georgeou et al. 2022<br>*PLOS Sustainability and Transformation*<br>20 citations<br>Relevancy: | • The review identifies that smallholder farming is crucial for food security in the Pacific Island Countries and Territories, highlighting themes such as community adaptation and the importance of local food production.<br>• It emphasizes that Pacific peoples adapt quickly to changes by diversifying crops, which supports food security and economic development, particularly for women. |
| Zero hunger: future challenges and the way forward towards the achievement of sustainable development goal 2 Open Access<br>Sporchia et al. 2024<br>*Sustainable Earth Reviews*<br>4 citations<br>Relevancy: | • The paper highlights that progress towards Sustainable Development Goal 2 (Zero Hunger) has stalled due to a global 'polycrisis', necessitating significant global efforts to meet its targets.<br>• Key challenges include the need for a systemic approach and cooperation among various stakeholders to address the complex dimensions of food systems. |
| Children Rights to 'Zero Hunger' and the Execution Challenges during the COVID-19 Crisis<br>Rahman and Yasin 2022<br>*Hasanuddin Law Review*<br>2 citations<br>Relevancy: | • The paper highlights that the COVID-19 pandemic has significantly hindered progress towards the 'zero hunger' goal, exacerbating issues like extreme poverty and malnutrition among vulnerable children.<br>• It recommends urgent humanitarian relief for food distribution and emphasizes the need for governments to comply with Sustainable Development Goal 2 to support at-risk populations. |
| Achieving Zero Hunger by 2030 A Review of Quantitative Assessments of Synergies and Tradeoffs amongst the UN Sustainable Development Goals<br>Valin et al. 2021<br>7 citations<br>Relevancy: | • The paper highlights the complex interrelations between Sustainable Development Goal 2 (SDG2) and other goals, emphasizing the need for joint efforts to address SDG2 alongside Goals 1 and 3 for effective outcomes.<br>• It concludes that achieving SDG2 will require significant transformations in food systems, including efficient production, resource management, and changes in consumption patterns, while also addressing trade-offs with other SDGs. |

2. **Demonstration of how GenAI tools helped idea generation** for Sustainable Development Goal 2 (SDG Goal 2): Zero Hunger

### Illustrations for Generating Ideas

We use Ideamap AI as example.

**Prompt 1:** Create many novel and innovative ideas for Solving SDGs Global challenges as Zero Hunger (Your proposed idea should never be applied somewhere before).

**Result:**

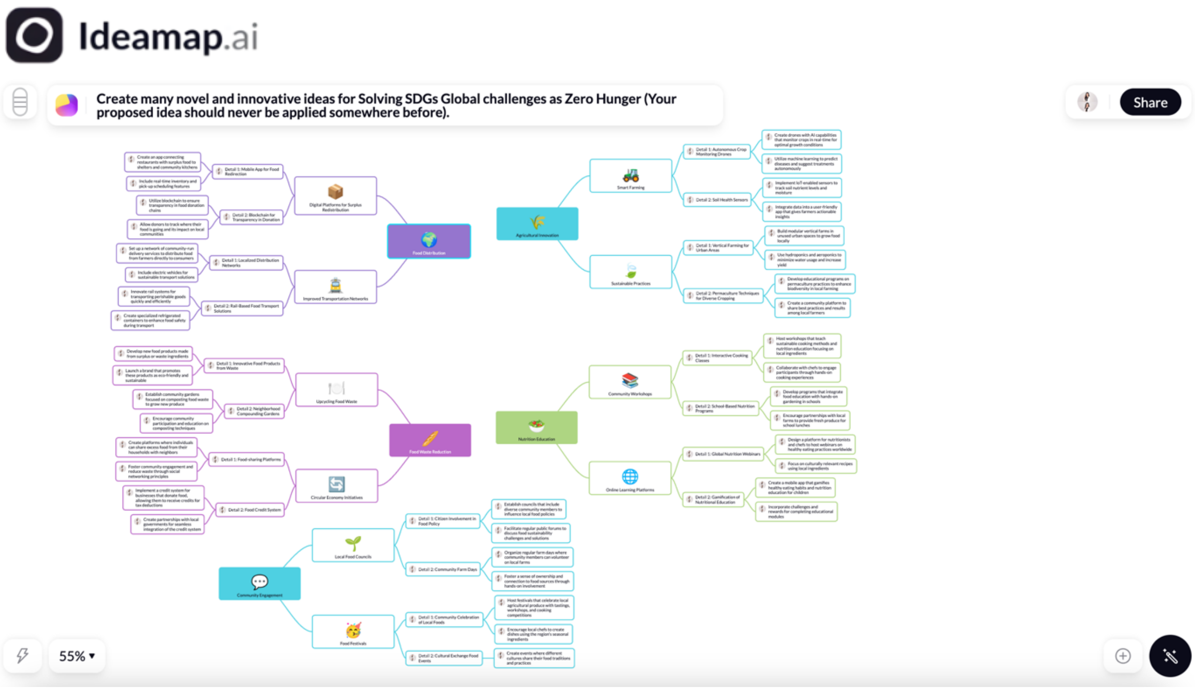

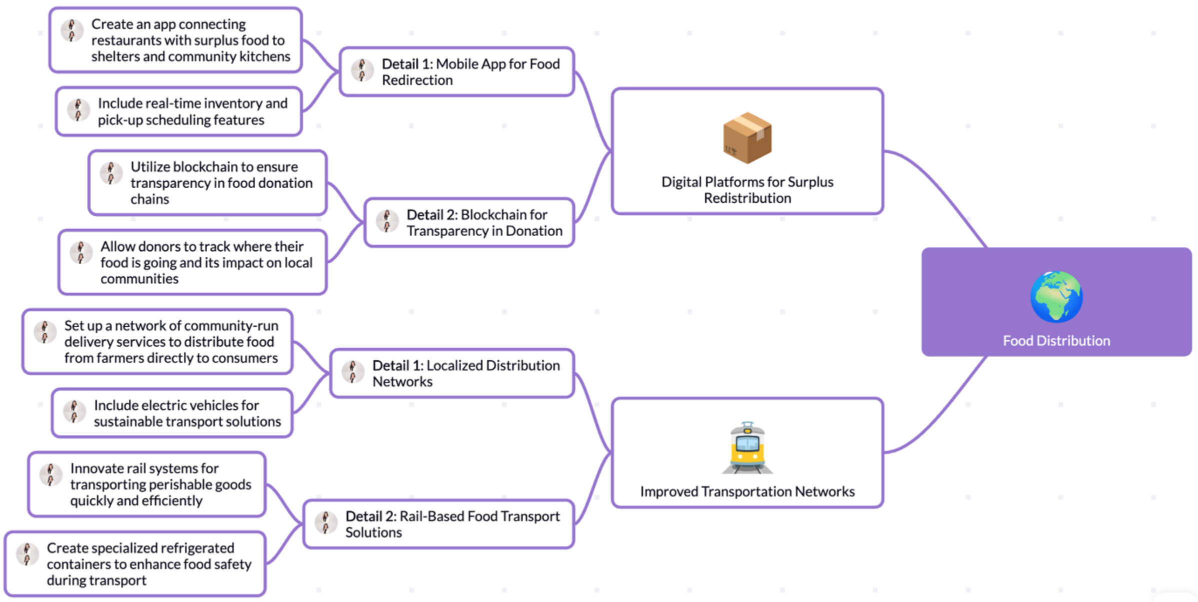
Food Distribution
Digital Platforms for Surplus Redistribution
Detail 1: Mobile App for Food Redirection
Create an app connecting restaurants with surplus food to shelters and community kitchens
Include real-time inventory and pick-up scheduling features
Detail 2: Blockchain for Transparency in Donation
Utilize blockchain to ensure transparency in food donation chains
Allow donors to track where their food is going and its impact on local communities
Improved Transportation Networks
Detail 1: Localized Distribution Networks
Set up a network of community-run delivery services to distribute food from farmers directly to consumers
Include electric vehicles for sustainable transport solutions
Detail 2: Rail-Based Food Transport Solutions
Innovate rail systems for transporting perishable goods quickly and efficiently
Create specialized refrigerated containers to enhance food safety during transport

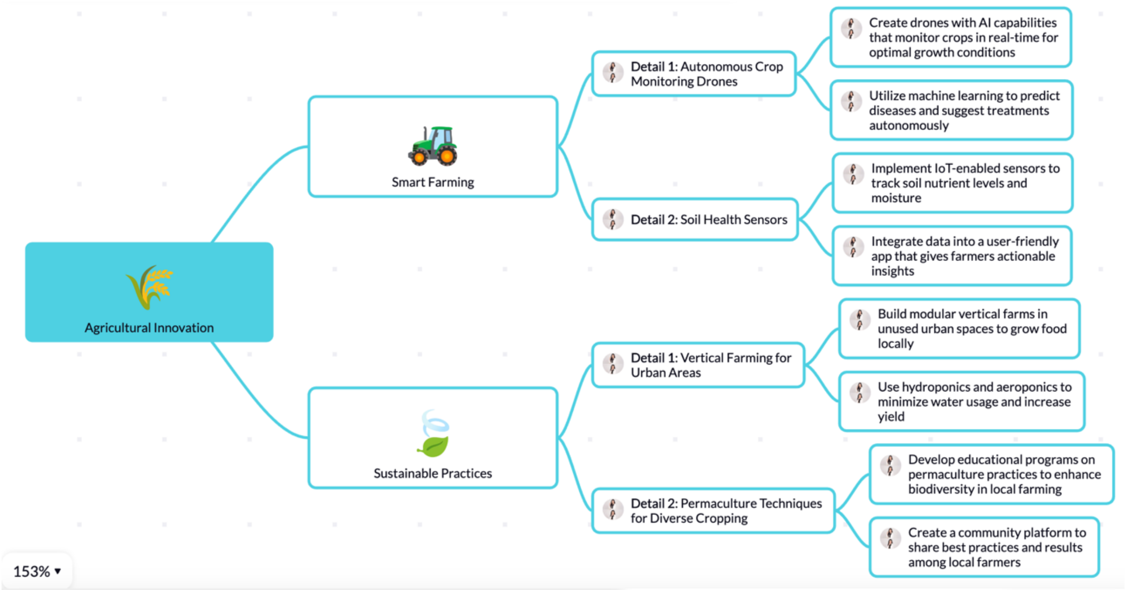
Agricultural Innovation
Smart Farming
Detail 1: Autonomous Crop Monitoring Drones
Create drones with AI capabilities that monitor crops in real-time for optimal growth conditions
Utilize machine learning to predict diseases and suggest treatments autonomously
Detail 2: Soil Health Sensors
Implement IoT-enabled sensors to track soil nutrient levels and moisture
Integrate data into a user-friendly app that gives farmers actionable insights
Sustainable Practices
Detail 1: Vertical Farming for Urban Areas
Build modular vertical farms in unused urban spaces to grow food locally
Use hydroponics and aeroponics to minimize water usage and increase yield
Detail 2: Permaculture Techniques for Diverse Cropping
Develop educational programs on permaculture practices to enhance biodiversity in local farming
Create a community platform to share best practices and results among local farmers
153% ▾

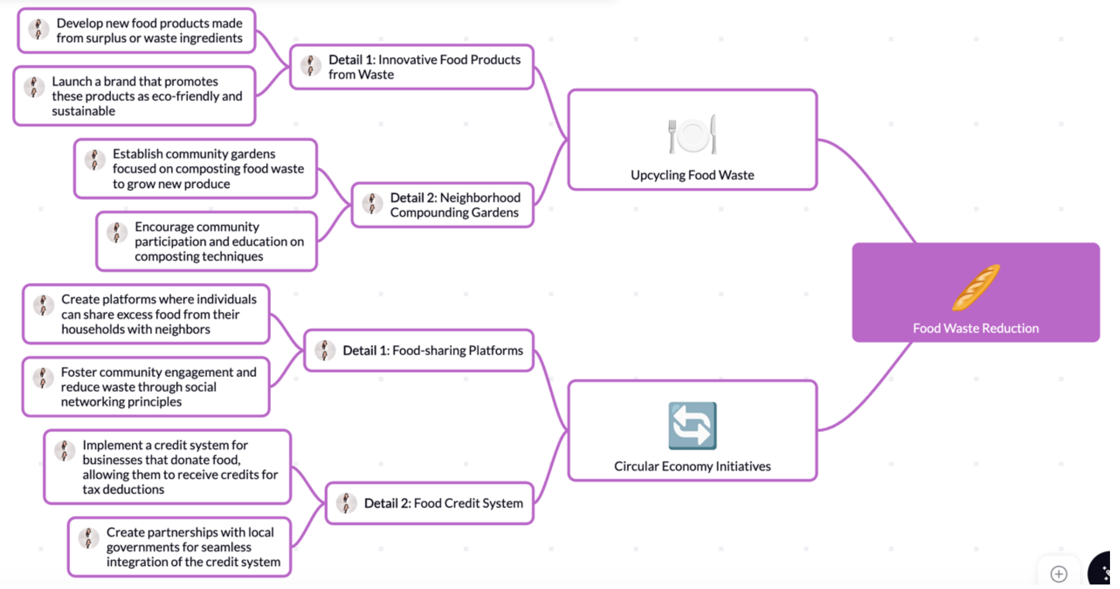
Food Waste Reduction
Upcycling Food Waste
Detail 1: Innovative Food Products from Waste
Develop new food products made from surplus or waste ingredients
Launch a brand that promotes these products as eco-friendly and sustainable
Detail 2: Neighborhood Compounding Gardens
Establish community gardens focused on composting food waste to grow new produce
Encourage community participation and education on composting techniques
Circular Economy Initiatives
Detail 1: Food-sharing Platforms
Create platforms where individuals can share excess food from their households with neighbors
Foster community engagement and reduce waste through social networking principles
Detail 2: Food Credit System
Implement a credit system for businesses that donate food, allowing them to receive credits for tax deductions
Create partnerships with local governments for seamless integration of the credit system

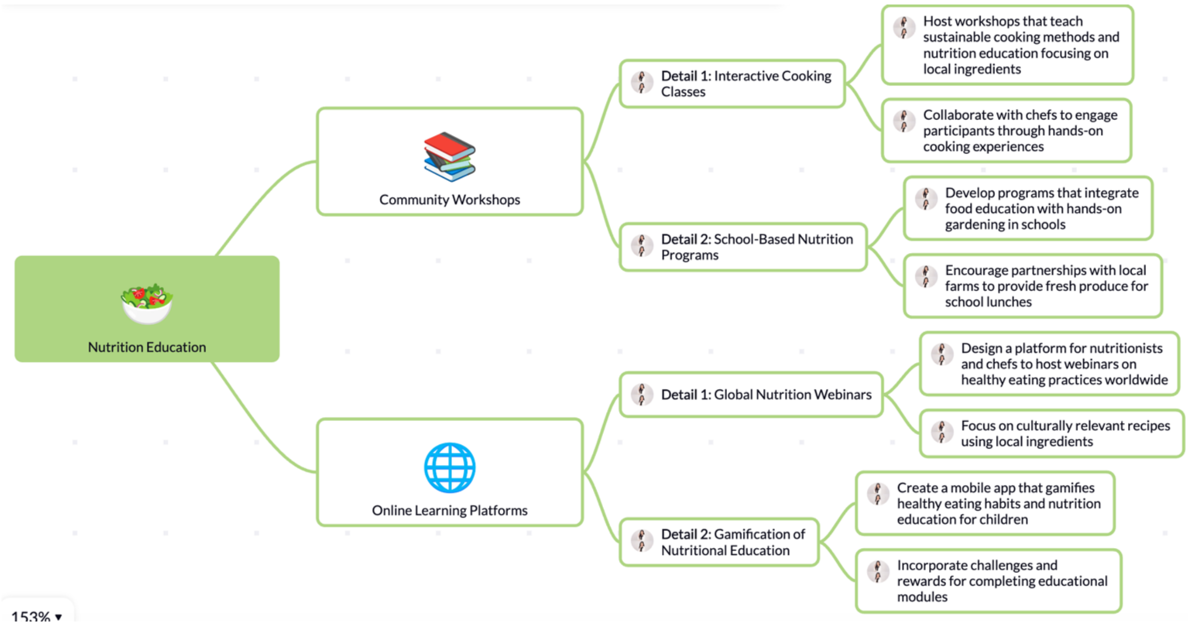
Nutrition Education
Community Workshops
Detail 1: Interactive Cooking Classes
Host workshops that teach sustainable cooking methods and nutrition education focusing on local ingredients
Collaborate with chefs to engage participants through hands-on cooking experiences
Detail 2: School-Based Nutrition Programs
Develop programs that integrate food education with hands-on gardening in schools
Encourage partnerships with local farms to provide fresh produce for school lunches
Online Learning Platforms
Detail 1: Global Nutrition Webinars
Design a platform for nutritionists and chefs to host webinars on healthy eating practices worldwide
Focus on culturally relevant recipes using local ingredients
Detail 2: Gamification of Nutritional Education
Create a mobile app that gamifies healthy eating habits and nutrition education for children
Incorporate challenges and rewards for completing educational modules
153% ▾

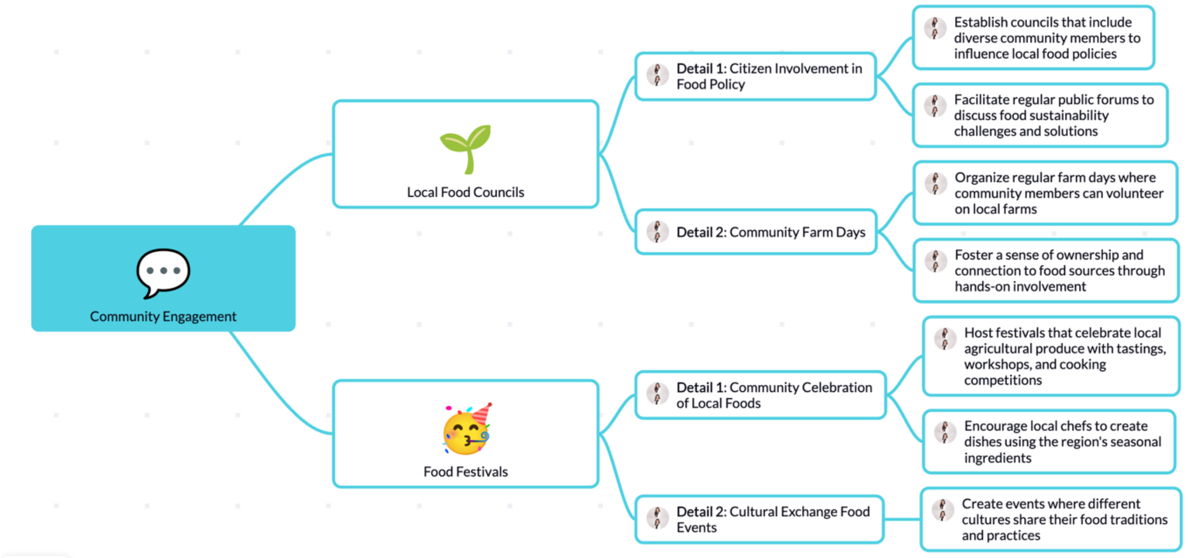
Community Engagement
Local Food Councils
Detail 1: Citizen Involvement in Food Policy
Establish councils that include diverse community members to influence local food policies
Facilitate regular public forums to discuss food sustainability challenges and solutions
Detail 2: Community Farm Days
Organize regular farm days where community members can volunteer on local farms
Foster a sense of ownership and connection to food sources through hands-on involvement
Food Festivals
Detail 1: Community Celebration of Local Foods
Host festivals that celebrate local agricultural produce with tastings, workshops, and cooking competitions
Encourage local chefs to create dishes using the region's seasonal ingredients
Detail 2: Cultural Exchange Food Events
Create events where different cultures share their food traditions and practices

**Prompt 2**: Select the best novel, innovative idea from the proposed ideas for solving Zero Hunger. Please give scores for each novel idea and why it is the best idea.

**Result:**

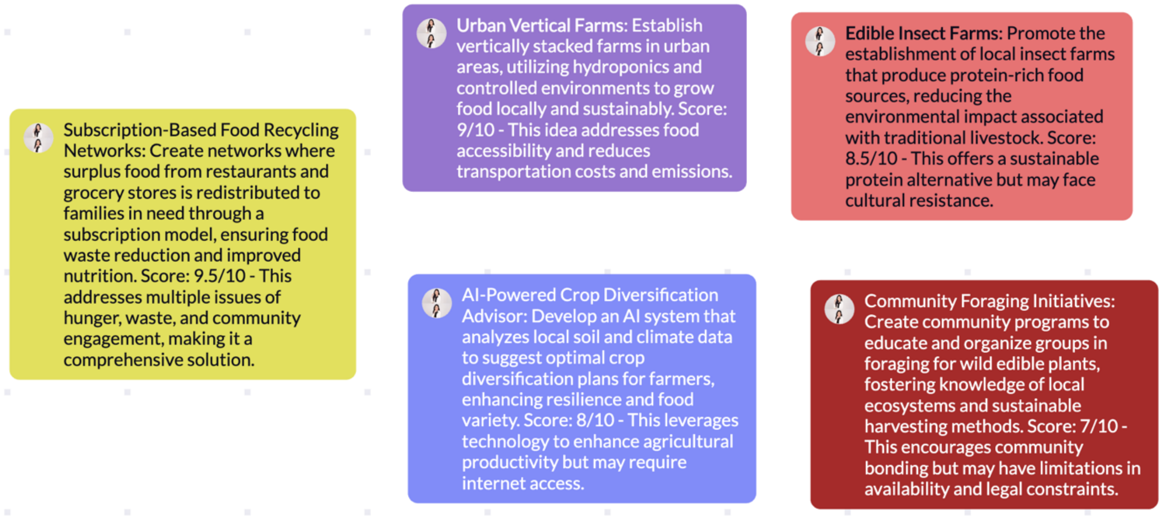

3. **Demonstration of how GenAI tools helped evaluating ideas** for Sustainable Development Goal 2 (SDG Goal 2): Zero Hunger

**Illustrations for Evaluating Ideas**

We use 10X your ideas as example.

**Prompt 1:**

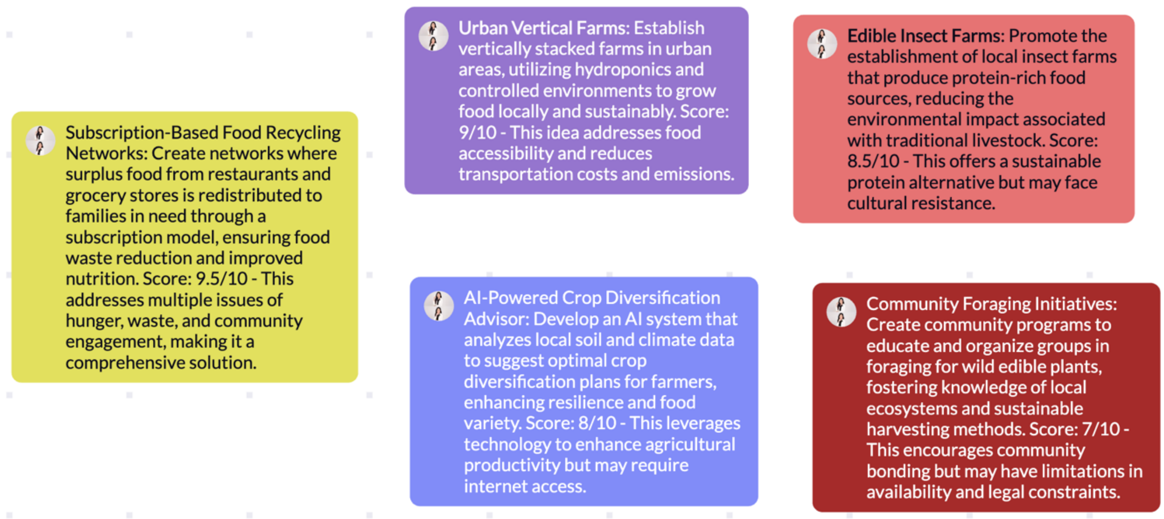


Select the best novel, innovative idea from the proposed ideas for solving Zero Hunger. Please give scores for each novel idea and why it is the best idea.

**Result:**

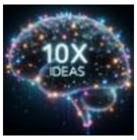


**Evaluating the Ideas for Solving Zero Hunger Based on Novelty, Feasibility, and Impact:**

---

### 📦 1. Subscription-Based Food Recycling Networks

### ✅ Score: 9.5/10

- **Novelty:** Moderate. While food recycling and redistribution models exist (e.g., food banks, apps like Too Good To Go), the subscription model adds a new layer by incentivizing and ensuring consistent distribution to families in need.
- **Impact:** High. Directly reduces food waste while addressing hunger and improving nutrition.

- **Scalability:** High. Easy to replicate in urban and suburban settings, though coordination with local businesses could be a bottleneck.

---

### 🌱 2. Urban Vertical Farms

✅ **Score: 9/10**

- **Novelty:** High. Hydroponic and vertical farming is a rapidly growing field, but integrating it into urban settings to provide affordable, locally grown food enhances its potential.
- **Impact:** High. Improves food accessibility in urban areas while reducing transportation emissions.
- **Scalability:** Moderate. High initial investment and ongoing operational costs may limit expansion, but technology advances may mitigate this.

---

### 🦗 3. Edible Insect Farms

✅ **Score: 8.5/10**

- **Novelty:** High. Producing protein-rich food from insects is a growing trend in sustainability but still faces cultural resistance in many regions.
- **Impact:** Moderate-High. Low resource input and high nutritional value, but widespread adoption could be slow.
- **Scalability:** Moderate. Needs community education and acceptance to scale effectively.

---

### 🤖 4. AI-Powered Crop Diversification Advisor

✅ **Score: 8/10**

- **Novelty:** High. Leveraging AI to analyze soil and climate data for optimal diversification is a forward-thinking approach.
- **Impact:** Moderate. It improves resilience and food security but relies on technology access, which could be a barrier in rural, low-income regions.
- **Scalability:** Moderate. Could be impactful with sufficient infrastructure, but adoption may be slower in underdeveloped areas.

---

### 🌾 5. Community Foraging Initiatives

✅ **Score: 7/10**

- **Novelty:** Moderate. Encouraging foraging and sustainable harvesting is not entirely new but promotes local engagement and education.
- **Impact:** Moderate. Encourages community engagement and knowledge of local ecosystems but may have limited impact on systemic hunger.
- **Scalability:** Low. Difficult to scale broadly due to regional availability and legal limitations.

---

🎯 **Best Innovative Idea: Subscription-Based Food Recycling Networks**

✅ **Why It's the Best Idea:**

- **Immediate Impact:** Addresses hunger by redistributing surplus food effectively while reducing food waste.
- **Community Engagement:** Involves local businesses and households, creating a sustainable ecosystem.
- **Scalability:** Can be easily expanded with a subscription model that ensures consistent delivery.

👉 **Recommendation:** Focus on refining the logistics, ensuring seamless supply chain management, and integrating real-time tracking to measure impact.

**Prompt 2:**

Best Idea for SDG Goal 2, Zero Hunger: **Subscription-Based Food Recycling Networks**
1. What is the concept of your project? (e.g., **Subscription-Based Food Recycling Networks**)
2. Who is your target audience?
3. What are the core features of your project? (e.g., digital platforms, mobile apps, online communities)

**Result:**

🎯 **Best Idea for SDG Goal 2: Zero Hunger – Subscription-Based Food Recycling Networks**

---

**1 What is the Concept of Your Project?**

**Subscription-Based Food Recycling Networks** aim to reduce food waste and combat hunger by redistributing surplus food from restaurants, grocery stores, and food producers to families in need.

- Through a **subscription model**, households and donors can contribute a recurring fee that helps sustain the logistics, operations, and technology required to ensure continuous redistribution of excess food.
- The model ensures **consistent, predictable delivery** of nutritious food, creating a win-win for reducing waste while improving food security in vulnerable communities.

---

**2 Who is Your Target Audience?**

**Primary Beneficiaries:**

- Low-income families and individuals experiencing food insecurity.
- Vulnerable communities, including the elderly, homeless, and marginalized populations.

**Food Donors/Partners:**

- Restaurants, grocery stores, and food manufacturers with surplus perishable and non-perishable items.
- Farmers with excess produce that may otherwise go unsold or wasted.

**Subscribers/Supporters:**

- Individuals and families who subscribe to sponsor food redistribution, contributing monthly/annually.
- Corporate sponsors, CSR initiatives, and local businesses looking to support a social cause.

**Government and Nonprofits:**

- Municipalities and NGOs working towards food security and waste reduction, who could leverage this system for public welfare.

---

**3 What are the Core Features of Your Project?**

**Core Features Include:**

**1. Digital Platform (Web & Mobile App)**

- **Donor Interface:** Allows food providers to list surplus inventory, schedule pickups, and receive tax incentives for donations.
- **Recipient Interface:** Enables families to sign up for a subscription plan that delivers food to their homes or community centers.
- **Logistics Coordination:** Matches available surplus food with recipients efficiently through automated routing and inventory management.

---

**2. Subscription Model for Sustainability**

- **Tiered Subscription Plans:** Allow donors and supporters to subscribe at different tiers to sponsor various levels of food distribution.
- **Crowdsourced Funding:** Engages individuals, businesses, and communities to sustain operational costs through recurring contributions.

---

**3. Smart Inventory & Food Matching System**

- **AI-Powered Matching Algorithm:** Ensures that donated food matches the dietary and cultural preferences of recipients, minimizing waste.
- **Real-time Tracking & Impact Analytics:** Provides transparency by tracking donations, deliveries, and impact metrics to ensure accountability.

---

**4. Community Engagement & Awareness**

- **Volunteer and Engagement Platform:** Mobilizes local volunteers for last-mile delivery and educates communities on food security.
- **Educational Content & Workshops:** Teaches participants about food sustainability, nutrition, and minimizing food waste.

---

**5. Partnerships and Incentives**

- **Corporate & Government Partnerships:** Collaborates with CSR programs, government agencies, and environmental groups to scale efforts.
- **Tax Benefits for Donors:** Provides financial incentives for businesses that donate surplus food.

---

💡 **Next Steps:** Develop an MVP (minimum viable product) focused on high-need areas, followed by expansion into urban and rural regions to maximize impact.

4. **Demonstration of how GenAI tools helped Deploying and Implementing ideas** for Sustainable Development Goal 2 (SDG Goal 2): Zero Hunger

**Illustrations for Deploying and Implementing Ideas**

We use Leo AI and Prototype App Generator as example.

**Prompt 1:**

**1 What is the Concept of Your Project?**

**Subscription-Based Food Recycling Networks** aim to reduce food waste and combat hunger by redistributing surplus food from restaurants, grocery stores, and food producers to families in need.

- Through a **subscription model**, households and donors can contribute a recurring fee that helps sustain the logistics, operations, and technology required to ensure continuous redistribution of excess food.
- The model ensures **consistent, predictable delivery** of nutritious food, creating a win-win for reducing waste while improving food security in vulnerable communities.

**2 Who is Your Target Audience?**

**Primary Beneficiaries:**

- Low-income families and individuals experiencing food insecurity.
- Vulnerable communities, including the elderly, homeless, and marginalized populations.

**Food Donors/Partners:**

- Restaurants, grocery stores, and food manufacturers with surplus perishable and non-perishable items.
- Farmers with excess produce that may otherwise go unsold or wasted.

**Subscribers/Supporters:**

- Individuals and families who subscribe to sponsor food redistribution, contributing monthly/annually.
- Corporate sponsors, CSR initiatives, and local businesses looking to support a social cause.

**Government and Nonprofits:**

- Municipalities and NGOs working towards food security and waste reduction, who could leverage this system for public welfare.

**3 What are the Core Features of Your Project?**

**Core Features Include:**

**1. Digital Platform (Web & Mobile App)**

- **Donor Interface:** Allows food providers to list surplus inventory, schedule pickups, and receive tax incentives for donations.
- **Recipient Interface:** Enables families to sign up for a subscription plan that delivers food to their homes or community centers.
- **Logistics Coordination:** Matches available surplus food with recipients efficiently through automated routing and inventory management.

**2. Subscription Model for Sustainability**

- **Tiered Subscription Plans:** Allow donors and supporters to subscribe at different tiers to sponsor various levels of food distribution.
- **Crowdsourced Funding:** Engages individuals, businesses, and communities to sustain operational costs through recurring contributions.

**3. Smart Inventory & Food Matching System**

- **AI-Powered Matching Algorithm:** Ensures that donated food matches the dietary and cultural preferences of recipients, minimizing waste.
- **Real-time Tracking & Impact Analytics:** Provides transparency by tracking donations, deliveries, and impact metrics to ensure accountability.

**4. Community Engagement & Awareness**

- **Volunteer and Engagement Platform:** Mobilizes local volunteers for last-mile delivery and educates communities on food security.
- **Educational Content & Workshops:** Teaches participants about food sustainability, nutrition, and minimizing food waste.

**5. Partnerships and Incentives**

- **Corporate & Government Partnerships:** Collaborates with CSR programs, government agencies, and environmental groups to scale efforts.
- **Tax Benefits for Donors:** Provides financial incentives for businesses that donate surplus food.

**Result:**

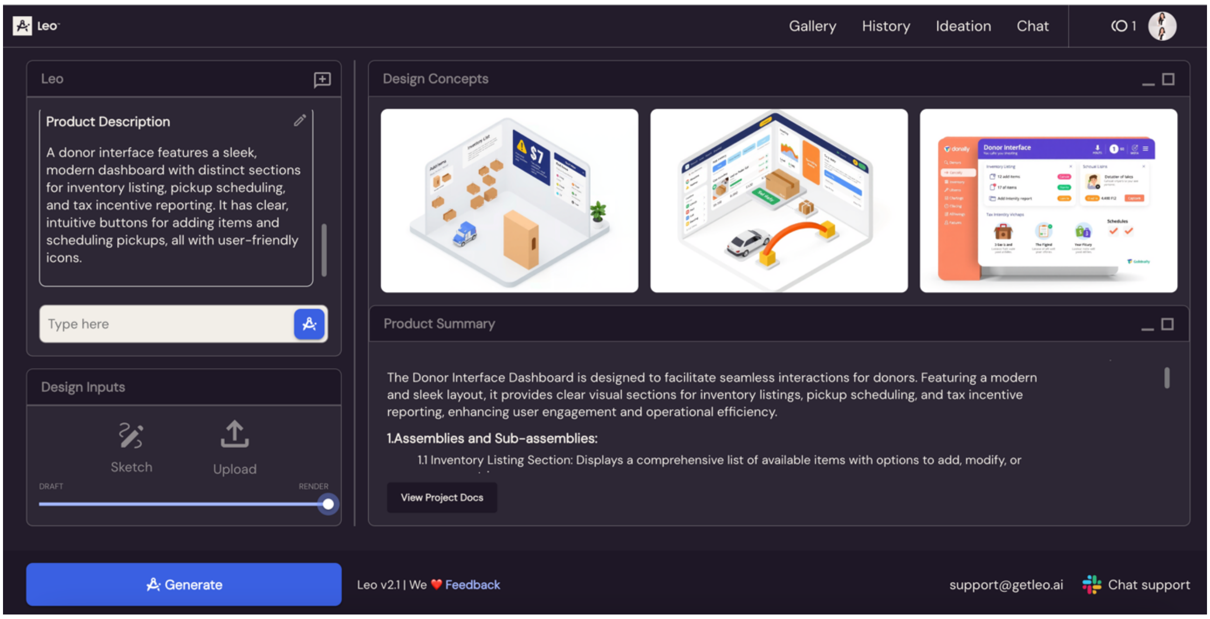
Leo
Gallery
History
Ideation
Chat
Leo
Product Description
A donor interface features a sleek, modern dashboard with distinct sections for inventory listing, pickup scheduling, and tax incentive reporting. It has clear, intuitive buttons for adding items and scheduling pickups, all with user-friendly icons.
Type here
Design Inputs
Sketch
Upload
DRAFT
RENDER
Design Concepts
Product Summary
The Donor Interface Dashboard is designed to facilitate seamless interactions for donors. Featuring a modern and sleek layout, it provides clear visual sections for inventory listings, pickup scheduling, and tax incentive reporting, enhancing user engagement and operational efficiency.
1.Assemblies and Sub-assemblies:
1.1 Inventory Listing Section: Displays a comprehensive list of available items with options to add, modify, or
View Project Docs
Generate
Leo v2.1 | We ❤ Feedback
support@getleo.ai
Chat support

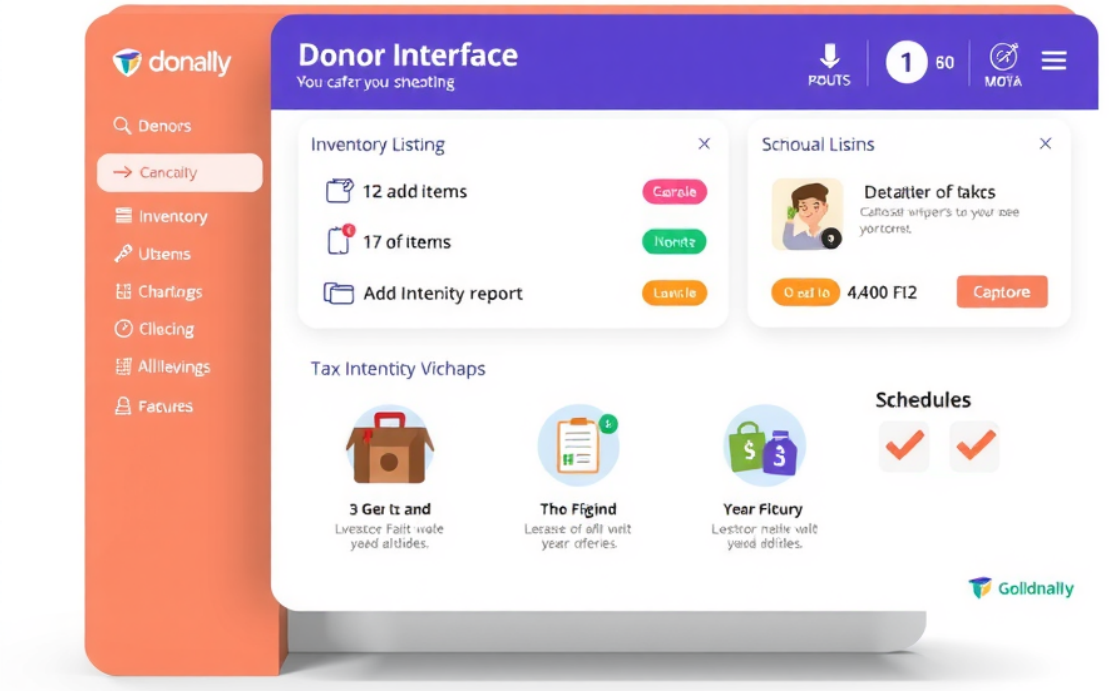
donally
Donor Interface
You cafer you sheating
POUTS
1
60
MOYA
Denors
Cancaity
Inventory
Utems
Chartogs
Cllecing
Alllevings
Facures
Inventory Listing
12 add items
17 of items
Add Intenity report
Schoual Lisins
Detaitler of takcs
4,400 F12
Captore
Tax Intentity Vichaps
Schedules
3 Ger tt and
Tho Ffgind
Year Ficury
Golldnally

**Donor Interface Dashboard**

The Donor Interface Dashboard is designed to facilitate seamless interactions for donors. Featuring a modern and sleek layout, it provides clear visual sections for inventory listings, pickup scheduling, and tax incentive reporting, enhancing user engagement and operational efficiency.

1.Assemblies and Sub-assemblies:

1.1 Inventory Listing Section: Displays a comprehensive list of available items with options to add, modify, or remove entries.

1.2 Pickup Scheduling Section: Allows users to select preferred dates and times for item pickups using a calendar interface.

1.3 Tax Incentive Reporting Section: Provides detailed summaries of donations for tax reporting purposes, with easy export options.

1.4 User Navigation Module: Intuitive button layout with user-friendly icons for smooth navigation throughout the dashboard.

2.Materials and Standards:

2.1 Interface Design: Developed using web technologies such as HTML, CSS, and JavaScript for compatibility and responsiveness.

2.2 Icons and Visual Elements: Created using scalable vector graphics (SVG) for clarity at any resolution.

2.3 Standards: Designed in accordance with user experience (UX) best practices and accessibility standards to ensure inclusive usability.

3.Tests and Quality Assurance:

3.1 Usability Testing: Conducted with real users to evaluate the intuitiveness of the layout and interactions.

3.2 Functionality Testing: Ensures all features work as expected, including adding items and scheduling pickups.

3.3 Cross-Browser Testing: Validated compatibility across multiple web browsers and devices.

3.4 Performance Testing: Assesses loading times and responsiveness under various user loads.

**Prompt 1:** A donor interface features a clean, user-friendly dashboard with sections for inventory listing, pickup scheduling, and tax incentive reporting. It includes buttons for adding items, scheduling pickups, and accessing support resources, all designed with intuitive navigation and clear icons.

Result:

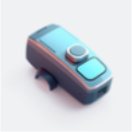

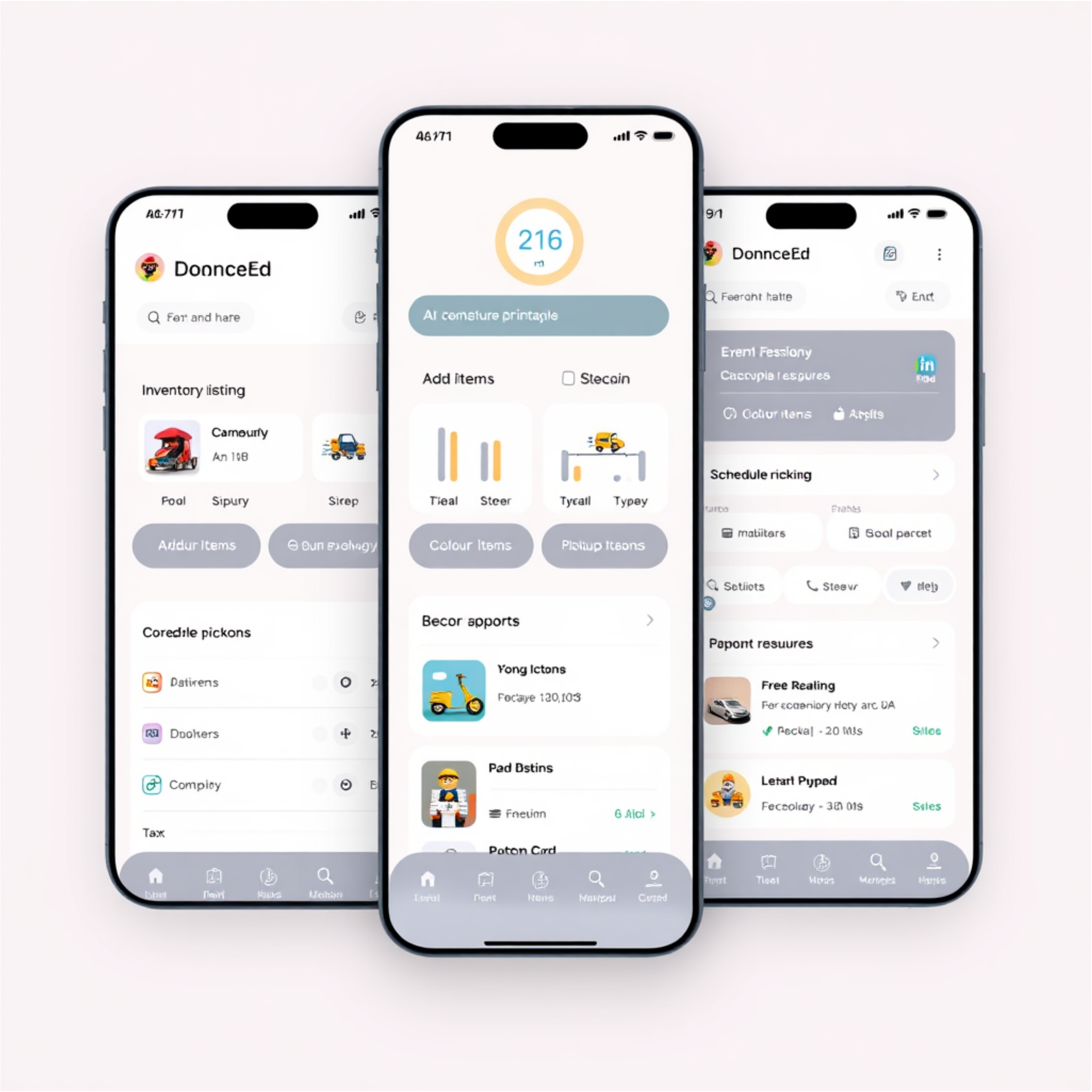

Supplement: Supplementary file 1 [file jintelligence-13-00057-s001.zip › Supplementary material S3 Combining Case of GenAIs.pdf]
